# Programming Multistable Metamaterials to Discover Latent Functionalities

**DOI:** 10.1002/advs.202202883

**Published:** 2022-10-17

**Authors:** Hossein Mofatteh, Benyamin Shahryari, Armin Mirabolghasemi, Alireza Seyedkanani, Razieh Shirzadkhani, Gilles Desharnais, Abdolhamid Akbarzadeh

**Affiliations:** ^1^ Department of Bioresource Engineering McGill University Montreal QC H9X 3V9 Canada; ^2^ Axis Prototypes Montreal QC H1P 3C1 Canada; ^3^ Department of Mechanical Engineering McGill University Montreal QC H3A 0C3 Canada

**Keywords:** continuous path, mechanical memory/sensor, multistable chain, programmable metamaterial, tunable chiral metamaterial

## Abstract

Using multistable mechanical metamaterials to develop deployable structures, electrical devices, and mechanical memories raises two unanswered questions. First, can mechanical instability be programmed to design sensors and memory devices? Second, how can mechanical properties be tuned at the post‐fabrication stage via external stimuli? Answering these questions requires a thorough understanding of the snapping sequences and variations of the elastic energy in multistable metamaterials. The mechanics of deformation sequences and continuous force/energy–displacement curves are comprehensively unveiled here. A 1D array, that is chain, of bistable cells is studied to explore instability‐induced energy release and snapping sequences under one external mechanical stimulus. This method offers an insight into the programmability of multistable chains, which is exploited to fabricate a mechanical sensor/memory with sampling (analog to digital‐A/D) and data reconstruction (digital to analog‐D/A) functionalities operating based on the correlation between the deformation sequence and the mechanical input. The findings offer a new paradigm for developing programmable high‐capacity read–write mechanical memories regardless of thei size scale. Furthermore, exotic mechanical properties can be tuned by harnessing the attained programmability of multistable chains. In this respect, a transversely multistable mechanical metamaterial with tensegrity‐like bistable cells is designed to showcase the tunability of chirality.

## Graphical abstract

Multistability in mechanics is a characteristic that provides distinct stable shapes and consequently imparts multiple functionalities to materials. Shape transformations in multistable metamaterials, triggered by elastic instability of constitutive cells, have sequential traits and are exploited to release elastic energy during reconfiguration. Programming these metamaterials is a new avenue in realizing mechanical sensors/memories, impact shields, and soft jumpers.

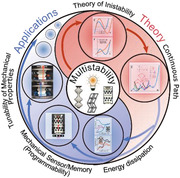

## 1 Introduction

Inspiration from nature^[^ ^1^, ^2^, ^3^, ^4^, ^5^, ^6^ ^]^ is an important key to developing science and forming the sprouts of today and future engineering manifest. Besides nature, advancement in other fields of science can be a root of inspiration.^[^ ^7^, ^8^ ^]^ Underlying energy release mechanisms of rapid snapping Venus flytrap to capture an insect,^[^ ^2^, ^9^ ^]^ cheetahs' spine during high‐speed galloping,^[^ ^10^, ^11^ ^]^ distinctive deformation sequences in earwig wing,^[^ ^12^ ^]^ human digestive organs,^[^ ^13^ ^]^ and reconfigurable traits of shape memory alloys^[^ ^14^ ^]^ are a few spectacular examples of mechanisms and materials that emanate their functionalities mainly from the snapping structural instability and deformation sequences. Elastic released energy and shape‐reconfigurability have been the subject of recent studies.^[^ ^15^, ^16^, ^17^ ^]^ However, two challenges are yet to be addressed: first, programming the released energy by controlling the instability forces; and second, predicting and controlling the deformation sequences to leverage all attainable configurations of a material/structure by applying stimuli merely at a single point on its exterior boundary. In addition, metamaterials are commonly topologically designed in the pre‐fabrication stage to deliver properties beyond what is found in naturally occurring materials (e.g., negative incremental stiffness,^[^ ^18^ ^]^ Poisson's ratio,^[^ ^19^ ^]^ compressibility,^[^ ^20^ ^]^ and electromagnetic permittivity^[^ ^21^ ^]^). Shape reconfiguration characteristics of multistable metamaterials offer programming their topology and properties/functionalities in the post‐fabrication stage leading to greater freedom of their adaptability to constantly changing requirements in real‐life applications.^[^ ^15^, ^16^, ^22^, ^23^, ^24^, ^25^, ^26^, ^27^ ^]^ Recently, shape transformation and structural bistability have been utilized to develop mechanical switches, reusable impact shields, soft jumpers, and stretchable sensors.^[^ ^28^, ^29^, ^30^, ^31^, ^32^, ^33^, ^34^ ^]^ A deep understanding of sequential deformations, snapping‐through instability, and snap‐back induced energy release is indispensable to leverage the capabilities of mechanical multistability. Herein, we present a methodology to find the deformation sequences along with the snapping‐through/back behavior in a 1D array of bistable cells (chain). This method determines the governing physical conditions for controlling the number of stable configurations and the amount of snap‐back induced released energy, which requires identifying the continuous force/energy–displacement paths including inaccessible paths. Understanding the shape transformation sequences in reconfigurable metamaterials enables us to: first, design an intelligent mechanical sensor/memory device that restores the deformation history from limited recorded data; second, define arithmetic operations in mechanical multistable materials in order to envision their applications in smart mechanical systems; and, third, design a bidirectional multistable chain to realize tunable chiral materials.^[^ ^35^, ^36^ ^]^ We deem our findings are a step toward the systematic design of multistable chains, which requires a thorough understanding of their force/energy–displacement continuous paths.

Dynamic systems with multiple stable states can generally form memories encoded biochemically in a living system^[^ ^37^ ^]^ or electromagnetically in an engineered one.^[^ ^38^, ^39^ ^]^ The latest technology in memory devices uses charge‐trap‐flash‐memory cells to store data;^[^ ^40^, ^41^, ^42^ ^]^ each cell requiring 2^*n*^ − 1 charge trap levels to offer *n* bits. Hence, increasing their capacity becomes drastically challenging due to the manufacturing limitations. We demonstrate how information can be recorded and encoded in mechanical memories (multistable chain of elastic bits/bistable cells) by mechanical loading and unloading at merely one point on the exterior boundary that is the top of the chain. The developed mechanical memory in this work requires *n* cells to form *n* bits. Understanding the continuous force/energy–displacement paths contributes to the potential realization of a new class of charge‐trap‐flash‐memory that requires *n* charge trap levels for achieving *n* bits, as compared to 2^*n*^ − 1 in conventional memories.

The programmability of snapping sequences in multistable chains can be exploited to tune exotic mechanical properties. This strategy is showcased by tuning the chirality of a bidirectional multistable chain composed of tensegrity‐like transversely bistable unit cells. Controlling deformation sequences in two directions enables transforming a left‐hand chirality into a right‐hand or a neutral one. Consequently, our research paves a cprogramming/tuning the properties of advanced materials in the post‐fabrication stage.

## 2 Result and Discussion

**Figure 1:**
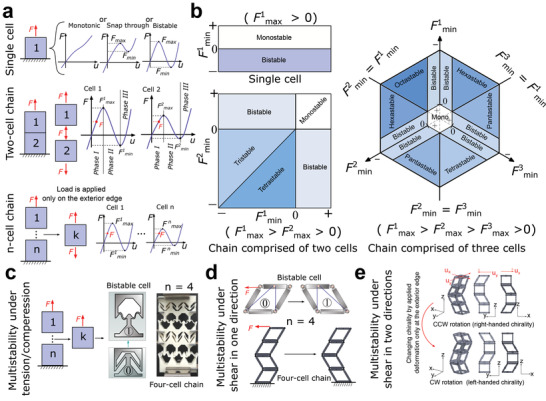
Multistable chain; a) definition of the chain and instability forces. b) The possible number of stable configurations for a single cell and a chain with two and three cells. Multistability under c) tension/compression, d) shear in one direction, and e) shear in two directions.

A chain is created by connecting unit cells in series; each cell or building block can have monotonic or snap‐through behavior. A cell is bistable if the force–displacement curve intersects more than once with the zero load line. Here we realize a multistable chain by connecting a combination of monotonic and bistable elements (**Figure** **1**a).

In the chain definition, we assume the external stimulation, such as displacement, is applied just on the top of the chain, which is the case when excitation is merely available at one boundary. The maximum and minimum instability forces (*F*_max_ and *F*_min_) of each cell determine the deformation sequences and the number of stable configurations of the chain (Figure 1b). A chain comprised of different types of bistable cells can show distinctive functionalities. Herein, two types of chains have been studied. The first type is comprised of inclined beam bistable cells (Figure 1c) in which reconfigurations occur under tension/compression. The other type is composed of newly designed tensegrity‐like bistable cells with reconfigurations under shear loading both in one direction (Figure 1d) and two directions (Figure 1e). In both designs, each cell preserves its properties after connecting to adjacent cells in the chain.

**Figure 2:**
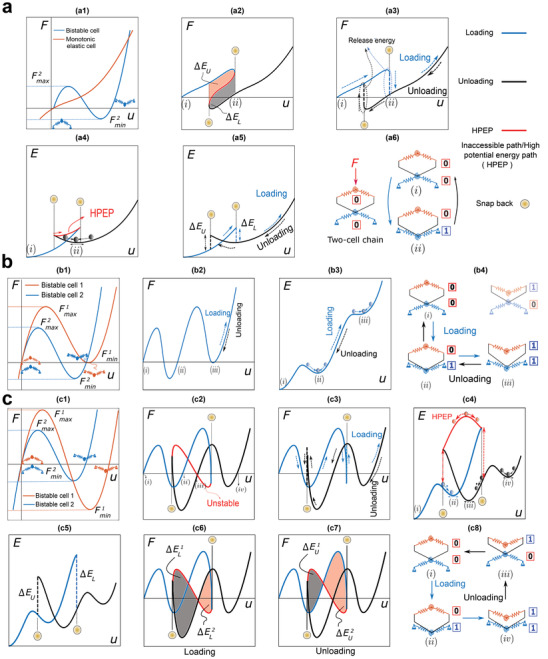
Analysis of a chain with two unit cells: a) soft monotonic and bistable cells; b,c) two bistable cells respectively characterized by $F_{\max}^{2}<F_{\max}^{1}$, $F_{\min}^{2}<F_{\min}^{1}$ and by $F_{\max}^{2}<F_{\max}^{1}$ $F_{\min}^{1}<F_{\min}^{2}$. a1,b1,c1) Force–displacement curves of each cell comprising the chain, a2,b2,c2) continuous force–displacement, a3,b2,c3) displacement‐controlled force–displacement, a4,b3,c4) continuous energy–displacement, and a5,b3,c5) displacement‐controlled energy–displacement paths of the chains (a6,b4,c8) configuration of each cell under loading and unloading. (c6) and (c7) snap‐back‐induced released energy under compression and tension, respectively.

To better understand the behavior of multistable chains under the applied displacement at one end, we first investigate the force/energy–displacement behavior of a two‐cell chain as shown in **Figure** **2**.

Under a displacement‐controlled loading condition a chain releases no energy (Figure 2b2,b3) unless it experiences a sudden drop/jump in the measured reaction force, caused by the instability of the chain's internal architecture. For example, sudden changes in the displacement‐controlled curves found in Figure 2a3,c3 imply instantaneous release of energy (snap‐back‐induced energy release). Existing studies have mainly focused on controlling the snap‐back‐induced released energy based on the stiffness of a monotonic elastic cell in series with a snap‐through cell^[^ ^16^ ^]^ (Figure 2a). However, we present a new systematic methodology for engineering the energy release in chains made of arbitrary mono/bistable cells by tuning their order of instability forces, that is, *F*_max_ and *F*_min_ (Figure 2c). Integrating the area under the apparent force–displacement curve obtained under a displacement‐controlled condition to find the energy–displacement curve is a valid strategy unless there are sudden drops/jumps in the force–displacement curve. In this case, integration over inaccessible paths is required (Figure 2a2,c2). Therefore, we need to adopt a theoretical approach to fully identify force/energy–displacement paths (herein called continuous paths). The continuous path includes high potential energy paths (HPEP) that do not physically occur under a displacement‐controlled condition. To find the continuous paths, we identify the bifurcation points (where force–displacement curve self‐intersects), snap‐through, and snap‐back structural instabilities. To decipher the path, we assume that: 1) the underlying architecture of each cell of chain deforms continuously; 2) force equilibrium between all cells is preserved; and 3) the force and energy–displacement curves are continuous at the cell level. Based on these conditions, elastic energy is preserved in the continuous path and is not dissipated. Although the displacement–control condition suffices to capture the continuous path in the snap‐through instability, additional constraints should be imposed to realize the snap‐back path. Arch length method^[^ ^44^ ^]^ mathematically captures snap‐back by imposing a coupling between the force and displacement, in which the displacement changes while the force equilibrium is preserved. Our method is based on the physical definition of instabilities. In this methodology, snapping of cells happen in a sequence based on their instability forces. This extra information simplifies the solution and prevents the solver from diverging.

To understand the snapping sequence, force–displacement behavior of each cell is divided into three phases with the boundaries characterized by the maximum and minimum instability forces (Figure 1a). Phases I and III are stable with positive stiffness while phase II is instable with negative stiffness. Finding the phase transition in each cell of the chain is a challenge (Figure S18, Supporting Information). To overcome this challenge, we consider two factors: 1) cell transformation direction and 2) determinant cell index. Cell transformation direction is obtained for each cell based on the force equilibrium in the chain and demonstrates current compression or expansion of the cell. The determinant cell index distinguishes the cell undergoing a phase transformation. In this procedure (Section S1.1, Supporting Information), the displacement is applied to the determinant cell and the corresponding force is obtained. In a chain, cells are in series and, therefore, they are under an identical force. Consequently, the displacements of the remaining cells are obtained by exploiting the force equilibrium. It should be noted that cells undergo phase transformation one at a time.

In this study, the developed algorithm attains the continuous path of multistable materials by considering that the constitutive cells are decoupled, that is, the deformation of one cell does not affect the behavior of other cells in the chain. This is not a valid assumption in some multistable materials, for example, perforated shellulars, in which the coupling of deformation in neighboring cells should also be considered (Section S3.2, Supporting Information).

Here, a summary of governing equations of the chain is provided. The elastic energy of the chain is determined by:

(1) $$U_{T}=\sum_{i=1}^{n}\int_{0}^{u_{i}}f^{i}\left(u_{i}\right)\cdot du_{i}$$

where *u_i_*, *f^i^*, *n* and *U*_T_ are the displacement of *i*th unit cell, internal force of *i*th unit cell, number of cells assembled in series in a chain and total energy, respectively. Compatibility of the chain implies:

(2) $$u_{T}=\sum_{i=1}^{n}u_{i}$$

where *u_T_* is the total displacement of the chain. Equilibrium in the chain is written as:

(3) $$F_{T}=F_{1}=\cdot \cdot \cdot F_{i}=\cdot \cdot \cdot F_{n}$$

(4) $$\left[\begin{array}{c} \begin{array}{c} F_{1}=f^{1}\left(u_{1}\right)\\ \vdots \\ F_{i}=f^{i}\left(u_{i}\right) \end{array}\\ \vdots \\ F_{n}=f^{n}\left(u_{n}\right) \end{array}\right]$$

where *F_i_* and *F_T_* are the *i^th^* unit cell internal force and external force of the chain, respectively. This set of nonlinear equations can have multiple answers for a specifically given displacement. Therefore, the aforementioned algorithm is needed to obtain deformations continuously. The solution algorithm is presented below:

A snap‐through behavior is represented mathematically as:

(5) $$\frac{df^{i}\left(u_{i}\right)}{du_{i}}=0$$

where Equation (5) must have two answers for a non‐monotonic cell ($\left\{\left[u_{i}^{I},\;F_{i}^{I}\right],\;\left[u_{i}^{II},F_{i}^{II}\right]\right\}$), corresponding to the boundaries of the three phases. Initially, all cells are in their phase I, Δ*u_i_* > 0 and the first cell that reaches the maximum instability force $F_{i}^{I}$ (force at the boundary of phases I and II) is the determinant cell. The following conditional statement defines the first cell that reaches phase transition, $if\;F_{\xi }^{I}=Min\;\left\{F_{1}^{I},\cdot \cdot \cdot F_{i}^{I},\cdot \cdot \cdot F_{n}^{I}\right\}\to$ determinant cell index = *ξ*.

Here, we define *λ*_*ξ*_ as the index direction of the determinant cell; initially, *λ*_*ξ*_ > 0, and it indicates a forward deformation toward cell transformation. Small *ε* > 0 is added to the deformation of the determinant cell, and the corresponding force *f* ^*ξ*^(*u*_*ξ*_) in the cell is obtained. Since the cells are in series, the displacement of all the other cells *u_i_* (and consequently *δu_i_*) are obtained in their current phase using *F_T_* = *f* ^*ξ*^(*u*_*ξ*_). From this point forward, whenever *F_T_* reaches $F_{i}^{I}$ or $F_{i}^{II}$, determinant cell index *ξ* changes to *i*. Small *δu*_*ξ*_ = *ελ*_*ξ*_, in which the index direction $\lambda_{\xi }=\frac{\delta u_{i}}{|\delta u_{i}|}$, is added to the deformation of the new determinant cell, and the process is continued until all the cells are in their third phase (Section S1.1, Supporting Information).

To determine the stable states of the chain on the continuous path, the following two conditions should be satisfied for the whole chain and the individual cells, respectively:

(6) $$\frac{dU_{T}}{du_{T}}=0\;\mathrm{and}\;\frac{d^{2}U_{T}}{du_{T}^{2}}>0$$

and

(7) $$\forall i\to \frac{dF_{i}}{du_{i}}>0$$

**Figure 3:**
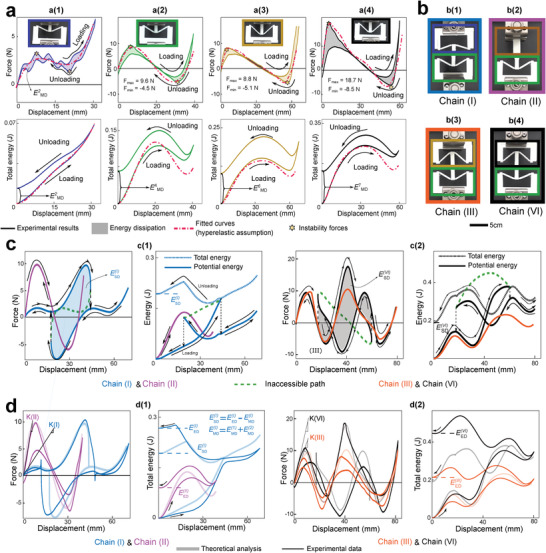
Experimental evaluation of a 1D chain of two unit‐cells and comparison with theoretical analysis: a) Force/total energy–displacement curves for a1) Cell#2, a2) Cell#4 (reference unit‐cell), a3) Cell#6 and a4) Cell#7; b) Two‐cell chains formed by b1) Cell#2 (soft) and Cell#4 (reference bistable) showing two stable configurations and, b2) Cell#1 (rigid) and Cell#4 (reference bistable) showing two stable configurations, b3) Cell#6 and #4 (two bistable cells) with one discrepancy (order of maximum instability forces of cells does not match the order of minimum instability forces) revealing three stable configurations and b4) Cell#7 and #4 (two bistable cells) with ideal combination (order of maximum instability forces of cells match the order of minimum instability forces) indicating four stable configurations. c) Theoretical analysis of force–displacement and total/potential energy–displacement of the chains shown in (b). d) The experimental results and comparison with the theoretical predictions for the chains shown in (b).^[^ ^16^, ^17^ ^]^

By integrating the area under the continuous path while the chain is being deformed in a displacement‐control manner, the released energy from snap‐back can be calculated regardless of the starting point of loading. Indeed, Δ*E_L_* and Δ*E_U_*, as instantaneous snap‐back induced released energy in a chain during loading and unloading, can be tuned independently by instability forces $F_{\min}=F_{i}^{II}$ and $F_{\max}=F_{i}^{I}$ of cells forming the chain, respectively. Moreover, the snapping sequence of the chain conforms to the ascending order of *F*_max_ in loading and descending order of *F*_min_ in unloading. Having two bistable cells with $F_{\max}^{1}<F_{\max}^{2}$ in series, $F_{\min}^{1}<F_{\min}^{2}$ and $F_{\min}^{1}>F_{\min}^{2}$result in three (Figure 2b4) and four (Figure 2c8) stable states, respectively.^[^ ^16^, ^17^ ^]^ The theory is evaluated by a set of experiments on a chain with two unit cells (**Figure** **3**).

To experimentally evaluate the programming of the energy dissipation by instability forces and compare it with the existing approaches (increasing snap‐back induced energy release by decreasing stiffness of snapping parts^[^ ^16^, ^17^ ^]^), a series of experimental tests has been designed (Figure 3). We 3D print one solid column (cell#1) and six alternative cells (cell#2 to #7) with different hinged inclined beams to achieve dissimilar instability forces and stiffnesses. Each sample is tested to obtain its instability forces, and more importantly the material's viscoelastic energy dissipation in one cycle of load (tension) and unload (compression). Cell#4 (reference cell) is selected as the underneath cell in all two‐cell chains (Figure 3b). We estimate the snap‐back induced energy release using the theoretical model adopting the aforementioned algorithm. In this model, we assume the base material has hyperelastic behavior and neglect the material energy dissipation. Force–displacement behavior of each cell is obtained by fitting a polynomial function to the average of loading and unloading force–displacement curves. From the energy perspective, neglecting viscoelastic energy dissipation causes the theoretical model to underestimate the total released energy compared to the experimental data.

The theoretical approach along with the solution algorithm can model the nonlinear reconfiguration of all chains made by any combination of cells in series (Section 1, Supporting Information). However, in Figure 3, we mainly study the combinations of the reference cell (cell#4) with the soft monostable (cell#2), rigid (cell#1), soft bistable (cell#6), and stiff bistable (cell#7). Chains (I) and (II) (Figure 3b1,b2) demonstrate the idea of increasing the energy release by decreasing the overall stiffness (*K*) of the chain, while chains (III) and (VI) indicate the idea of simultaneous increase in the energy release and the overall stiffness of the chain (other two chains, that is, chains (IV) and (V), have also designed to show the intermediate stages of the increase in energy release from chain (III) to chain (VI). The details about their constitutive cells together with the corresponding force/energy–displacement curves are provided in Section S1.8, Supporting Information). By comparing chains (I) and (II), it can be concluded that decreasing the stiffness of the upper cell leads to more energy release. In the chain design, the instability forces of the reference cell (cell #4) govern the instability behavior of the chain. Between cells #1 and #2, cell#2 has lower stiffness and hence deforms more under the same load, which means it stores more energy when the reference cell reaches its instability point. When cell #4 is in unstable mode, the total force in the chain reduces. Accordingly, in chains (I) and (II), cells #2 and #1 deform reversely to follow force reduction. Under the displacement‐controlled condition, force and deformation direction are positive at the instability point, which implies that the chain should have positive energy changes. In the chain (II), cell #4 and cell#1 deforms in the forward and backward directions after instability with positive and negative energy changes, respectively. Due to the small stored energy in cell#1, total energy changes are still positive. However, in the chain (I), stored energy in cell#2 is significantly higher, leading to a negative change in the total energy, which appears as a sharp drop in the force–displacement curve (Figure 3c1) (Section S1.3, Supporting Information).

Our study on the continuous path enables the calculation of potential energy of the chain and unfolds inaccessible regions of the corresponding force–displacement curve (Figure 3c, green dashed line). Due to the non‐viscoelastic base material assumption in the theoretical investigation, potential energy returns to zero after completing a loading and unloading cycles; however, the total energy in the displacement‐controlled loading and unloading does not return to the same point, due to the snap‐back‐induced released energy. Changing instability forces order between two cells in the chain create two possibilities. The first possibility (chain (III)) is obtained by $F_{\max}^{2}>F_{\max}^{1}$ and $F_{\min}^{2}>F_{\min}^{1}$ and shows three stable configurations and no energy release in loading and unloading. The second possibility (chain (VI)) is obtained by $F_{\max}^{2}>F_{\max}^{1}$ and $F_{\min}^{2}<F_{\min}^{1}$ demonstrating four stable configurations and a significant amount of energy released in loading and unloading. In chain (VI), different configurations at the same strain imply different potential energy landscapes in which the drops are associated with the release of energy at snap‐back points (Figure 3c2). $E_{U}^{-s}-E_{U}^{+s}$ Indicates the difference of potential energy before and after a snap‐back point. Interestingly, when the orders of instability forces of two cells in a chain are $F_{\max}^{2}>F_{\max}^{1}$ and $F_{\min}^{2}<F_{\min}^{1}$ (chain (VI)), shape reconfigurations in loading and unloading are different (black line in Figure 3c2); however, when $F_{\max}^{2}>F_{\max}^{1}$ and $F_{\min}^{2}>F_{\min}^{1}$ (chain (III)), the configurations during the loading and unloading are the same with no release of energy (orange line in Figure 3c2). Our goal is to increase the snap‐back‐induced released/dissipated energy, that is, structural energy dissipation (*E*_SD_), in one cycle of loading and unloading.

**Figure 4:**
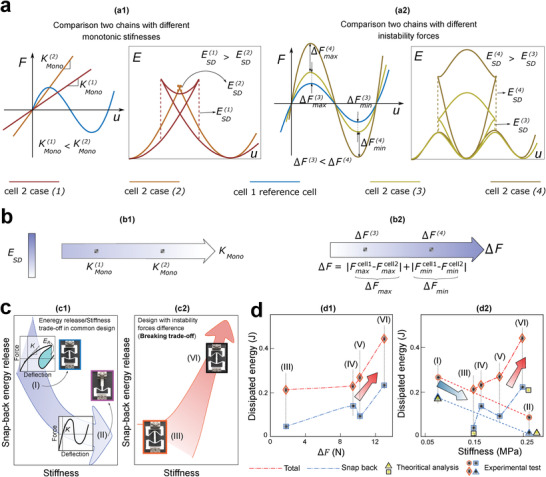
Tuning released/dissipated energy with two strategies. a) Comparing energy releases with changing a1) stiffness and a2) instability forces. b) The factor playing the role in the energy release in each strategy: b1) changing stiffness and b2) differentiating instability forces. c) Snap‐back energy release/stiffness trade‐off in the first strategy (c1) and breaking this trade‐off with instability forces (c2). d) Results of two chains (I and II) for investigating the first strategy and four chains (III–VI) for studying the second strategy (breaking the trade‐off).

As previously mentioned, by decreasing the stiffness of a cell in series with a bistable cell, the snap‐back‐induced energy release can be increased (**Figure** **4**a1). This is the conventional strategy of enhancing energy release during snap‐back that has been adopted in the literature.^[^ ^16^, ^17^ ^]^ This approach, however, results in a reduced total stiffness of the chain, which might be considered inappropriate when more stiff designs are required (conventional trade‐off between the overall stiffness and the energy release). Our theoretical results reveal that increasing the differences in the instability forces (i.e., $\Delta F=|F_{\max}^{2}-F_{\max}^{1}\left|+\right|F_{\min}^{2}-F_{\min}^{1}|$ in case of a two‐cell chain) can be considered an alternative strategy to enhance the snap‐back energy release without sacrificing the overall stiffness of the chain (Figure 4a2). Accordingly, one can increase the snap‐back‐induced energy release and the stiffness of the chain simultaneously (Figure 4b) and break the aforementioned trade‐off (Figure 4c). More details on the stable configurations of a two‐cell chain and the corresponding released energy are provided in Sections S1.3 and S1.4, Supporting Information. To validate the theoretical results experimentally, various combinations of the reference bistable cell with six other cells, a solid column, and five cells with different inclined beams are tested in one loading and unloading cycle (Section S1.8, Supporting Information). The results of chains (I) and (II) in Figure 3c confirm that by decreasing the stiffness of the soft monostable cells, which reduces the overall stiffness of the chain, energy dissipation can be increased (the first strategy). Further experiments on four combinations of the cells (chains (III–VI)) demonstrate a significant increase in energy dissipation by increasing both instability forces and stiffness (second strategy). These studies confirm that energy dissipation can be rationally programmed by incorporating a combination of stiffness tuning and instability force differences in a snapping chain.

The energy dissipation (*E*_ED_) of a two‐cell chain during one cycle of loading and unloading in the experiment is the sum of the *E*_SD_ and other energy losses herein referred to as material energy dissipations (*E*_MD_) (Figure 4d1,d2 blue dash line). As shown in Figure 4d2, the theoretically calculated *E*_SD_ using the discussed continuous path closely agrees with those from the experiments, further supporting the presented methodology. To obtain the snap‐back‐induced energy dissipation experimentally (*E*_SD_), we subtract the material energy dissipation associated with the constitutive cells (*E*_MD_) demonstrated in Figure 3a from the chain total energy dissipation (*E*_ED_) presented in Figure 3d.

**Figure 5:**
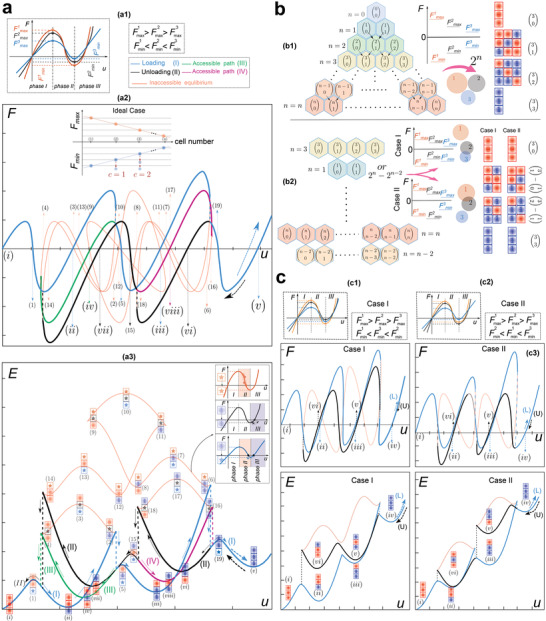
Mathematical description for the number of stable configurations. a) A chain comprised of three bistable unit cells, characterized by a1) $F_{\max}^{1}>F_{\max}^{2}>F_{\max}^{3}\;\mathrm{and}\;F_{\min}^{1}<F_{\min}^{2}<F_{\min}^{3}$ (ideal case with eight stable configurations), and its corresponding a2) force–displacement and a3) potential energy–displacement curves. b) Mathematical model based on Pascal's triangle along with the set theory to capture the number of stable configurations: b1) Ideal case (without a discrepancy), b2) one discrepancy, which has been divided into the case I with $F_{\max}^{1}>F_{\max}^{2}>F_{\max}^{3}\;and\;F_{\min}^{1}<F_{\min}^{3}<F_{\min}^{2}$, and case II with $F_{\max}^{1}>F_{\max}^{2}>F_{\max}^{3}\;and\;F_{\min}^{2}<F_{\min}^{1}<F_{\min}^{3}$. c) Cases I and II with six stable configurations: c1,c2) force–displacement and energy–displacement curves.

**Figure** **5** corroborates the versatility of the developed methodology for finding the continuous path and, more importantly, it illustrates how to capture the stable configurations in a chain mathematically. As shown in Figure 5a3, the continuous path demonstrates that during the snap‐back instability if multiple energy paths exist below the current energy landscape, the energy level drops to the nearest energy path, leading to the least released energy (more details in Section S2, Supporting Information). To yield robust and programmable structures, we show how the arrangement of the minimum and maximum instability forces of the cells solely determines the number of stable configurations. The order of *F*_max_ and *F*_min_ in Figure 5b1 is defined as an ideal arrangement for which the corresponding *n*‐cell chain can transform into all 2^*n*^ possible stable configurations. In Figure 5b1, Pascal's triangle diagram mathematically expresses the number of stable configurations as a selection of different sets of binary cells.

**Figure 6:**
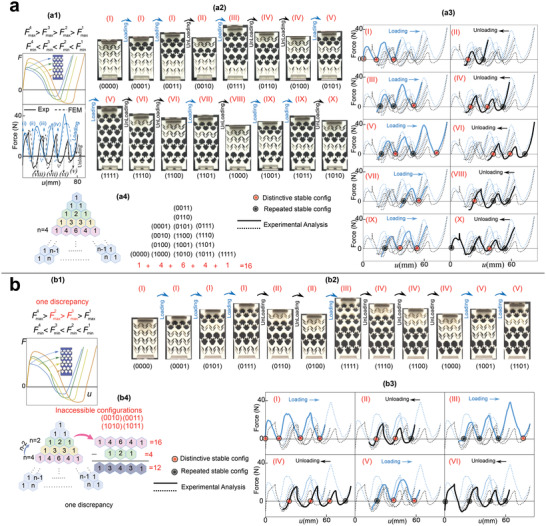
Experimental validation of accessible configurations in a chain comprised of four bistable cells. a,b) Multistable metastructure with zero and one discrepancy: a1,b1) order of instability forces, and the force–displacement curve of each constitutive cell; a2,b2) sequences of loading and unloading for achieving all possible chain configurations; a3,b3) corresponding force–displacement curves associated with the represented configurations in the (a2,b2), (a4,b4) corresponding Pascal's triangle for the achievable configurations.

As shown in Figure 5b2, applying a single discrepancy to the *F*_min_ sequences of the ideal case (defined as changing one of the $F_{\min}^{i+1}<F_{\min}^{i}$ cases to $F_{\min}^{i+1}<F_{\min}^{i}$) creates two sets in series, a smaller ideal set with *n* − 2 binary cells and a tristable cell (later discussed in **Figure** **6**b), presenting a total of 3 × 2^*n* − 2^ stable configurations. The minimum number of accessible stable configurations for an arbitrary number of discrepancies can be obtained by the following closed‐form expressions:

(8) $$\psi \left(n,\;c,m\right)=\sum_{j=0}^{c}{\left(-1\right)}^{j}\left(\begin{array}{c} c\\ j \end{array}\right)\left(\begin{array}{c} n-2j\\ m-j \end{array}\right)\;\;n-2c\geq 0$$

(9) $$\omega \left(n,c\right)=\sum_{j=0}^{c}{\left(-1\right)}^{j}\left(\begin{array}{c} c\\ j \end{array}\right)2^{n-2j}\;\;n-2c\geq 0$$

where *c* is the number of discrepancies, $\left(\begin{array}{c} c\\ j \end{array}\right)$ is the number of combinations of *c* items taken *j* at a time, *ψ*(*n*, *c*, *m*) is the minimum number of stable configurations that have *m* cells (*m* = 0, …, *n*) in the second stable state, and *ω*(*n*, *c*) is the minimum total number of stable configurations. As presented in Figure 5b2, for two chains having different single discrepancies, investigating all stable states using set theory (based on *F*_min_ and *F*_max_ sequences) not only provides *ψ* and *ω*, but also reveals the inaccessible binary states. Furthermore, the alignment between these findings and the respective outcomes of the continuous paths (Figure 5c) verifies the capability of our derived mathematical approach to capture distinct stable configurations without investigating the force/energy–displacement curves of the chain (Section S2, Supporting Information).

**Figure 7:**
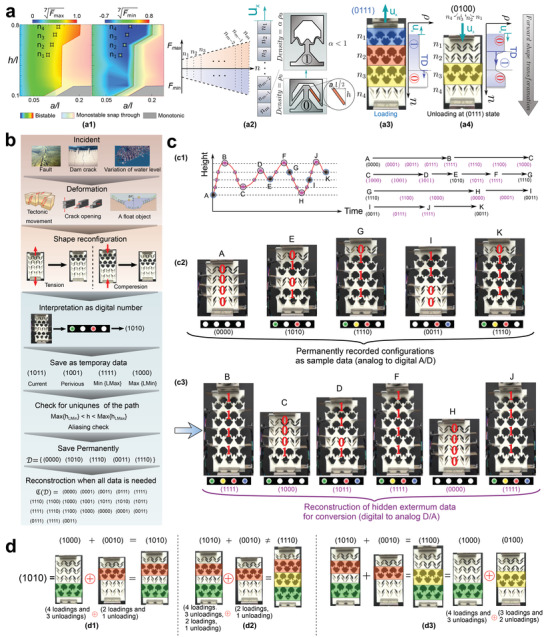
Designing an ideal chain for a mechanical sensor and a memory device. a) Wave propagation in a multistable chain comprised of four independent rows of bistable cells made of inclined beams: a1) numerically obtained a correlation of *F*_max_ and *F*_min_ with the inclined beam's geometrical parameters; the results are represented as the seventh root of actual results that are normalized by the maximum value of *F*_max_. a2) Definition of the unit cell's density in the initial and second stable configurations and an ideal chain comprised of *m* cells. a3) Shape transformation occurs solely in the opposite direction of the mechanical stimulus, characterized by a forward propagating wavefront of density along the chain. a4) Unloading the chain before reaching the fully stretched state (1111) still results in a forward shape transformation, leading to the appearance of the second wavefront. b) Proposed sample application in monitoring of an incident deformation (light purple), consisting of sensing (light brown), recording, and reconstructing data (light blue); c) Mechanical sensor/memory devices: c1) time evolution of a monitored system's height under an arbitrary deformation scenario. c2) Permanently recorded data. c3) Restored latent data from the permanent data of the mechanical sensor utilizing the reconstruction function. d) Mechanical and binary summations and their relationship: d1) equivalence of mechanical summation (⊕) and binary summation ( + ) when none of the *i*th digit of the two binary addends are simultaneously unity and the left addend is larger, d2) inequivalent mechanical and binary summation when second digits of the two addends are simultaneously one, and d3) analogy between the mechanical and binary summations.

Similar to encoding digital data to bistable units, a mechanical bistable cell as a bit of nonvolatile memory device retains the stored data when an external stimulus is removed.^[^ ^45^ ^]^ Recently, a mechanical memory has been developed by means of parallel arrays of bistable cells, in which read/write head should move over each cell to read/write data.^[^ ^32^ ^]^ We introduce an engineered chain of bistable cells connected in series to develop a high‐capacity memory device. Saving *n* bits of data in a chain in binary form requires 2^*n*^ stable states. We demonstrate that the ideal chain creates *n* bits with *n* bistable cells, while a full discrepant arrangement (i.e., $F_{\min}^{n}<\cdot \cdot \cdot <F_{\min}^{1}$), containing *n*(*n* − 1)/2 discrepancies, requires 2^*n*^ − 1 cells to offer the same capacity. The shortest path between two stable states in the chain indicates writing speed in a mechanical memory device. In the ideal arrangement, the shortest path between two arbitrary stable states without crossing the continuous path repeatedly involves at most *n*(*n* + 1)/2 reconfigurations (Section S2, Supporting Information). For obtaining the shortest path between any arbitrary two stable states in ideal arrangement, a reconstruction function is developed in the mechanical sensor (later discussed in **Figure** **7**).

To evaluate the validity of the derived theory and the effect of discrepancies on the achievable configurations, two snapping chains have been designed and 3D printed (Figure 6). The first chain has no discrepancy and brings eight distinct configurations in tension till fully extended and compression till fully compacted (Figure 6a1). In addition to these eight configurations, eight additional stable configurations exist which are attainable only if certain sets of loading and unloading sequences are applied. These sets guide the chain to maneuver through all the possible but latent deformation paths. The procedure to trace these latent paths and the associated configurations are explored using the shortest paths discussed later (Figure 7). As shown in Figure 6a2,a3 five sets of loading together with five sets of unloading can cover all achievable configurations of the chain (Figure 6a3). Each row of Pascal's triangle diagram in Figure 6a4 consists of an assortment of units representing the number of chain configurations with the same number of cells in their second state, regardless of the order of cell states. For instance, the row marked by *n* = 4 consists of five units that represent: a) no cell in the second state (one possibility); b) one cell in the second state (four possibilities); c) two cells in the second state (six possibilities); d) three cells in the second state (four possibilities); and e) four cells in the second state (one possibility). Inducing a discrepancy in the design of the chain's second cell by increasing the thickness of the beams, changes the order of maximum instability force between cells 2 and 3; consequently, the new chain demonstrates 12 distinct configurations as theoretically predicted by Equations (8 and 9). As demonstrated in Figure 6b2,b3, these configurations are achievable by three loading sets and two unloading sets (Figure 6b3).

The effect of geometrical parameters on maximum and minimum instability forces is studied in Figure 7a1. There are three design parameters: thickness‐to‐width ratio (*a*/*l*), height‐to‐width ratio (*h/l*), and inclined beams boundary conditions. In addition, an inclined beam can experience three major beam deformation mechanisms: bending, axial deformation, and buckling.

Increasing the *h/l* ratio while the *a*/*l* ratio is kept constant, increases both cell stiffness and slenderness ratio of the inclined beams (defined as the beam length divided by its thickness). As such, at smaller *h/l*, with more bending and less axial deformations in the beams, the magnitude of instability forces increases, while at larger *h/l*, slender beams undergo buckling resulting in a smaller absolute value of instability forces. On the other hand, keeping *h/l* constant and increasing *a*/*l* result in stiffer beams in bending and axial deformations, and reduce the possibility of buckling, all of which contribute to a larger *F*_max_. By increasing *a*/*l* ratio, the value of *F*_min_ of slender beams decreases. However, by moving toward larger *a*/*l* ratios, the value of *F*_min_ increases, and it eventually becomes positive (snap‐through, Figure 7a1 grey part). The overall effects of thickness‐ and height‐to‐width ratios on the maximum and minimum instability forces, determined by nonlinear finite element analysis, are shown in Figure 7a1. Based on the instability forces, an ideal four‐cell chain has been designed (Figure 7a3,a4). The sequence of reconfigurations provides an insight into wave‐like behavior, which is defined as the propagation of shape transformation due to structural instabilities in the chain under an externally prescribed displacement. As illustrated in Figure 7a3,a4, the direction of propagating shape transformation of an ideal chain (designed to follow the *F*_max_ and *F*_min_ order presented in Figure 7a2) remains forward, regardless of the initial state of the chain or the external stimulus direction. Interestingly, although the transformation direction remains unchanged, the chain shows two different wave fronts, one activated in loading (Figure 7a3) and the other one in unloading (Figure 7a4) (Section S3, Supporting Information). This idea may also be pertinent to the observed forward‐only movement of food in the intestine,^[^ ^13^ ^]^ both in the contraction and relaxation of its muscles).

Exploiting structural instability in multipurpose applications is a recent endeavor;^[^ ^9^, ^10^, ^15^, ^16^, ^18^, ^46^, ^47^, ^48^ ^]^ however, its utilization in electro‐mechanical sensing devices has hardly been investigated. Numerous incidents are translated into deformation/displacement (e.g., fault in earth's crust, crack in dams, and water level variation) and tracking these incidents with minimum stored data can be an advantage (Figure 7b). Herein, as presented in Figure 7c, we fabricate a mechanical sensor based on an ideal chain to continuously capture displacement variations in mechanical devices. The exterior boundary of this sensor is connected to the monitored target system, making it possible to persistently track the overall displacement. Moreover, the non‐reciprocity of the stable configurations during loading and unloading allows the device to record the maximum number of recent deformation variations without any external source of energy such as electricity. This mechanical sensor/memory not only holds the above‐mentioned traits but also allows the reconstruction of all data, including extremum values (points where the displacement direction is changing), from a limited number of permanently recorded data. For instance, it is possible to reconstruct three extremums (B,C, and D) of the applied displacement merely from Point A and E (Figure 7c1). Based on the number of bistable cells (*n*) and the applied load path, this mechanical sensor can restore up to *n* − 1 extremum points between two sample data as shown in Figure 7c. This feature extricates sensor from saving all the history. This exciting property arises from the fact that in the ideal chain, forward displacements make different configurations compared to backward displacement (A to B compared with B to C in Figure 7c1).

Nevertheless, a question emerges: how can we reconstruct an approximate analog (continuous) path from the limited digital (discrete) stored data? The main challenge for this data reconstruction is the multiple paths between two recorded digital data. The necessary and sufficient condition as a sampling theorem is defined here to guarantee the uniqueness of the path and to prevent aliasing:

(10) $$\begin{array}{ccc} & & Max\left\{h_{\mathrm{local}\;\min\left(1\right)},\;h_{\mathrm{local}\;\min\left(2\right)},\cdot \cdot \cdot \right\}<h_{t}<\\ & & Min\left\{h_{\mathrm{local}\;\max\left(1\right)},\;h_{\mathrm{local}\;\max\left(2\right)},\;\cdot \cdot \cdot \right\} \end{array}$$

where, *i* is the number of times data is permanently stored, *t* > *t* ^(*i*)^, and *h_t_* is the height of the chain at time *t* right after *t* ^(*i*)^, and

(11) $$\;h^{\left(i\right)}=\left\{\begin{array}{cc} h_{\mathrm{local}\;\min\left(1\right)} & h_{t}>h^{\left(i\right)}\\ h_{\mathrm{local}\;\max\left(1\right)} & h_{t}<\;h^{\left(i\right)} \end{array}\right.$$

in which, *h* ^(*i*)^ (*i* = 1, 2, …) or height at *t* ^(*i*)^, is an extremum point that determines whether the recorded data is a local minimum or maximum. By applying Equation (10), the shortest path is equivalent to the chain's actual path. After a permanently stored data (e.g., point A), each time loading direction is changed, that point is stored temporarily as a local minimum or maximum state (e.g., point B). The maximum height of the local minimum and the minimum of local maximums are crucial bonds that determine the upper and lower bonds. If displacement from the latest permanently saved data passes or reaches these bonds as expressed in Equation (10), the data reconstruction function cannot restore the history of applied displacement. Hence, whenever the condition in Equation (10) is not satisfied (like 1011 after point E), the previous configuration (e.g., point E) is saved permanently. The sampling theorem is utilized to keep the maximum possible history of reconfigurations (information) between the two recorded data, significantly obviating the need to store all the data in a full displacement of a target system. Each permanent data point (*N* ^(*i* + 1)^), which contains the *n* binary (0 and 1) representation of the stable states of the cells in the chain is saved in D as:

(12) $$D=\left\{N^{\left(1\right)},\cdot \cdot \cdot ,N^{\left(i\right)},N^{\left(i+1\right)},\cdot \cdot \cdot \right\},\;\;\;N^{\left(j\right)}={\left(\bar{a_{n}\cdot \cdot \cdot a_{1}}\right)}^{\left(j\right)}$$

in which, *a_n_* is the stable configuration of the *n*th bistable cell.

By implementing the following reconstruction function along with Equations (10) and (11) the approximate analog path is recovered:

(13) $$\begin{array}{c} C\left(N^{\left(i\right)},N^{\left(i+1\right)}\right)=\{N^{\left(i,1,1\right)},\cdot \cdot \cdot ,\;N^{\left(i,1,r\right)},\;\cdot \cdot \cdot ,\;N^{\left(i,2,1\right)},\;\cdot \cdot \cdot ,N^{\left(i,k,r\right)},\cdot \cdot \cdot \end{array}$$

where C and *N* ^(*i*,*k*, *r*)^ are the reconstruction function and the restored data between *N* ^(*i*)^ and *N* ^(*i* + 1)^, respectively. In Equation (13), *k* represents the counter for the extremum between time *i* and *i* + 1 (equivalent to the number of changes in the displacement direction added by one). In the reconstruction function C, variable *r* is between 1 and *L* ^(*k*)^, in which *L* ^(*k*)^ is the number of shape transformations in cells occurring between the extremums (for following reconstruction procedure, see Section S6, Supporting Information) and is defined as:

(14) $$\begin{array}{c} \begin{array}{c} L^{\left(k=1\right)}=\left(a_{m}^{\left(i+1\right)}-a_{m}^{\left(i\right)}\right)\left(m\right)+\frac{1-\left(a_{m}^{\left(i+1\right)}-a_{m}^{\left(i\right)}\right)}{2}\left[m-1\right]-\sum_{j=1}^{m-1}a_{j}^{\left(i\right)}\left(\mathrm{For}\;k=1\right) \end{array} \end{array}$$

where *m* is the highest binary place that is different between permanently stored points *N* ^(*i*)^ and *N* ^(*i* + 1)^. The current reconstructed point is called N*^(*i*,*k*, *r*)^. All binary places greater than *m* of *N* ^(*i* + 1)^ and *N* ^(*i*)^ are equal, hence $a_{j>m}^{\left(i\right)}$ of the current, reconstructed point would be equal to those of *N* ^(*i* + 1)^. Then, $a_{j\leq m}^{\left(i\right)}$ as the digits of $N^{*(i,k,r)}=\left(\bar{\;a_{n}^{\left(i\right)}\cdot \cdot \cdot \overset{\leftarrow }{a_{j}^{\left(i\right)}}\cdot \cdot \cdot a_{2}^{\left(i\right)}\;a_{1}^{\left(i\right)}}\right)$ are sequentially obtained from *j* = 1 using:

(15) $$a_{j}^{\left(i\right)}=\left\{\begin{array}{cc} 1 & L^{\left(k=1\right)}>0,\;\;\mathrm{and}\;\;a_{j}^{\left(i\right)}=0\\ 0 & L^{\left(k=1\right)}<0,\;\;\mathrm{and}\;\;a_{j}^{\left(i\right)}=1 \end{array}\right.,\;\;\;\left(j\leq m,\;r\leq \;L^{\left(k=1\right)}\right)$$

For *k* > 1,

(16) $$L^{\left(k>1\right)}=\left(a_{m-\left(k-1\right)}^{\left(i+1\right)}-a_{m-\left(k-2\right)}^{\left(i+1\right)}\right)\left[m-\left(k-1\right)\right]$$

and

(17) $$a_{j}^{\left(i\right)}=\left\{\begin{array}{cc} 1 & L^{\left(k\right)}>0\\ 0 & L^{\left(k\right)}<0 \end{array}\right.,\;\;\left(j<m-\left(k-1\right),\;r\leq \;L^{\left(k\right)}\right)$$

When *j* ≥ *m* − (*k* − 1) the reconstruction function reaches the extremum and the *k* counter should be increased by one. The reconstruction function finds the shortest path between two stable configurations by firstly identifying the leftmost dissimilar digits associated with the highest instability forces and secondly applying the necessary loadings/unloadings to match those digits. This process is repeated unless all the digits are matched from time *i* to time *i* + 1.

The introduced data compression/reconstruction logic can potentially be adapted to efficiently store and reconstruct other time‐dependent or sequential data.

The idea behind the introduced sensor/memory based on the mechanically multistable chain can be regarded as an early step toward mechanical–digital computation. Encoding a chain with a set of loadings and unloadings is the key point of programming and controlling a chain. The presented reconstruction function can provide the smallest sets of shape transformations (i.e., shortest path) to reach any desired binary value from an initial arbitrary state (see Movie S3, Supporting Information). This capability can make the encoding process extremely fast since the chain does not need to go to the zero state to be encoded. Additionally, each stable state has specific mechanical, thermal or acoustic properties,^[^ ^49^ ^]^ which can be programmed to achieve desired properties. To this end, mathematical and logical operations should be developed based on the associated shape transformations. As an example, the connection of the developed mechanical sensor/memory to binary systems is depicted in Figure 7d to distinguish between the mechanical and binary summation operations. In Figure 7d1, we show that 1000 state can be obtained with a set of four loadings (0000 → 1111) and three unloadings (1111 → 1000), and 0010 state can be obtained with set of two loadings and one unloading. Performing these sets of loading and unloading together is equivalent to a mathematical binary summation (four loadings+three unloadings+two loadings+one unloading = configuration of 1010). However, the mechanical summation is not commutative; in other words, a different order of loadings and unloadings in the mechanical summation (e.g., 0010+1000 instead of 1000+0010) results in a different outcome. Furthermore, if two addends have the same digit equal to one (e.g., the second digits of both 1010 and 0010 are equal to one), the result does not agree with the math, which requires further studies.

**Figure 8:**
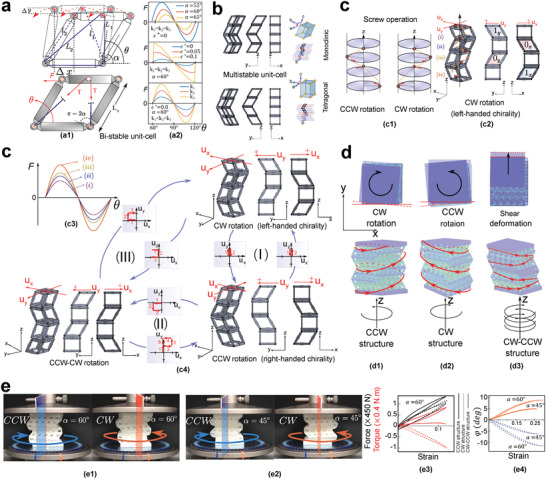
Tunable and programmable chiral materials. a) Ideal bistable cell under a transverse load: a1) nomenclature of the unit cell, and a2) effects of initial locking angle *α*, pre‐strain ε*, and string's stiffness *k* on the force–displacement curve. b) Converting the crystal‐like structure of the multistable chain from monoclinic to tetragonal (along *y*‐direction) by transforming the stable configurations of its cells. c) Convertibility of the intrinsic chiral property of the bidirectional multistable chain: c1) definition of screw operation, c2) definition of the initial and second stable configurations in *x* and *y* directions of a CW chain, c3) force–displacement curves of each cell of a representative designed chain, c4) converting the CCW, CW and CCW‐CW hybrid chiral materials to each other by applying (I), (II) and (III) deformation operations along the continuous path of deformation. d) Finite element simulation results for twist angle under compression; d1,d2,d3) CCW, CW, and directional shear deformation; constant stress contours (e.g., red lines) match with the chirality of the structure. e) Experimental results for twist angle in CCW and CW chiral materials composed of 5 × 5 × 2 tessellated four‐cell chains under compression for e1) *α* = 60° and e2) *α* = 45°, e3) force–strain (black line) and torque–strain (red line) curves for CCW, CW, and CW‐CCW hybrid materials for *α* = 60°, e4) twist–strain curves of CCW (orange solid lines) and CW (blue dashed lines) materials for *α* = 45° and 60°, where the CCW rotation direction *φ*_*zz*_ is defined as the positive direction.

In addition to programming the chain, mechanical properties can be tuned effectively by deforming just the top part of the chain. From different material properties, chirality as an exotic behavior of metamaterials is chosen for tuning. We illustrate in **Figure** **8** that using the same deformation sequence logic, a novel designed chain can have left‐hand, right‐hand and neutral chirality.

The programmability of SMAs inspires us to also develop a reconfigurable architected material counterpart, based on the continuous path of multistable chains. The functionality of this design, however, is through the application of mechanical loads only at the top with no requirements for temperature increase (Section S4, Supporting Information). In this regard, a transversely‐bistable tensegrity‐like cell is initially designed by adding a string to a four‐bar linkage mechanism with one degree of freedom (Figure 8a1). The transverse force is obtained by:

(18) $$F=\frac{1}{2}TL_{0}\left(\frac{1}{l_{1}}-\frac{1}{l_{2}}\right)$$

where $l_{1}=L_{0}{\left[{\left(-1/2+\cos\theta \right)}^{2}+{\left(\sin\theta \right)}^{2}\right]}^{\frac{1}{2}}$ and $l_{2}=L_{0}\;{\left[{\left(1/2+\cos\theta \right)}^{2}+{\left(\sin\theta \right)}^{2}\right]}^{\frac{1}{2}}$, in which *θ* and *L*_0_ have been defined in Figure 8a1, and *T* = *kε* where *k* and *ε* are the longitudinal stiffness and strain (*ε*) of the string, respectively; in addition, $\varepsilon =\frac{l_{1}+l_{2}}{L_{1}+L_{2}}+\varepsilon^{*}$, in which ε* is the pre‐strain. While the tension of the string can tune the cell's force–displacement curve, the symmetry of the two stable configurations of this cell results in |*F*_max_| = |*F*_min_|. A multistable chain (Figure 8b), which can be tessellated to form an architected material, is designed by the unidirectional stacking of the transversely‐bistable cells. SMA crystal structures are convertible to twinned and detwinned. Analogous to SMAs, the transformation of the cells can change chain Bravais lattices between monoclinic and tetragonal (Figure 8b) (Section S4.2 and Movie S4, Supporting Information).

The mechanical convertibility is extended to *x* and *y* directions in Figure 8c, by stacking the designed unidirectional bistable cells in the order of instability forces as depicted in Figure 8c3 and having the assembled cells rotated +90 degrees about the *z*‐axis with respect to the previous cell (i.e., screw operation) (Section S4.3 and Movie S4, Supporting Information). As presented in Figure 8c4, externally applied deformations can transform these chains into clockwise (CW), counter‐clockwise (CCW), or CW‐CCW hybrid (half CW and half CCW) structures, each having different mechanical properties (Figure 8d). Tunable correlation between the twist angle *φ*_*zz*_ about the *z*‐axis and strain *ε*_*zz*_ along this direction is presented by:

(19) $$\varphi_{zz}=\kappa \left(N\right)\varepsilon_{zz}$$

where *κ*(*N*) depends on the stable state of the chain, and $N\in {\overset{︷}{\left\{\left({\underset{︸}{00\cdot \cdot \cdot 0}}_{n}\right)\right\},\;..,\left(11\cdot \cdot \cdot 1\right)}}^{2^{n}}$ is binary forms of 2^*n*^ possible stable configurations of the *n*‐cell chain. Such description illustrates the contribution of the shape transformation in tuning the chirality behavior, while a monostable chain is incapable of presenting such tunability. Three cellular structures close to the presented states of multistable chains (Figures 8c) have been designed to simplify the numerical and experimental evaluation of their chirality (Figure 8d,e). Finite element simulations confirm that the designed CW and CCW materials show counter‐clockwise and clockwise rotation in compression and vice versa in tension. This design also has directional shear shape transformation, which is obtained by half CW‐half CCW hybrid state. We demonstrate that stress flow depends on the chirality direction (right/left‐hand) of the material, regardless of the direction of the applied axial mechanical load (Figure 8d). Representative CW and CCW 3D chiral materials (*α* = 60°) show counter‐clockwise and clockwise rotation, respectively, with the angle of twists per axial compressive strain as large as 0.46°/%. Moreover, the CCW‐CW hybrid exhibits a coupled compression‐bending behavior. Versatile programmable properties associated with transversely‐multistable materials have also been investigated in Section S5, Supporting Information.

## 3 Conclusion

In conclusion, the programmability of mechanical metamaterials utilizing elastic instability has been hampered by the absence of a detailed understanding of their continuous paths. Presenting a simple and widely applicable strategy for determining the continuous paths in multistable structural systems provides a new platform for the realization of shape‐reconfigurable metamaterials and metastructures. We decipher how to deterministically program a multistable chain to control its elastic released energy and stable configurations in order to discover its latent functionalities as mechanical memories composed of elastic bits, sensors with integrated data‐reconstruction features, and architected materials with tunable chirality attributes. Our strategy may also be applied to nanoscale architected structures to open avenues for realizing high‐capacity mechanical memories triggered by a variety of actuations including magnetic,^[^ ^50^ ^]^ electrochemical,^[^ ^51^ ^]^ and capillary forces.^[^ ^52^ ^]^ Further research can contribute to the realization of compound motions of robotic arms and artificial muscles with multiple degrees of freedom by controlling a single force input on an exterior boundary (e.g., through pneumatic pressure), and consequently the deformation of constitutive snapping cells of a multistable chain. On the contrary, in a living muscle, contraction and relaxation of fibers result in one specific body motion^[^ ^43^ ^]^; accordingly, several muscles are needed to perform to cause a compound motion. As such, a rationally designed multimodal multistable chain can be utilized toeffectively substitute a complex system containing multiple muscles.

## Conflict of Interest

The authors declare no conflict of interest.

## Supporting information

Supporting Information

Supplemental Video 1

Supplemental Video 2

Supplemental Video 3

Supplemental Video 4

## Data Availability

The data that support the findings of this study are available in the supplementary material of this article.
