# Supplementary material for: Programming Multistable Metamaterials to Discover Latent Functionalities
Source: Adv Sci (Weinh). 2022 Oct 17;9(33):2202883. doi: 10.1002/advs.202202883 (PMC9685460; doi:10.1002/advs.202202883)
Supplement: Supplementary file 1 — Supporting Information [file ADVS-9-2202883-s004.pdf]

ADVANCED SCIENCE
Open Access

Supporting Information

for *Adv. Sci.*, DOI 10.1002/advs.202202883

Programming Multistable Metamaterials to Discover Latent Functionalities

*Hossein Mofatteh, Benyamin Shahryari, Armin Mirabolghasemi, Alireza Seyedkanani, Razieh Shirzadkhani, Gilles Desharnais and Abdolhamid Akbarzadeh**

Supporting Information

Programming Multistable Metamaterials to Discover Latent Functionalities

Hossein Mofatteh, Benyamin Shahryari, Armin Mirabolghasemi, Alireza Seyedkanani, Razieh Shirzadkhani, Gilles Desharnais, Abdolhamid Akbarzadeh*

* Corresponding Author, E-mail: hamid.akbarzadeh@mcgill.ca

Supporting Information includes:

S1. Theory, stable configurations, and released energy in a chain comprised of two cells
S2. Generalization of theory and mathematical method
S3. Multistability in tension/compression, and wave-like reconfiguration in multistable materials
S4. Multistability in shear and SMA-like materials for programming chiral materials
S5. Study of chirality in multistable metamaterials
S6. Application of structural multistability in memory devices and sensors
S7. Simulation method
S8. Manufacturing

Additional Supplementary Videos are available online:

Supplementary Movie S1. Shape reconfiguration of multistable chain (Deforming from the exterior boundary)
Supplementary Movie S2. Mechanical sensor
Supplementary Movie S3. Multistability, the chain of elastic bits, and arithmetic relations
Supplementary Movie S4. Multistability and tuning mechanical properties

S1. Theory, stable configurations, and released energy in a chain comprised two unit cells

S1.1. Solution algorithm

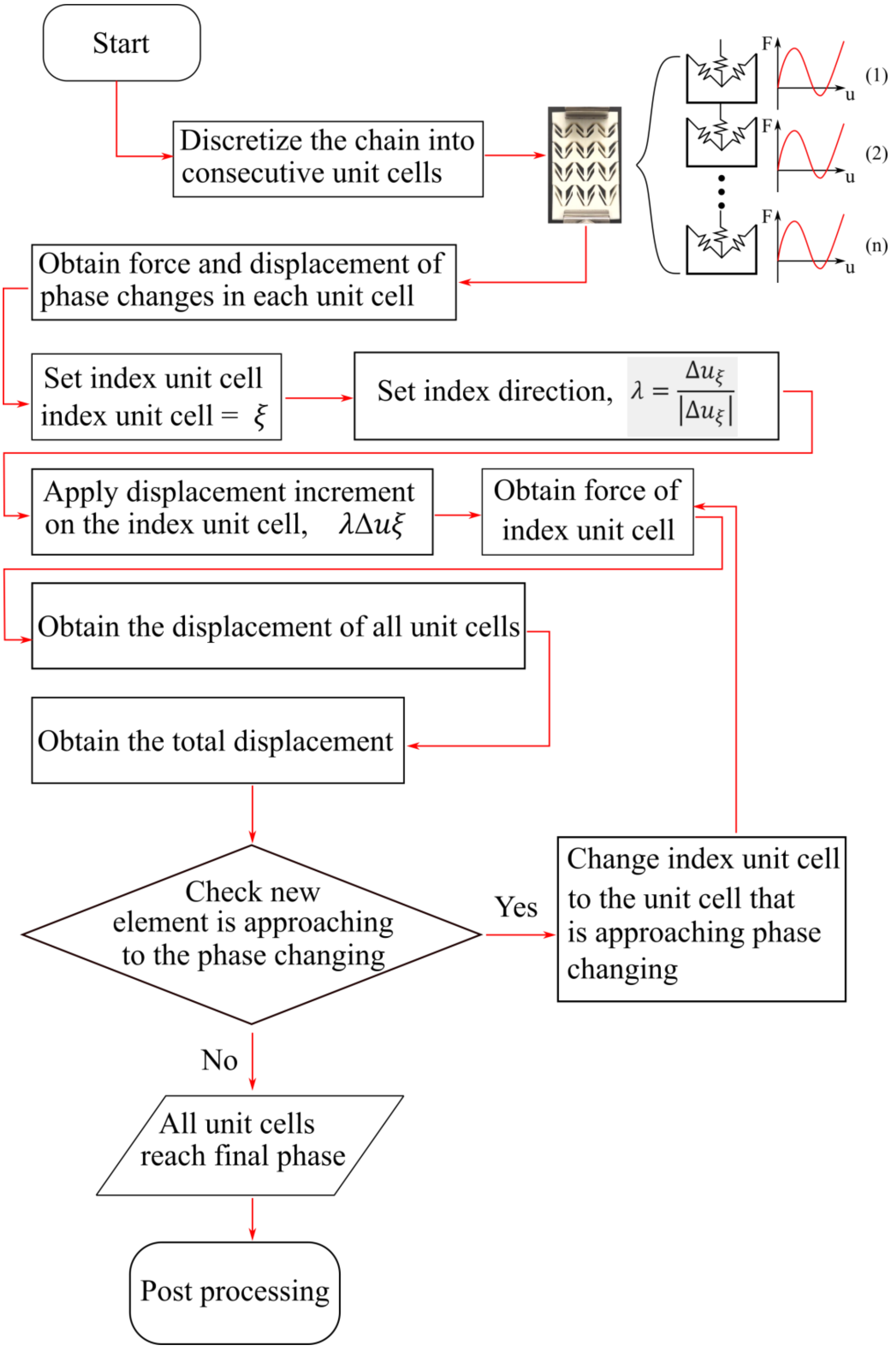

Figure S1. Solution algorithm to capture the continuous path of the chain and corresponding flowchart.

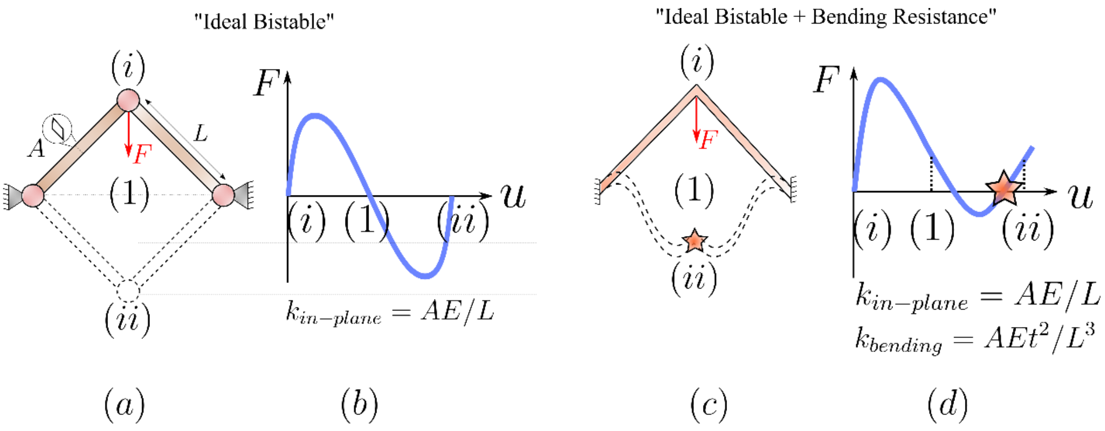

Figure S2. Illustration of **(a)** geometrical and **(b)** associated force-displacement curve of an idea bistable cell. **(c)** geometrical, and **(d)** force-displacement curve of a cell with the resistance stiffness.

Here, $k_{in-plnae}$, and $k_{bending}$ are the in-plane, and out-of-plane stiffness of the strut, respectively. A, t, L, E are the cross-section area, thickness, length, and Young's modulus of the strut, respectively.

S1.2. Chain comprised of two unit cells

S1.2.1. Case I (Combination of bistable and monostable cells)

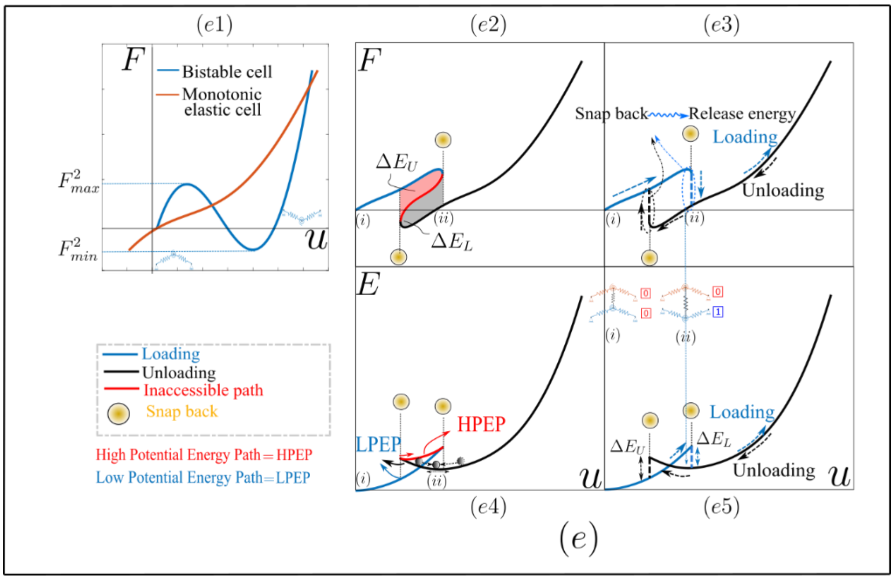

Figure S3. A chain is comprised of two unit cells, which the cells are characterized by monotonic soft and bistable cells. **(e1)** Force-displacement curve of the unit cells. **(e2)** and **(e3)** Theoretical force-displacement, and displacement control force-displacement curves, respectively. **(e4)** and **(e5)** Theoretical energy-displacement, and displacement control energy-displacement curves, respectively.

S1.2.2. Case II (Combination of two identical bistable cells)

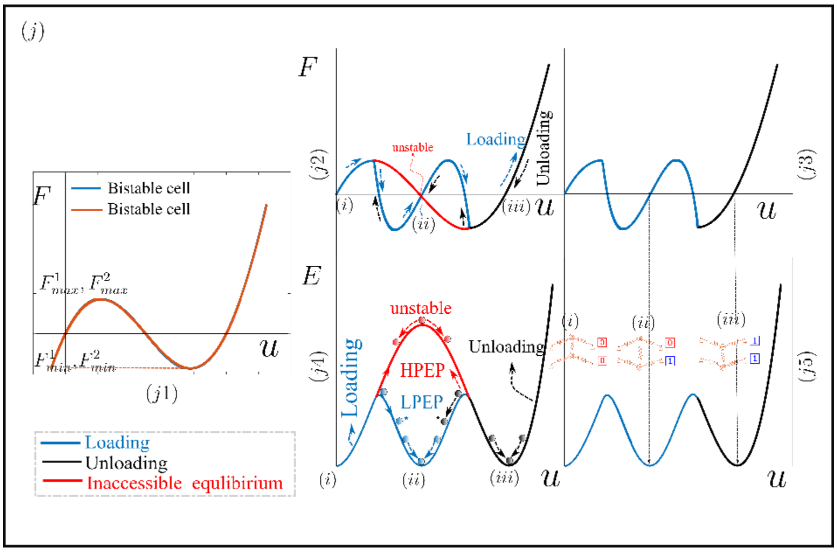

Figure S4. A chain is comprised of two unit cells, which the cells are characterized by $F^2_{max} = F^1_{max}$, $F^1_{min} = F^2_{min}$. **(j1)** Force-displacement curve of the unit cells. **(j2)** and **(j3)** Theoretical force-displacement, and displacement control force-displacement curves, respectively. **(j4)** and **(j5)** Theoretical energy-displacement, and displacement control energy-displacement curves, respectively.

S1.2.3. Case III (Combination of two bistable cells, characterized by $F^2_{max} < F^1_{max}$ and $F^1_{min} < F^2_{min}$, and with the same periodicity)

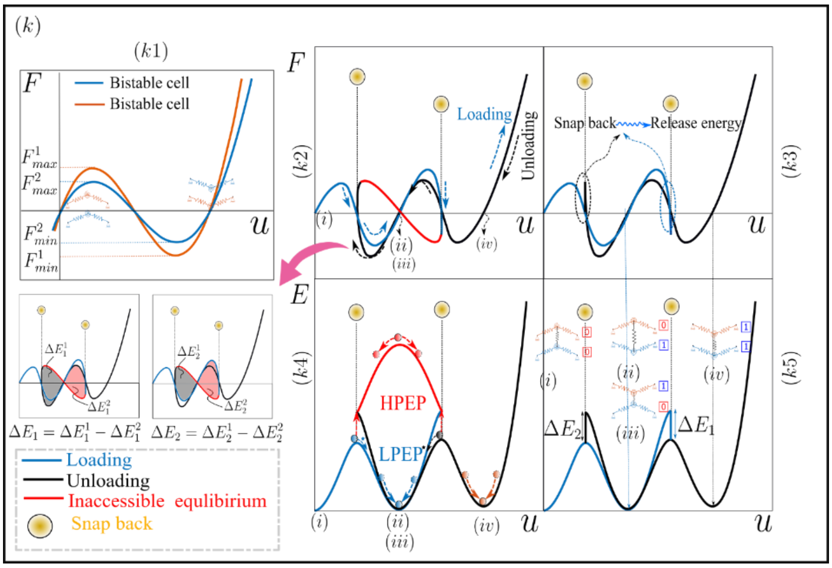

Figure S5. A chain is comprised of two unit cells, which the cells are characterized by $F^2_{max} < F^1_{max}$, $F^1_{min} < F^2_{min}$. **(k1)** Force-displacement curve of the unit cells. **(k2)** and **(k3)** Theoretical force-displacement, and displacement control force-displacement curves, respectively. **(k4)** and **(k5)** Theoretical energy-displacement, and displacement control energy-displacement curves, respectively.

S1.2.4. Cases IV and V (Combination of two bistable, characterized by $F^2_{max} < F^1_{max}$ and $F^2_{min} < F^1_{min}$, and with different periodicity)

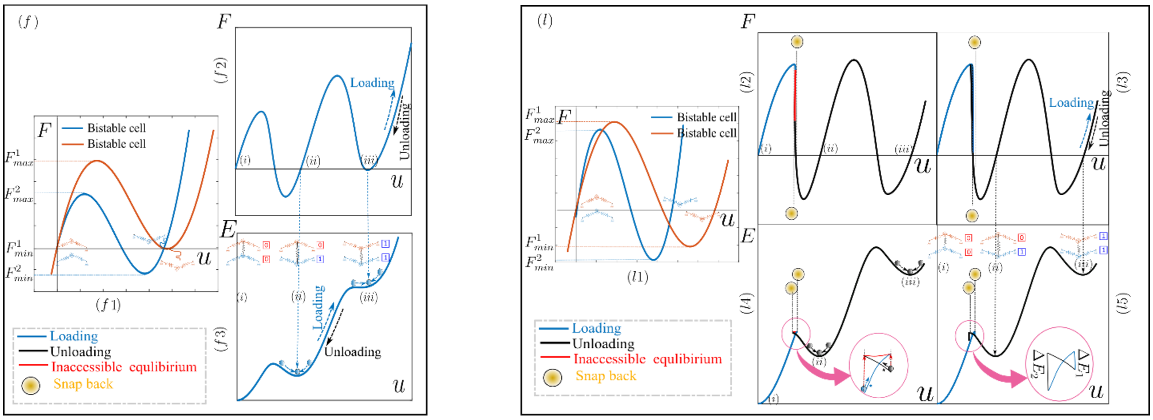

Figure S6. A chain comprised of two unit cells, in which the cells are characterized by **(f)** $F^2_{max} < F^1_{max}$, $F^2_{min} < F^1_{min}$, where cell I is marginally bistable and **(l)** $F^2_{max} < F^1_{max}$, $F^2_{min} < F^1_{min}$, respectively. **(f1, l1)** Force-displacement curve of the unit cell. **(f2, l2)** and **(l3)** Theoretical force-displacement, and displacement control force-displacement curves, respectively. **(f3, l4)** and **(l5)** Theoretical energy-displacement and displacement control energy-displacement curves, respectively.

S1.2.5. Case VI (Combination of two bistable cells, characterized by $F^2_{max} < F^1_{max}$, and $F^1_{min} < F^2_{min}$, and with different periodicity)

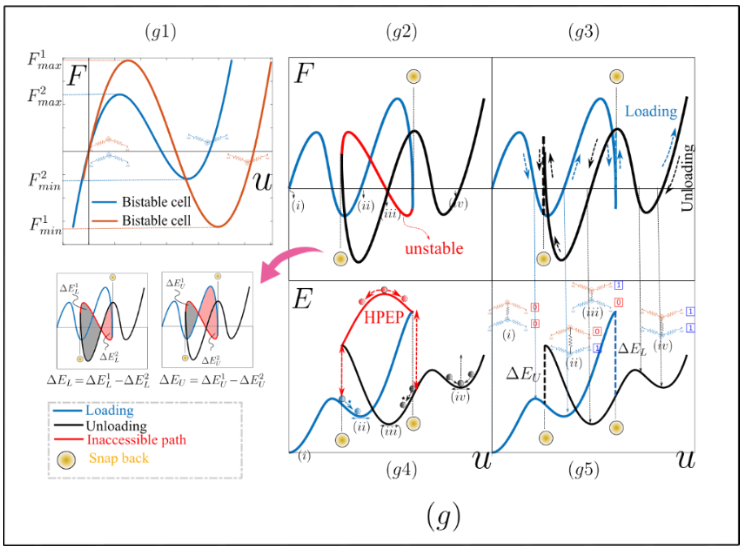

Figure S7. A chain is comprised of two unit cells, which the cells are characterized by $F^2_{max} < F^1_{max}$, $F^1_{min} < F^2_{min}$. **(g1)** Force-displacement curve of the unit cell. **(g2)** and **(g3)** Theoretical force-displacement, and displacement control force-displacement curves, respectively. **(g4)** and **(g5)** Theoretical energy-displacement, and displacement control energy-displacement curves, respectively.

S1.3. Programming the released energy

The released energy in a chain has been studied in Figure S8. The chain comprises two unit cells, which the cells are characterized by monotonic soft and bistable cells.

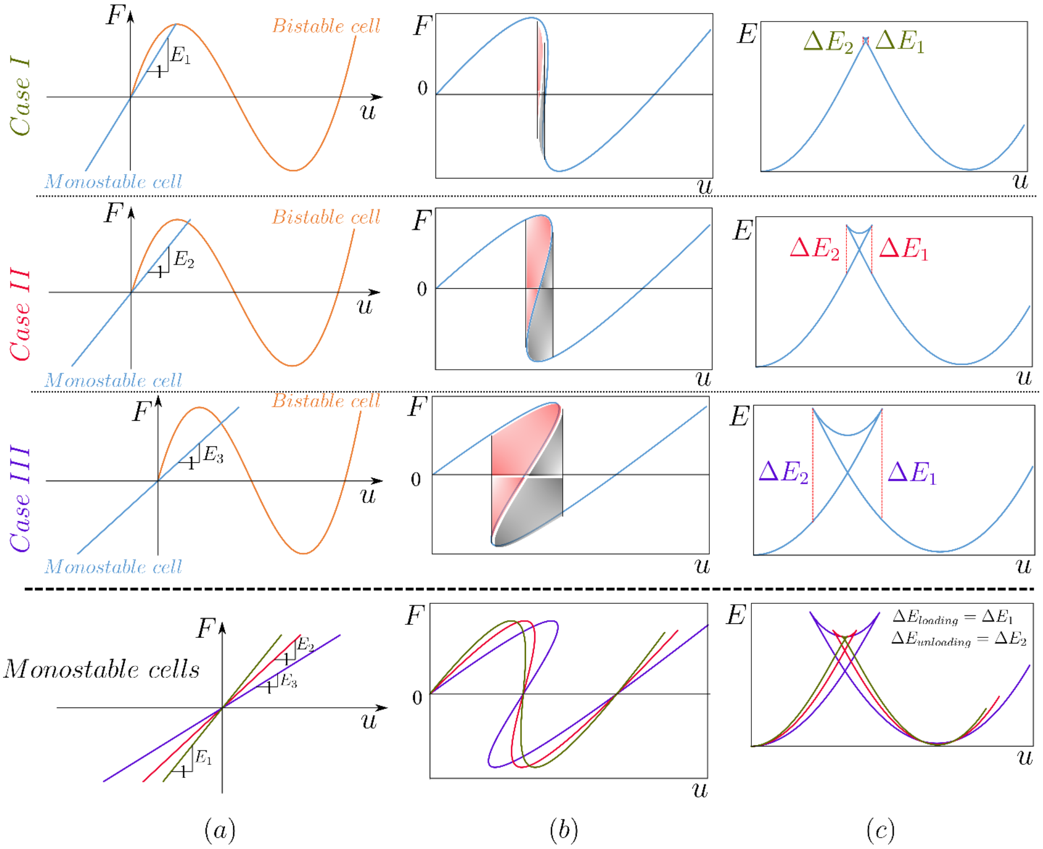

Figure S8. A chain comprises two unit cells, which the cells are characterized by monotonic soft and bistable cells. **(a)** force-displacement curves of the cells. **(b)** theoretical force-displacement of the chains, and **(c)** theoretical energy-displacement curves of the chain in which $k_{Mono}^{case\ III} < k_{Mono}^{case\ II} < k_{Mono}^{case\ I}$, leading $\Delta E_i^{case\ I} < \Delta E_i^{case\ II} < \Delta E_i^{case\ III}$, where $i = 1,2$.

The released energy in a chain has been studied in Figure S9. The chain comprises two unit cells, which the cells are characterized by $F_{max}^2 < F_{max}^1$, $F_{min}^1 < F_{min}^2$.

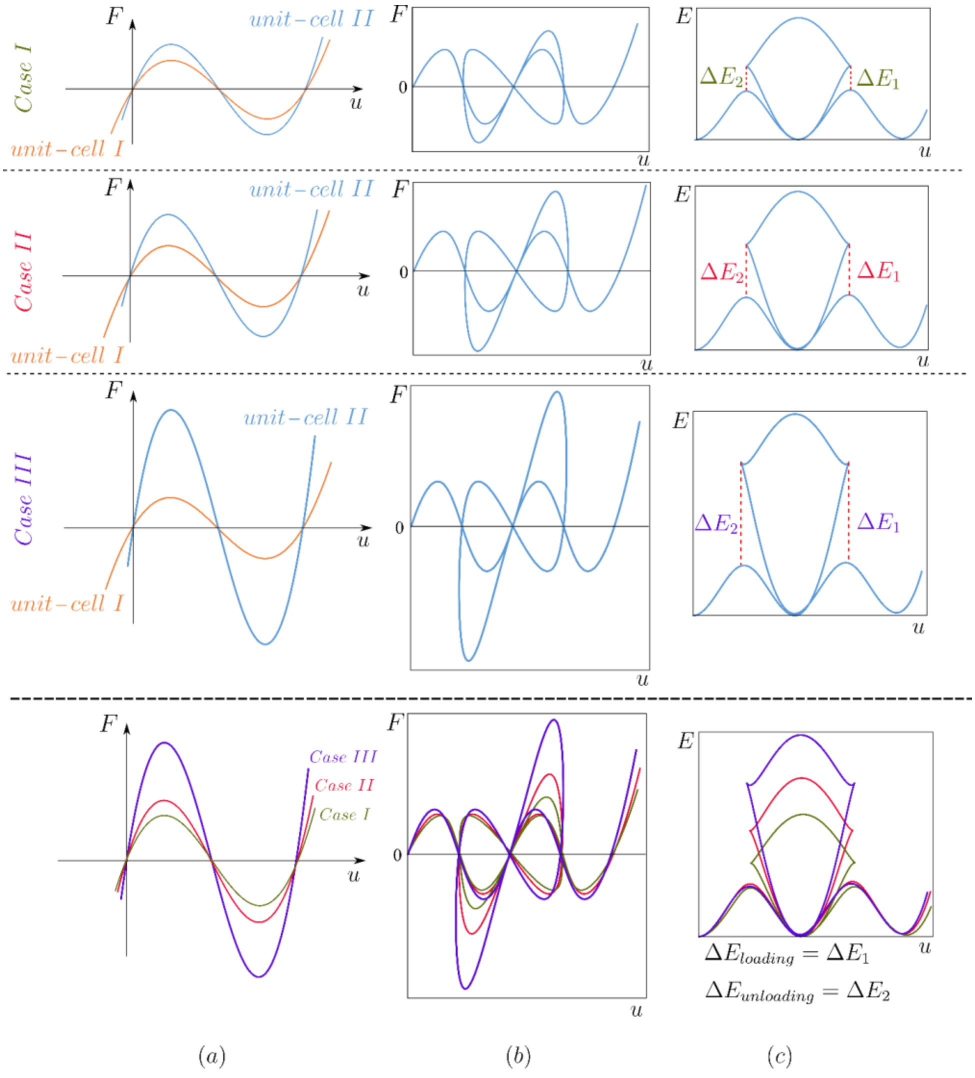

Figure S9. A chain comprises two unit cells, which the cells are characterized by $F_{max}^2 < F_{max}^1$, $F_{min}^1 < F_{min}^2$. **(a)** force-displacement curves of the cells. **(b)** theoretical force-displacement of the chains, and **(c)** theoretical energy-displacement curves of the chain in which $(|F_{max}^1 - F_{max}^2| + |F_{min}^1 - F_{min}^2|)^{case\ I} < (|F_{max}^1 - F_{max}^2| + |F_{min}^1 - F_{min}^2|)^{case\ II} < (|F_{max}^1 - F_{max}^2| + |F_{min}^1 - F_{min}^2|)^{case\ III}$, leading $\Delta E_i^{case\ I} < \Delta E_i^{case\ II} < \Delta E_i^{case\ III}$, where $i = 1{,}2$.

S1.4. Energy dissipation relation with the negative stiffness of instabilities

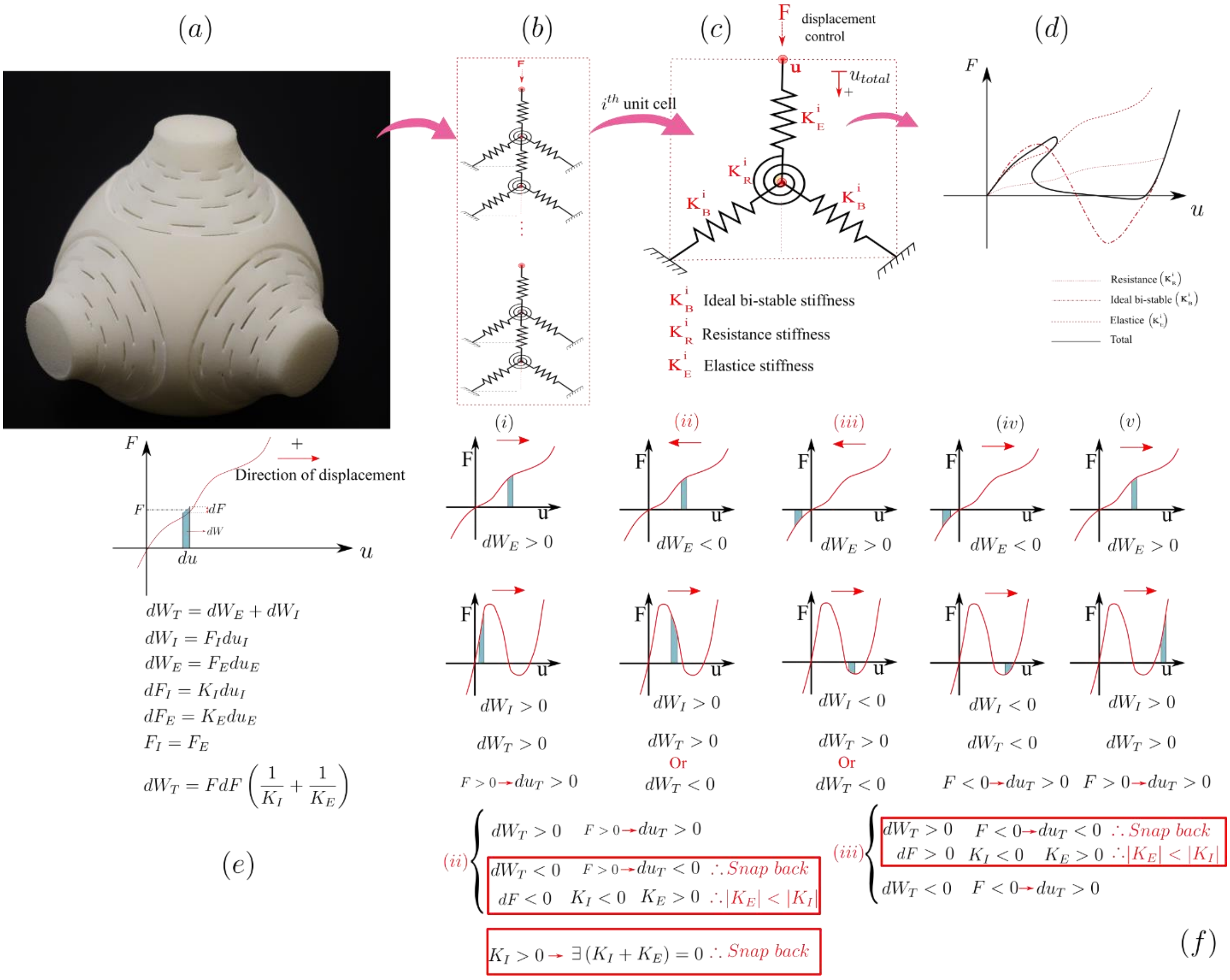

Figure S10. Energy dissipation relation to the negative stiffness of instabilities**. (a)** three directional multistable materials. **(b)** Illustrations of the conventional model of bistable chain composed of bistable elements. **(c)** modeling of bistable cell with snap-through (instable) and elastic (stable) parts which any snap-through part can be conceptually divided into an ideal bistable and resistance parts. **(d)** corresponding force-displacement curves of the defined springs. **(e)** nomenclature of elastic, snap-through, and total energies to investigate the snapping back points. **(f)** required and sufficient conditions to design a chain by which the snapping back occurs.

We extend the conventional model for a chain composed of bistable cells and theoretically divide the stiffness of each constitutive bistable cell into snap-through (instable) and monotonic parts. The snap-through part can be conceptually decomposed into an ideal bistable element connected in parallel with a bending resistant element. The ideal bistable part has a symmetrical force-displacement curve, which implies the stable configurations have the same zero elastic energy level; as an example, a tilted hinged beam, shown in Figure S2, demonstrates an ideal bistable behavior. The bending resistant part has a positive definite stiffness, transforming the snap-through behavior from ideal bistability towards marginal bistability and eventually to a monostable behavior. For instance, clamped constraint at the end of the titled beam imposes the

bending resistance (Figure S2). The monotonic (elastic) part with a positive definite stiffness in series with the snap-through part can cause the snap-back behavior. In summary, the snap-through part comprises positive and negative stiffness , while the elastic part always imposes a positive stiffness on the cell. Therefore, any non-monotonic cell might be in a negative or positive stiffness state, and even more, it continuously passes through these states.

S1.5. Energy dissipation relation with instability forces and the negative stiffness of instabilities

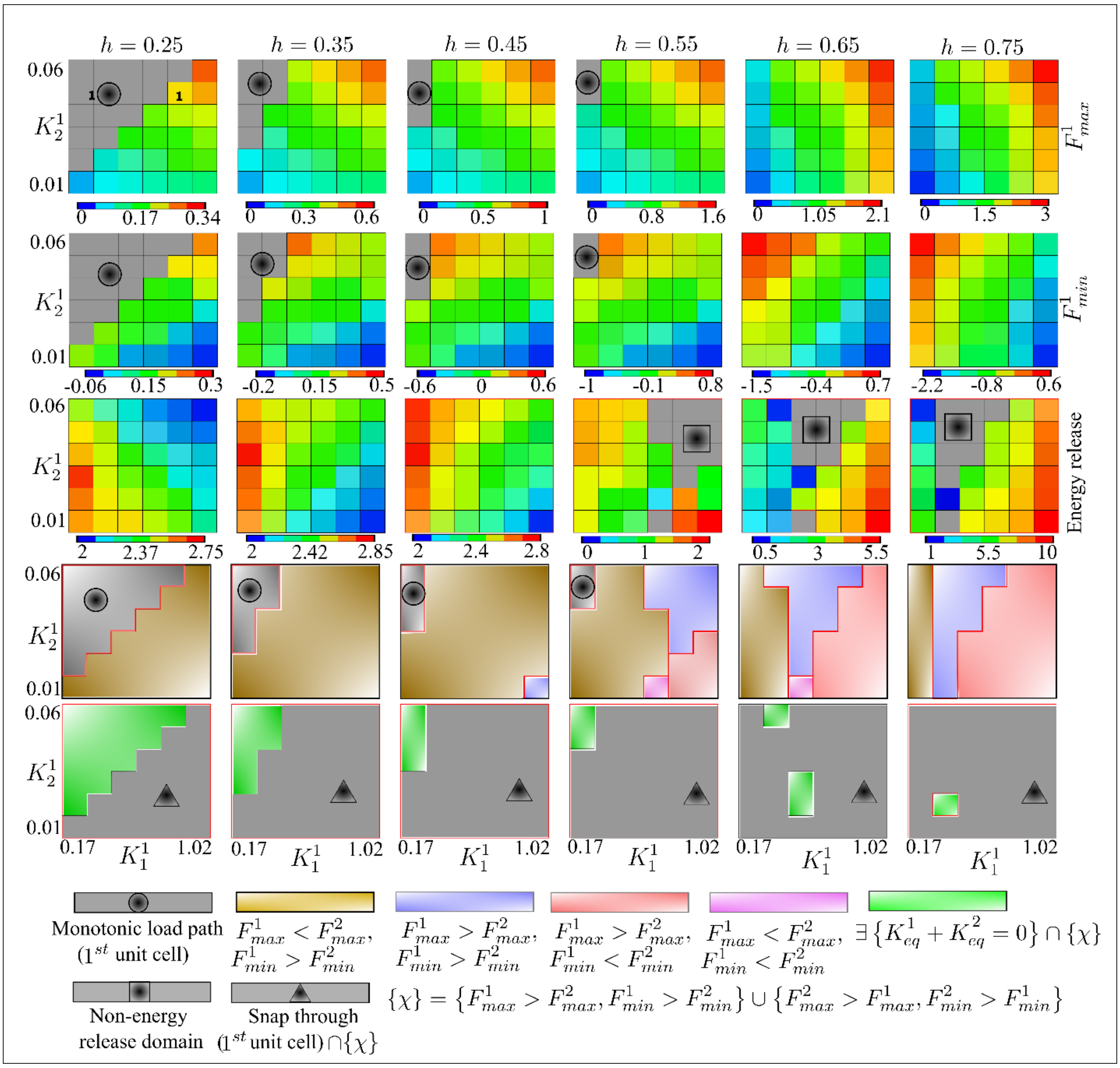

Figure S11. Energy dissipation relation to instability forces and the negative stiffness of instabilities in a chain that is comprised of two unit cells. All the quantities for one of the cells have been normalized to the properties of another cell, where K_1^1, and K_2^1 are the normalized resistance and ideal bistable stiffness, F_{max}^1, and F_{min}^1 are the normalized minimum and maximum forces of the cell, h is the ratio of the height to length of the cell, and the ratio for the base cell is 0.5. **(a)**, **(b)**, and **(c)** variation of F_{max}^1, F_{min}^1, and released energy relative toK_1^1 (horizontal axis) and K_2^1(vertical axis) for constant h, respectively. **(d)** released energy in a chain comprises two bistable unit cells,

which are characterized by $F_{max}^2 < F_{max}^1,\ F_{min}^1 < F_{min}^2$ (red area), and $F_{max}^2 > F_{max}^1,\ F_{min}^1 > F_{min}^2$ (gold area) and by increasing $|F_{max}^1 - F_{max}^2| + |F_{min}^1 - F_{min}^2|$, the released energy increases, which in this case is pertinent to Figure 2(c). Also, the gray shaded area demonstrates the released energy in a chain that comprises one bistable and monostable unit cell. By increasing the stiffness of monostable cells, the released energy decreases. This case corresponds to Figure 2(a) in the main text. **(e)** the green area shows the released energy when snap-back happens, and the equivalent stiffness of the cells will be zero. This area is pertinent to two cases. First, the chain comprises a bistable and monostable unit cell. Second, the chain comprises two bistable unit cells, which are characterized by $F_{max}^2 < F_{max}^1,\ F_{min}^1 > F_{min}^2$, or by $F_{max}^2 > F_{max}^1,\ F_{min}^1 < F_{min}^2$, in which these cases are pertinent to Figure 2(b) in the main text.

S1.6. Results summary of 1D chain composed of two unit cells

required conditions for stable configurations and snapping energy of 1D chain comprised of two unit cells have been discussed. In summary, these figures allow us to select the appropriate geometry to achieve different designs aligned with our concern in programming multistable metamaterials. For example, how would it be possible to maximize/minimize the released energy? Answering this question paves the way for designing a soft jumper [1], energy absorption element [2], and more interestingly, how to achieve each configuration; answering this question provides a foundation for designing a new generation of mechanical sensors and memory devices [3].

Considering that $0 < F_{max}^2 < F_{max}^1$, Figure 1(b) shows necessary and sufficient conditions to design a multistable 1D chain. Clearly, if both cells are monostable ($0 < F_{min}^1,\ and\ 0 < F_{min}^2$), the chain is monostable; while if one of the cells is bistable and the other cell is monostable ($0 < F_{min}^1,\ and\ F_{min}^2 < 0$ or $F_{min}^1 < 0,\ and\ 0 < F_{min}^2$), the chain will be bistable which corresponds to Figure 2(a). If $F_{min}^2 < F_{min}^1 < 0$, three stable configurations will be captured in loading and unloading paths related to Figs. 2(b). Finally, if all the required conditions are satisfied, which means $0 < F_{max}^2 < F_{max}^1$, and $F_{min}^1 < F_{min}^2 < 0$, four stable configurations will be unveiled, which corresponds to Figure. 2(c).

More interestingly, Figure 4 has been presented to pave the way for programming the released energy in a chain corresponding to Figure 2(a) and Figure 2(c), respectively. As shown, in the case pertinent to Figure 2(a), stiffness of the monostable cell is the most effective parameter in which by increasing the stiffness, the released energy decreases, while in the case related to Figure 2(c), the $|F_{max}^1 - F_{max}^2| + |F_{min}^1 - F_{min}^2|$, is the most effective factor in which by increasing it, the snapping energy increases. In other words, in the case pertinent to Figure 2(a), released energy increases by softening of the monostable cell, which has already been studied for soft jumper application. However, we are looking to harness high released energy along with high overall stiffness in stable configurations. As shown in Figure 2(c), by selecting two

bistable elements with the order $F_{max}^{2} < F_{max}^{1}$, $F_{min}^{1} < F_{min}^{2}$ and also increasing the $|F_{max}^{1} - F_{max}^{2}| + |F_{min}^{1} - F_{min}^{2}|$, we can make stiff material with high released energy.

S1.7. 2D and 3D multistability

2D chain is comprised of two cells in two orthogonal directions. Note that the cells are independent.

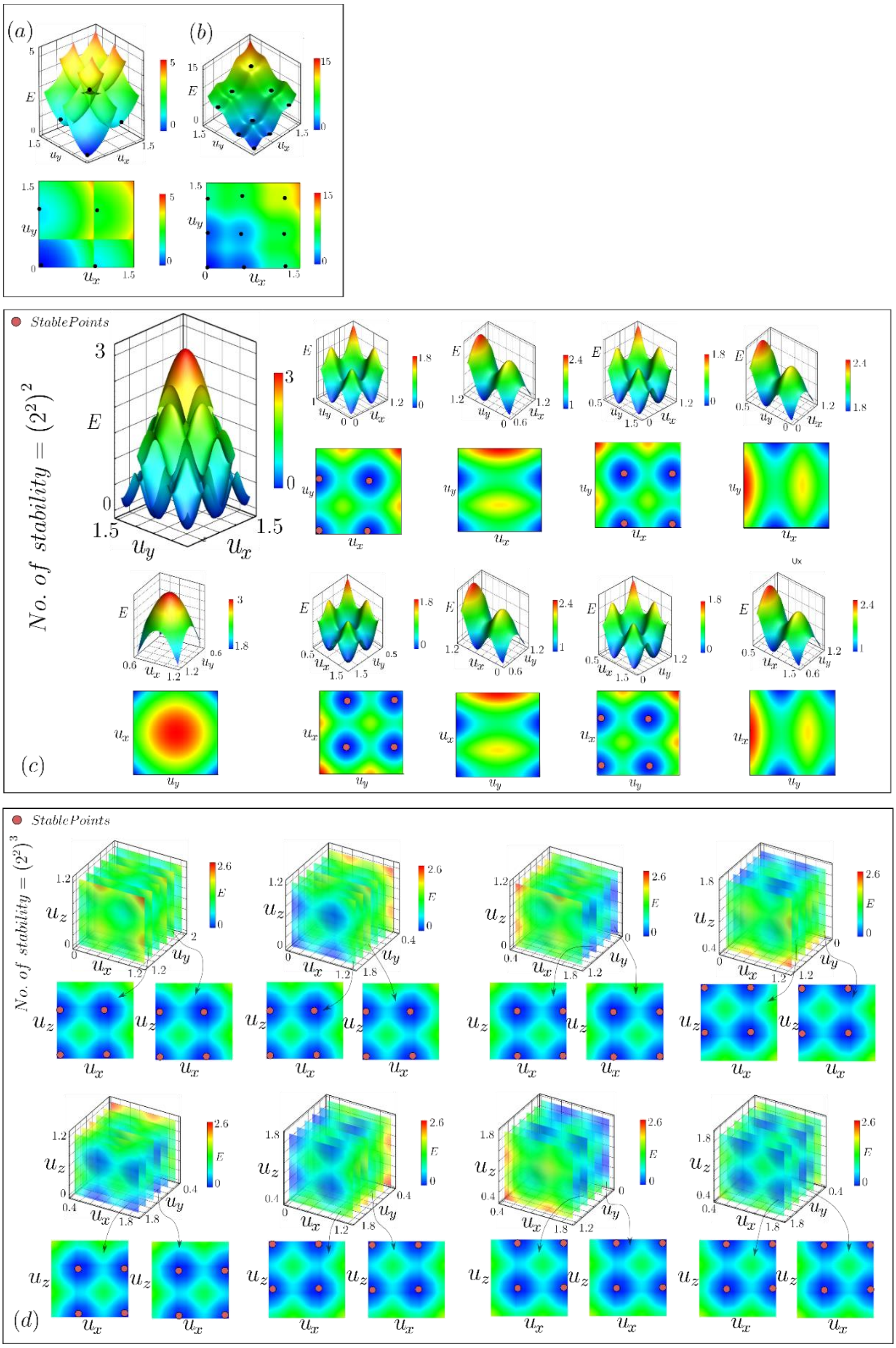

Figure S12. 2D Multistability. The 2D chain is comprised of two cells tesselated in two orthogonal directions. **(a)** The chain possesses a monostable and a bistable cell in each direction. Therefore, four stable configurations have

been recognized and illustrated by the black points that are the local minima of the potential energy. **(b)** The chain possesses two bistable unit cells in each direction characterized by $F_{max}^2 < F_{max}^1$ and $F_{min}^2 < F_{min}^1$ (one discrepancy in each direction), and demonstrates nine bistable configurations. **(c)** The chain possesses two bistable unit cells in each direction characterized by $F_{max}^2 < F_{max}^1$ and $F_{min}^1 < F_{min}^2$ (no discrepancy), and 16 stable configurations and the corresponded local minima have been illustrated in four parts of the energy-displacement curve. **(d)** 3D Multistability**.** The chain is characterized by $F_{max}^2 < F_{max}^1$, $F_{min}^1 < F_{min}^2$, and 64 stable configurations and the corresponded local minima have been illustrated in 16 parts of the energy-displacement curve. The stable configurations in **(c)**, and **(d)** have been illustrated by pink points.

S1.8 experimental evaluation of the theory for different combinations of two unit cells.

Seven unit cells have been designed and 3D printed by fused deposition modeling (FDM) using an Ultimaker S3 3D printer. The middle part of the unit cell is printed out of thermoplastic polyurethane (TPU (95A)), while the outer part is made out of polylactic acid (PLA) as support.

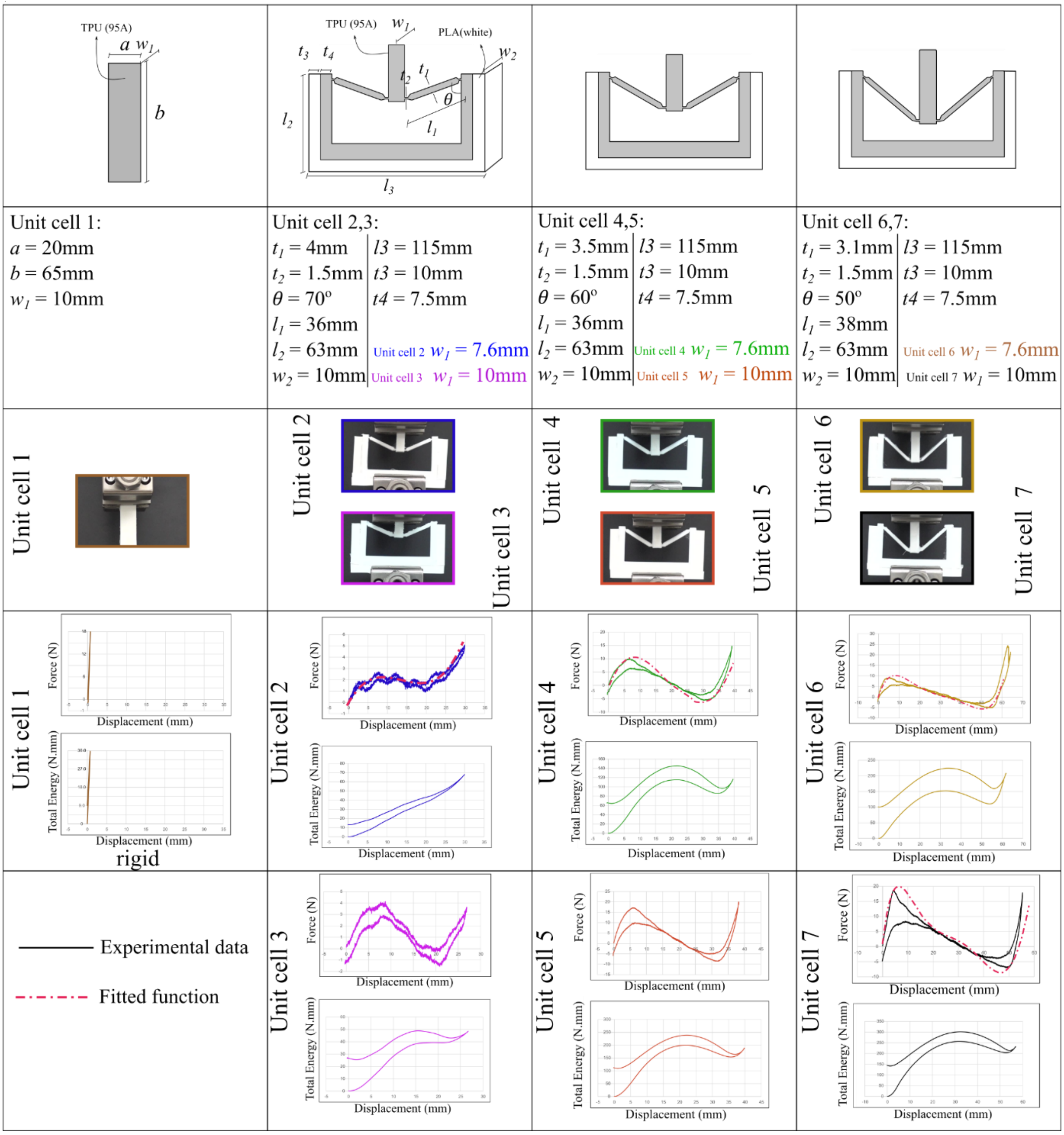

Figure S13: Geometrical parameter, force-displacement, and energy displacement curves of 3D printed unit cells. The observed energy dissipation in the total energy-displacement curves is associated with material energy dissipation (E_D).

Table S1. Parameters of fitted force-displacement for each unit cell.

$F = -au^6 + bu^5 - cu^4 + du^3 - eu^2 + fu$						
	a	b	c	d	e	f
Cell 1	0	0	0	0	0	36.000
Cell 2	0	0	0	1.117×10^{-3}	4.761×10^{-2}	5.986×10^{-1}
Cell 4	0	0	2.000×10^{-5}	4.500×10^{-3}	2.051×10^{-1}	2.464
Cell 6	0	1.489×10^{-6}	2.131×10^{-4}	1.141×10^{-2}	2.782×10^{-1}	2.643
Cell 7	6.949×10^{-8}	1.468×10^{-5}	1.169×10^{-3}	4.449×10^{-2}	8.100×10^{-1}	6.288

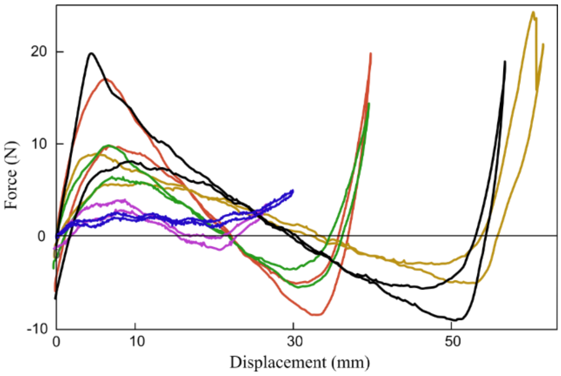

Figure S14. The force-displacement curve for different unit cells to compare the instability forces.

And six combinations with one reference cell (bottom one (unit cell 4)) have been studied as follow:

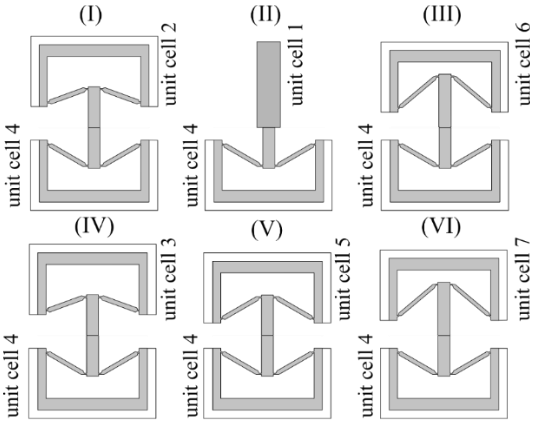

Figure S15. Six combinations of the designed unit cells. To make the comparison possible, the bottom cell was kept constant (unit cell 4), and six combinations with the other six unit cells were obtained.

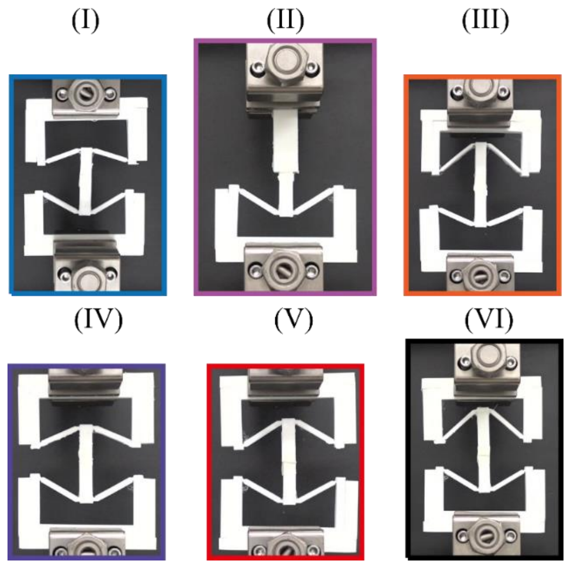

Figure S16. Printed combinations of reference bistable cell with (I) soft monostable, (II) rigid part, and (III-VI) low-to-high instability forces.

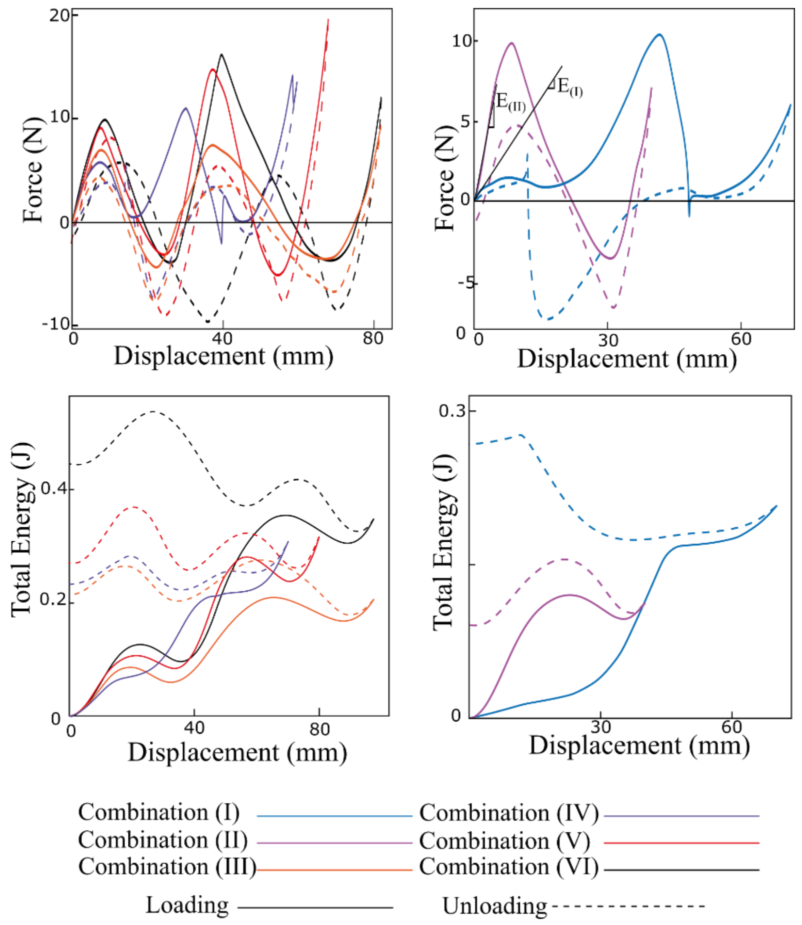

Figure S17. Force displacement and energy displacement behavior for six combinations.

S2. Generalization of theory and mathematical method

In the previous figures, the actual loading and unloading path, inaccessible equilibrium path, snap-back, released energy, and the number of possible stable configurations were discussed. Since we are interested in finding stable equilibrium configurations, we turned our attention to investigating the required conditions for proposing, designing, and programming a chain with more than two cells. In this regard, perusing the actual path and releasing energy during the loading and unloading are our outstanding concerns. Therefore, Figure 5. in the main text has been demonstrated. Here, a more informative and underlying point is unveiled; what are the effective parameters in the number of configurations, and how can it change the configurations? This question has remained unanswered. In recent years, many studies have been done to predict the configurations. Nevertheless, due to the innate weakness of the approach, the researchers could not capture the configurations because their analyses would not provide a comprehensive and realistic description. Eventually, a new perspective has been provided in this research; motivated by our robust methodology based on the deformation sequences, the possible configurations for a chain have been revealed. Furthermore, we systematically extended our methodology to study various cases not depicted in Figure 5 of the main text.

It should be noted that more unit cells provide more options for transition, which increases the possibility for configurations. Increasing the structure's size and complexity makes the systems' description difficult. Hence, we introduce another influential part of our novel method and description of reconfigurable metamaterials. More interestingly, based on the pascal triangle and set theory, mathematical concepts have been employed to systematically explore the number of stable configurations and necessary and sufficient conditions to achieve the configurations.

S2.1. Model of a 1D chain comprised of three bistable unit cells

In this section, the response of the constitutive cells of the chain is discussed during the loading and unloading. As illustrated in Figure S18, the force-displacement curve of each unit cell is divided into three-phase identified phases I, phase II, and phase III, respectively. Also, points (i), (1), and (ii) are pertinent to the initial stability, instability, and second stability points. In phase I, cells are prone to return to their initial configuration (point (i)). In phase II, cells are unstable and can be located at point (1); herein, two scenarios occur. First, by applying forward controlled displacement during which the cell eventually goes to the point with the minimum force at the beginning of phase III; second, by applying the backward controlled displacement, the cell moves back to the maximum force at the end of phase I. In the first scenario, cells will be stable in the second configuration, whereas, in the second scenario, the cells will be stable

in the initial configuration. Therefore, each cell's maximum and minimum forces are the boundaries of the phases, and by achieving these forces, the phase of cells switches.

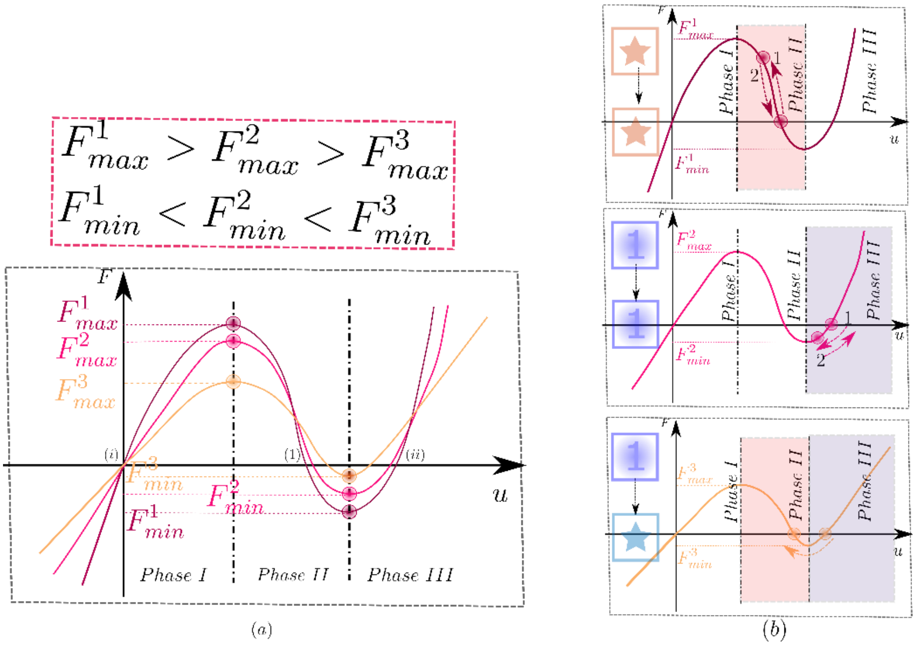

Figure S18. (a) Illustration of the ideal combination of the maximum and minimum forces in a chain comprised of three bistable unit cells, and the defined phases where the boundaries of phases I and II, and phases II and III are defined by the points associated with the maximum force and minimum force. **(b)** Schematic of the phase changing in the chain based on the orders of the F_{max} and F_{min}, in which by applying a backward controlled displacement, cell 3 returns from phase III to phase II, while cells I and II remain in their phases.

For a better interpretation of the theory, it is worth mentioning that the cells are in a series arrangement in a chain; therefore, the force in each cell is equal to the force of the chain. This fact, accompanied by dividing the force-displacement curve into three phases, is required to find a continuous loading and unloading path. When $F^1_{min} < F^2_{min} < F^3_{min}$, applying a reversed controlled displacement moves cell III backward from phase III to phase II while cells I and II remain in their current phase. Therefore, it clearly shows that the minimum and maximum forces are the governing parameters in finding the loading and unloading paths of the chain.

S2.1.1. Displacement control force-displacement curve

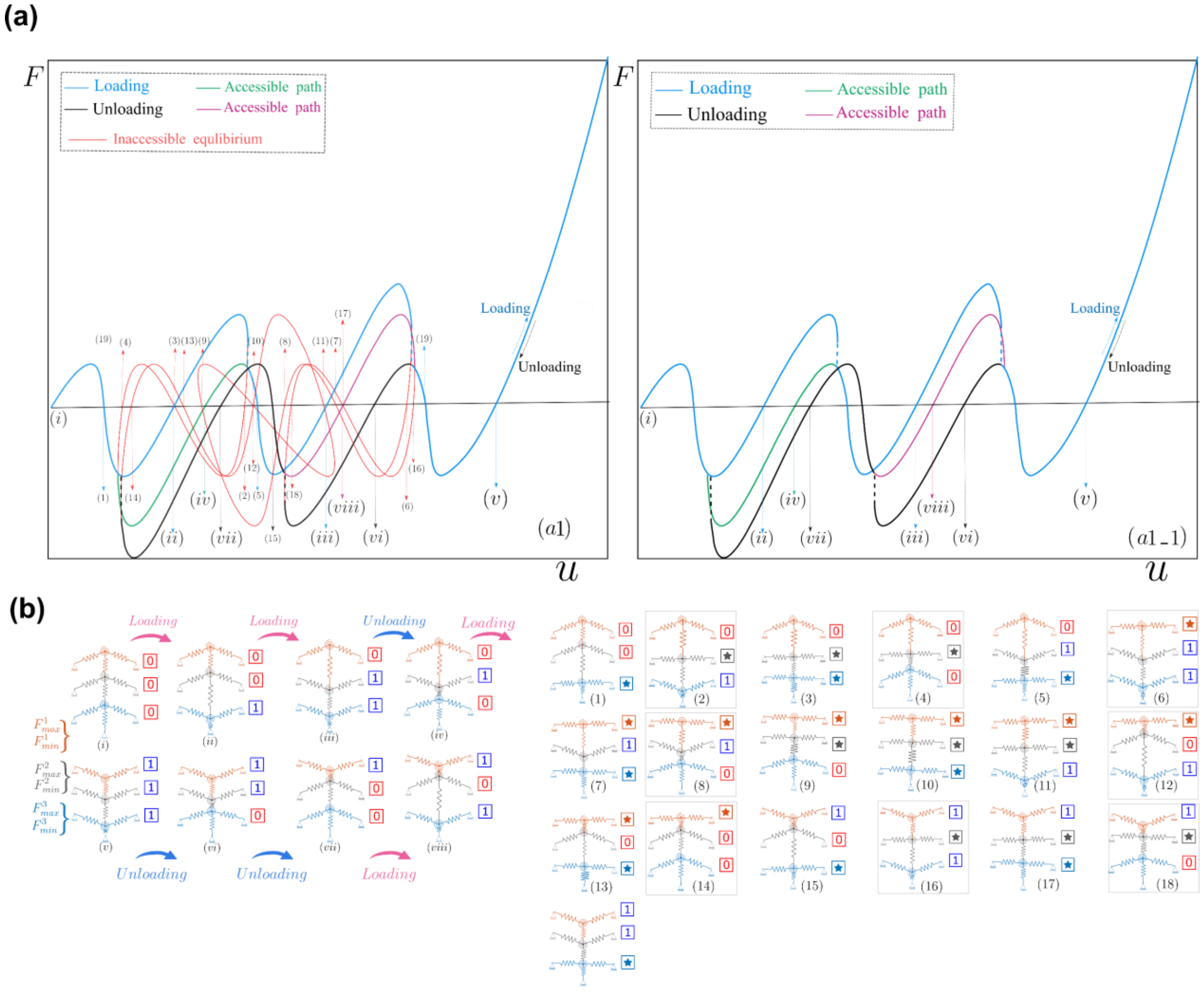

Figure S19. A chain comprises three bistable unit cells, characterized by $F^3_{max} < F^2_{max} < F^1_{max}, and\ F^1_{min} < F^2_{min} < F^3_{min}$, in which, the blue and black lines demonstrate the loading and unloading paths, respectively, and the green and purple lines illustrate the accessible paths in loading and unloading paths which capture two distinct stable configurations. **(a1)** force-displacement of the chain in which the blurred red line shows the inaccessible path. **(a1-1)** displacement control force-displacement curve of the chain in which the drops of the curve in snapping back are illustrated by dashed lines. Also, $(i) - (viii)$ show the stable configurations, and $(1) - (19)$ depict the unstable configurations in the chain. **(b)** schematic illustration of the stable and unstable configurations, in which zero and one show the initial and second stable configurations, respectively, and the star sign illustrates the unstable configuration of the pertinent cell. Also, the way to capture all the stable configurations based on the orders of F_{max},and F_{min}, without investigating the force-displacement curve, has been presented.

S2.1.2. Continuous force-displacement curve

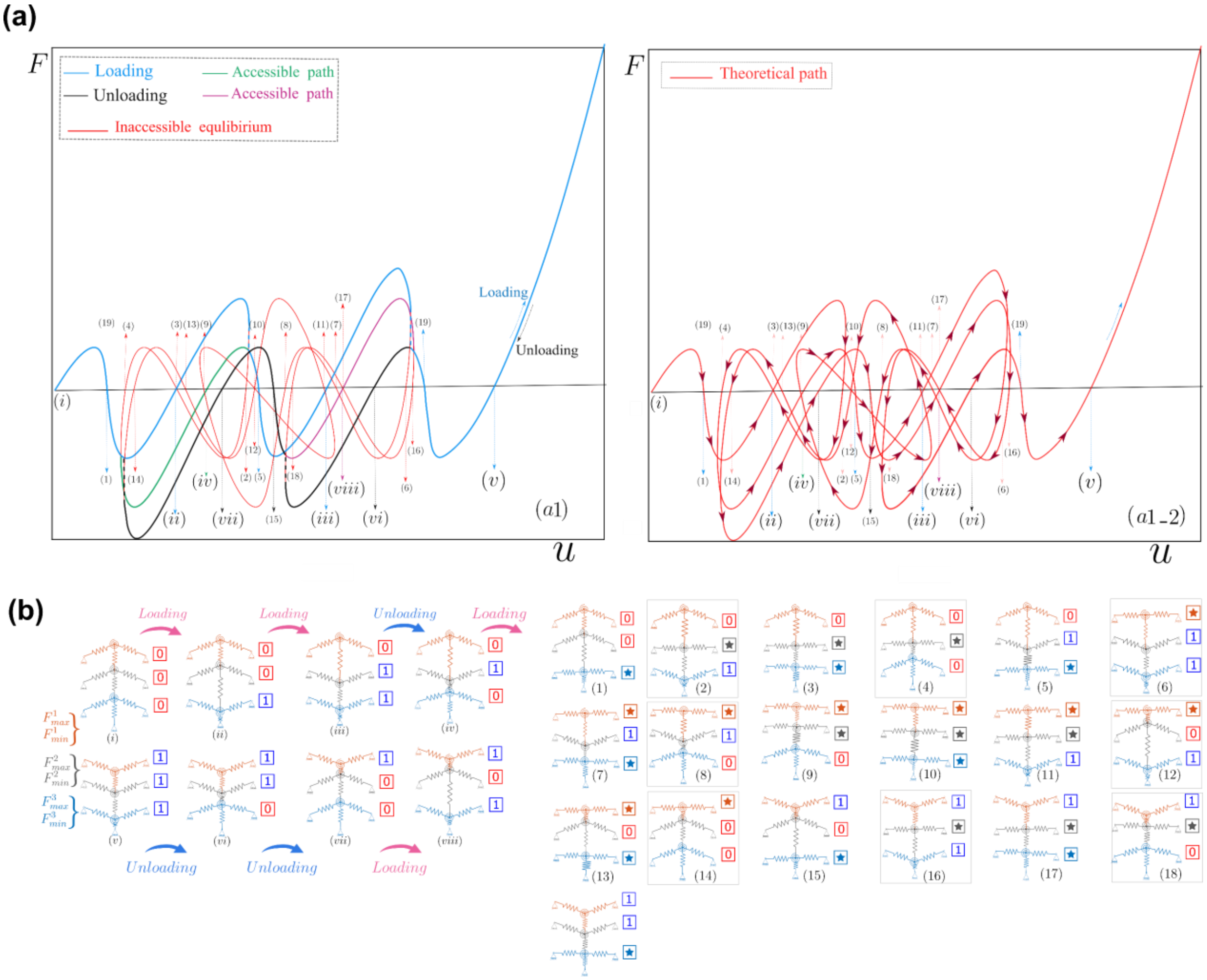

Figure S20. A chain comprises three bistable unit cells, characterized by $F^3_{max} < F^2_{max} < F^1_{max}, and\ F^1_{min} < F^2_{min} < F^3_{min}$, in which, the blue and black lines demonstrate the loading and unloading paths, respectively, and the green and purple lines illustrate the accessible paths in loading and unloading paths which capture two distinct stable configurations. **(a1)** force-displacement of the chain in which the blurred red line shows the inaccessible path. **(a1-2)** continuous force-displacement path of the chain in which the red line illustrates the path, and arrows show the direction of the continuous path in loading. Also, $(i) - (viii)$ show the stable configurations, and $(1) - (19)$ depict the unstable configurations in the chain. **(b)** schematic illustration of the stable and unstable configurations, in which zero and one shows the initial and second stable configurations, respectively, and the star sign illustrates the unstable configuration of the pertinent cell. Also, the way to capture all the stable configurations based on the orders of F_{max}, and F_{min}, without investigating the force-displacement curve, has been presented.

S2.1.3. Displacement control energy-displacement curve

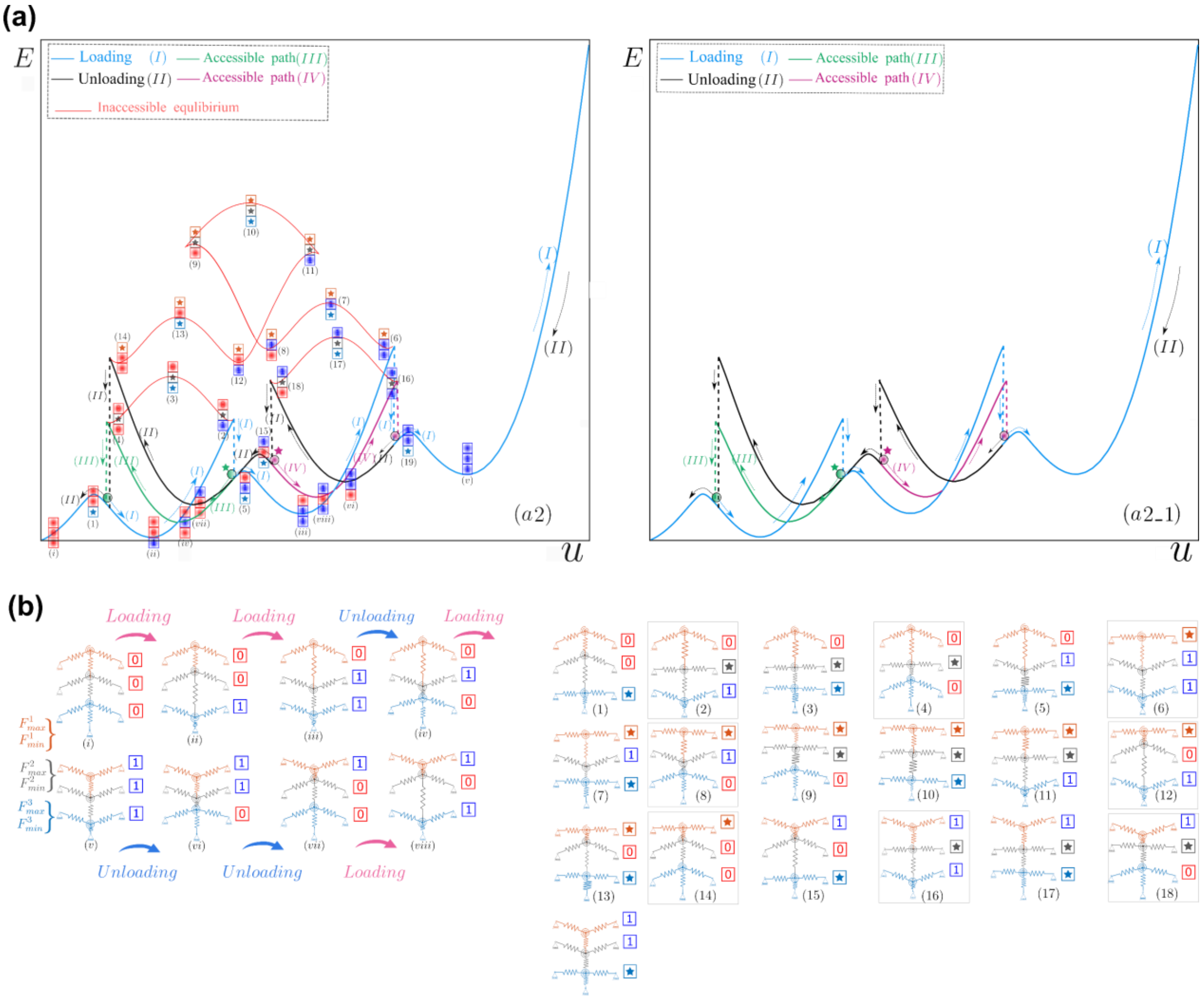

Figure S21. A chain comprises three bistable unit cells, characterized by $F^3_{max} < F^2_{max} < F^1_{max}, and\ F^1_{min} < F^2_{min} < F^3_{min}$, in which, the blue line (I), black line (II) demonstrate the loading and unloading paths, respectively, and the green (III), and purple (IV) lines illustrate the accessible paths in loading and unloading paths which capture two distinct stable configurations, and the dash lines show the energy drop off in snapping back. Moreover, the green and purple balls illustrate that the chain naturally is inclined to pass the local minima. **(a2)** energy-displacement of the chain in which the blurred red line (V) shows the inaccessible path. Zero (red square), and one (blue square) show the initial and second stable configurations, respectively, and the star sign illustrates the unstable configuration of the pertinent cell. Also, $(i) - (viii)$ show the stable configurations which are located in local minima of the energy-displacement curve, and $(1) - (19)$ depict the unstable configurations in the chain which are corresponding to the high level of energy in the curve. **(a2-1)** the displacement control energy-displacement curve of the chain. **(b)** schematic illustration of the stable and unstable configurations in which the way to capture all the stable configurations based on the orders of F_{max}, and F_{min}, without investigating the force-displacement curve, has been presented.

S2.1.4. Continuous energy-displacement curve

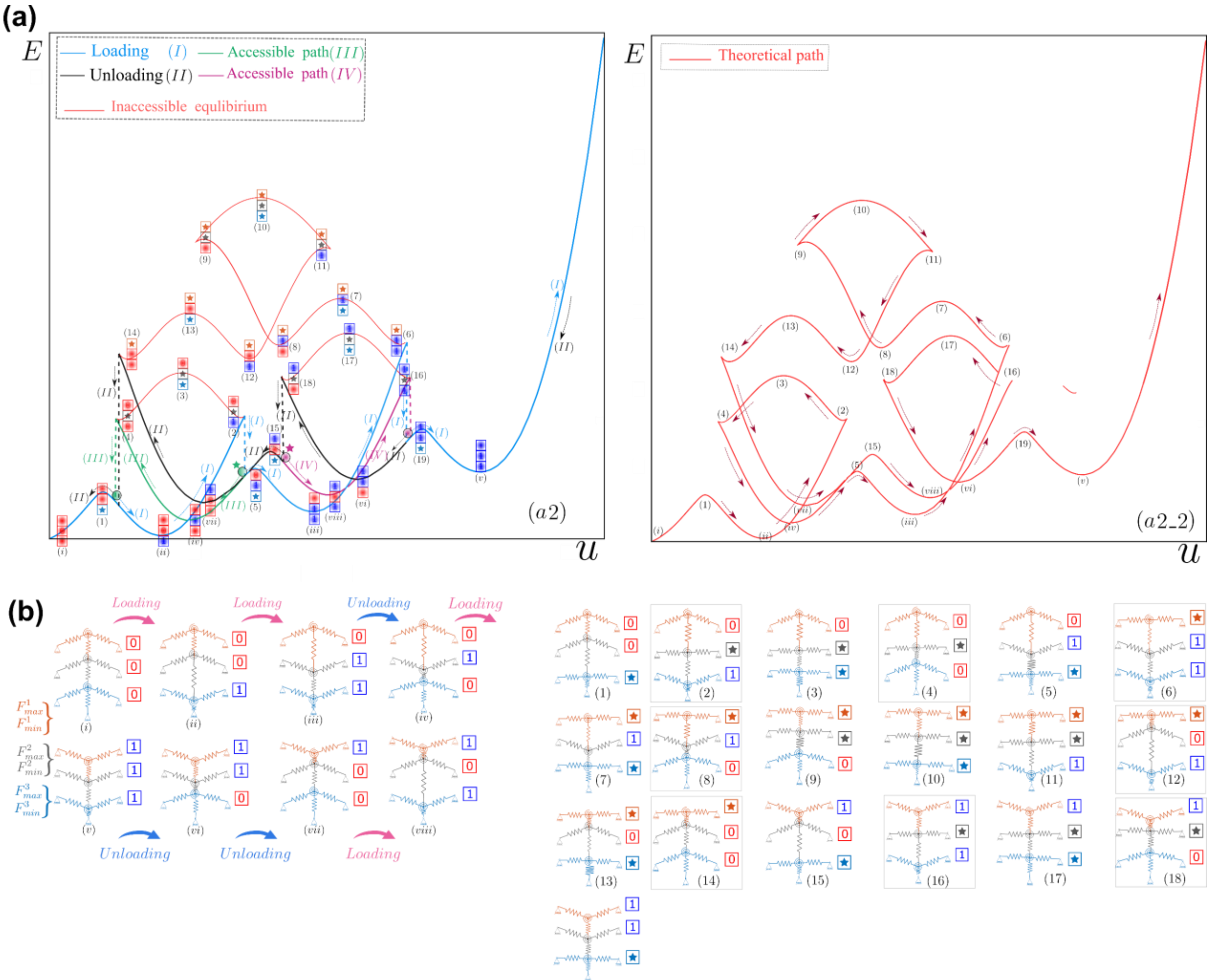

Figure S22. A chain comprises three bistable unit cells, characterized by $F_{max}^3 < F_{max}^2 < F_{max}^1$, *and* $F_{min}^1 < F_{min}^2 < F_{min}^3$, in which, the blue line (I), and black line (II) demonstrate the loading and unloading paths, respectively, and the green (III), and purple (IV) lines illustrate the accessible paths in loading and unloading paths which capture two distinct stable configurations, and the dash lines show the energy drop off in snapping back. Moreover, the green and purple balls illustrate that the chain naturally is inclined to pass the local minima. **(a2)** energy-displacement of the chain in which the blurred red line (V) shows the inaccessible path. Zero (red square), and one (blue square) show the initial and second stable configurations, respectively, and the star sign illustrates the unstable configuration of the pertinent cell. Also, $(i) - (viii)$ show the stable configurations which are located in local minima of the energy-displacement curve, and $(1) - (19)$ depict the unstable configurations in the chain which are corresponding to the high level of energy in the curve. **(a2-2)** the continuous energy-displacement curve of the chain in which the red arrows illustrate the direction of the path in loading. **(b)** schematic illustration of the stable and unstable configurations in which the way to capture all the stable configurations based on the orders of F_{max}, and F_{min}, without investigating the force-displacement curve, has been presented.

S2.1.5. Deformation sequence

All the stable configurations and corresponded sequences have been captured based on the $F^3_{max} < F^2_{max} < F^1_{max}, and\ F^1_{min} < F^2_{min} < F^3_{min}$.

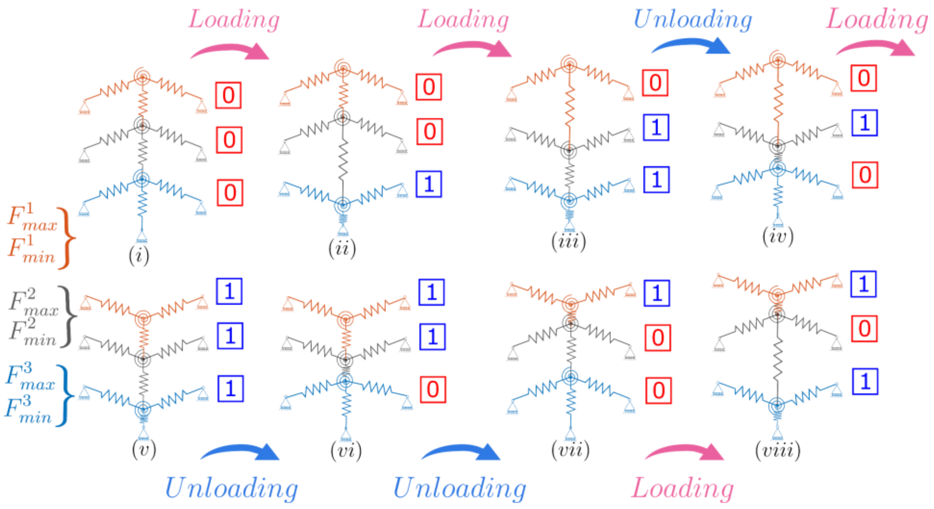

Figure S23. Schematic illustration of the stable configurations in which the way to capture all the stable configurations based on the orders of F_{max}, and F_{min}, without investigating the force-displacement curve, has been presented. In this case, the chain comprises three bistable unit cells, characterized by $F^3_{max} < F^2_{max} < F^1_{max}, and\ F^1_{min} < F^2_{min} < F^3_{min}$. Zero (red square), and one (blue square) show the initial and second stable configurations, and $(i) - (viii)$ are the distinct stable configurations, and the red and blue arrows demonstrate the forward controlled displacement (loading), and backward controlled displacement (unloading), respectively.

S2.2. Longest path and speed of reconfigurability

Now, we focus on finding the longest path and speed of reconfigurability in the chain. We found that the longest path is related to the leftmost/rightmost cases of the paths. Assume the chain is in (000) configuration. To achieve the (101) configuration the chain should pass six stable configurations, which are $(001), (011), (111), (110), (100)$, and eventually (101). Now, if the chain is located in the (111) configuration, it should go through $(110), (100), (000), (001), (011)$ to reach (010). We have extended our conclusion to the "n" number of bistable unit cells. when we have the same order of absolute positive and negative instability force, 2^n Configurations are possible. By applying a forward control displacement, cells go to the second stable configuration; whenever one cell goes to the second stable configuration, we count one loading and vice versa in unloading. The furthest configuration in terms of the number of loading and unloading required is the state of *(101 … 101)*, which needs $n(n+1)/2$ loading and unloading. Knowing the loading and unloading process's length helps us get important information from the material history just from the current configuration and length of the loading and unloading process. This number also is very important especially

when these materials are used as a memory. This length shows the speed of writing that is affected by memory capacity. This availability to get all configurations can be affected by order of maximum and minimum forces.

S2.3. Mathematical model

We systematically explore the number of configurations in a chain comprised of "n" cells. Then, we conceptually found how changing the governing conditions reduces the number of stable configurations. Moreover, what are the effective parameters? We found that the effective parameter is the arrangement of the maximum and minimum force of the cells. Here for a better explanation, the cells are arranged based on their maximum and minimum force for, i.e., the cell, which has a maximum instability force, and the lowest minimum force, is pertinent to 1. By this order, the cell with a minimum instability force and highest minimum force correspond to n, where n is the number of unit cells. In the following paragraph, the discrepancy will be defined, and the number of configurations for different discrepancies will be discussed.

Assuming that the maximum force is arranged by the defined order, the discrepancy is defined when the minimum force in the $(i+1)^{th}$cell becomes lower than the ith cell. Furthermore, we defined the number of discrepancies term by "c"; where the minimum force of the $(i+c)^{th}$ cell is lower than the minimum force of the ith cell. Also, "m" illustrates the number of cells in the second stable state.

S2.3.1. Without discrepancy (Ideal case)

According to the force-displacement curve of Figure 5(b1) in the main text, the number of discrepancies is zero, and therefore, the total number of stable configurations is 2^3, which means each cell has two options (initial stable and second stable configurations), and all the accessible configurations are unique. Therefore, the total number of accessible configurations is equal to 2^n. More accurately, the number of the configurations with "m" cells in the second stable state is the selection of m from, $\binom{n}{m}$. Obviously, this behavior follows the Pascal triangle, illustrated in Figure 5(b1) in the main text. For instance, as illustrated in Figure 5(b1), assume the chain of Figure 5(a1) with three cells; the number of configurations with zero, one, two, and three cells in the second stable configuration is $\binom{3}{0}$, $\binom{3}{1}$, $\binom{3}{2}$, and $\binom{3}{3}$, respectively. Also, the total number of configurations is 2^3.

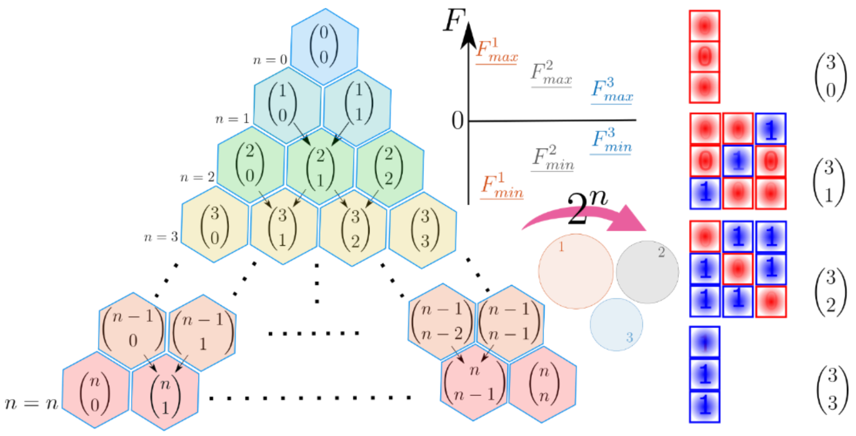

Figure S24. A mathematical model based on the Pascal triangle along with the set theory to capture the number of stable configurations in a chain without any discrepancy, where the initial and second stable configurations have been shown by zero (red square), and one (blue square). The ideal case without discrepancy has been defined based on the $F^3_{max} < F^2_{max} < F^1_{max}, and\ F^1_{min} < F^2_{min} < F^3_{min}$; in this case, the cells are similar to independent sets, and therefore, all the stable configurations are accessible, which is equal to 2^n. Also, $\binom{n}{m}$ shows the number of stable configurations in a chain comprising n cells in which m cells are in second stable configurations.

S2.3.2. One discrepancy

Clearly, by applying a discrepancy in the chain cells, the number of configurations changes. This case has been shown in Figure 5(b2), in which one discrepancy has been applied to the chain's cell. Two possibilities occur, demonstrated by case I and case II. In case I, the minimum force in cell II is less than cell III, while the maximum force in cell II is higher than in cell III. In this case, the number of stable configurations with $m = 0, 1, 2, 3$ become $1, 2, 2, 1$, respectively; so, six configurations are totally accessible. Compared to the case without discrepancy, the $(010), (110)$ configurations become inaccessible. In case II, the minimum force in cell II is higher than in cell I, while the maximum force in cell II is lower than in cell I. In this case, the number of stable configurations with $m = 0, 1, 2, 3$ become $1, 2, 2, 1$, respectively; so, six configurations are accessible. Compared to the case without discrepancy, the $(100), (101)$ configurations become inaccessible.

Assume a chain comprises "n" cells, the number of configurations that has a "m" cells in the second stable state is equal to the difference of the row "n^{th}", and "$(n-2)^{th}$" row of the Pascal triangle for a specific "m". Therefore, the leftmost and rightmost of the "n^{th}" row of the Pascal triangle remain unchanged; these values are related to $m = 0$, and $m = n$. Otherwise, the number of the configurations for an arbitrary "m" is $\binom{n}{m} - \binom{n-2}{m-1}$. Therefore, by some manipulation, it can be easily shown that the total number of configurations is $2^n - 2^{n-2}$. Also, the difference in the summation of the "n^{th}" row values and "$(n-2)$" row values show the

same result. For example, consider cases I and II, the number of configurations for $m = 0$, and $m = 3$ are equal to $\binom{3}{0}$, and $\binom{3}{3}$, while for $m = 1$, and $m = 2$, the number of configurations is $\binom{3}{1} - \binom{1}{0}$, and $\binom{3}{2} - \binom{1}{1}$, respectively. Therefore, the total number of the configuration is also equal to the difference of the summation on third-row values and 1st-row values of the Pascal triangle; In other words, it is equal to $(2^3 - 2^1)$.

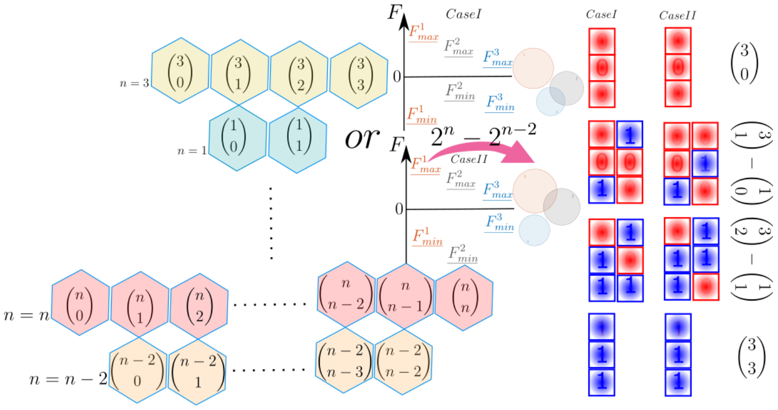

Figure S25. a mathematical model based on the Pascal triangle along with the set theory to capture the number of stable configurations in a chain with one discrepancy, where the initial and second stable configurations have been shown by zero (red square), and one (blue square). Case I, and case II are characterized by $\{F_{max}^3 < F_{max}^2 < F_{max}^1, and\ F_{min}^1 < F_{min}^3 < F_{min}^2\}$, and $\{F_{max}^3 < F_{max}^2 < F_{max}^1, and\ F_{min}^2 < F_{min}^1 < F_{min}^3\}$, respectively. In this case, the cells are similar to dependent sets and therefore, all the stable configurations are not accessible. Therefore, the difference between the rows "n^{th}", and "$(n-2)^{th}$" of the Pascal triangle for a specific "m" is equal to the number of stable configurations in which the "m" cells are in a second stable configuration, which is $\binom{n}{m} - \binom{n-2}{m-1}$, and the total number of accessible stable configurations is $2^n - 2^{n-2}$.

S2.4. Verification of mathematical theory

The results of Figure S24 and Figure S25 have been verified by studying the actual paths.

S2.4.1. Force-displacement and energy-displacement for Case I

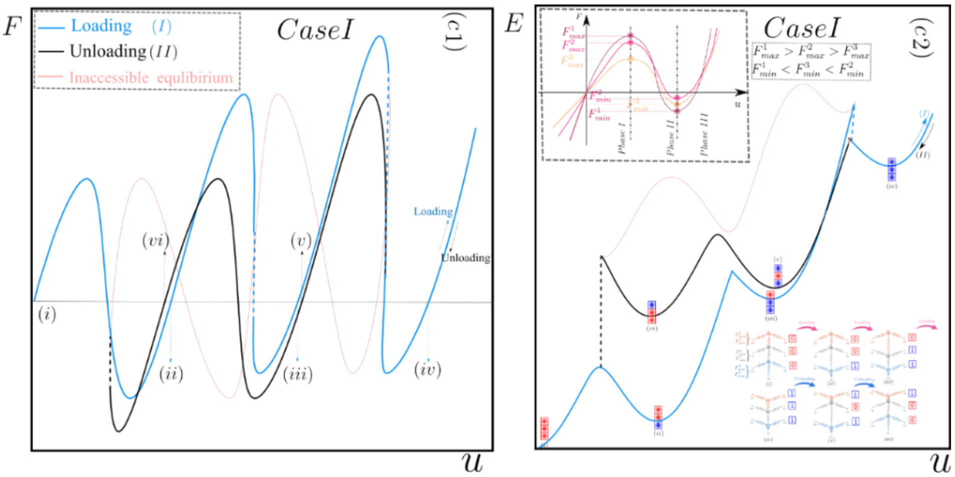

Figure S26. a chain comprises three bistable unit cells, characterized by $F^3_{max} < F^2_{max} < F^1_{max}, and\ F^1_{min} < F^3_{min} < F^2_{min}$, in which, **(c1),** and **(c2)** are the corresponding force-displacement and energy-displacement curves. Where the blue line (I) and the black line (II) demonstrate the loading and unloading paths, respectively, the blurred red line shows the inaccessible path, and the dash lines show the energy drop-off in snapping back. Zero (red square), and one (blue square) show the initial and second stable configurations, respectively. Also, $(i) - (vi)$ show the stable configurations which are located in the local minima of the energy-displacement curve. Moreover, schematic illustration of the stable configurations in which the way to capture all the stable configurations based on the orders of F_{max}, and F_{min}, without investigating the force-displacement curve, has been presented. The results are entirely in agreement with mathematical theory.

S2.4.2. Force-displacement and energy-displacement for Case II

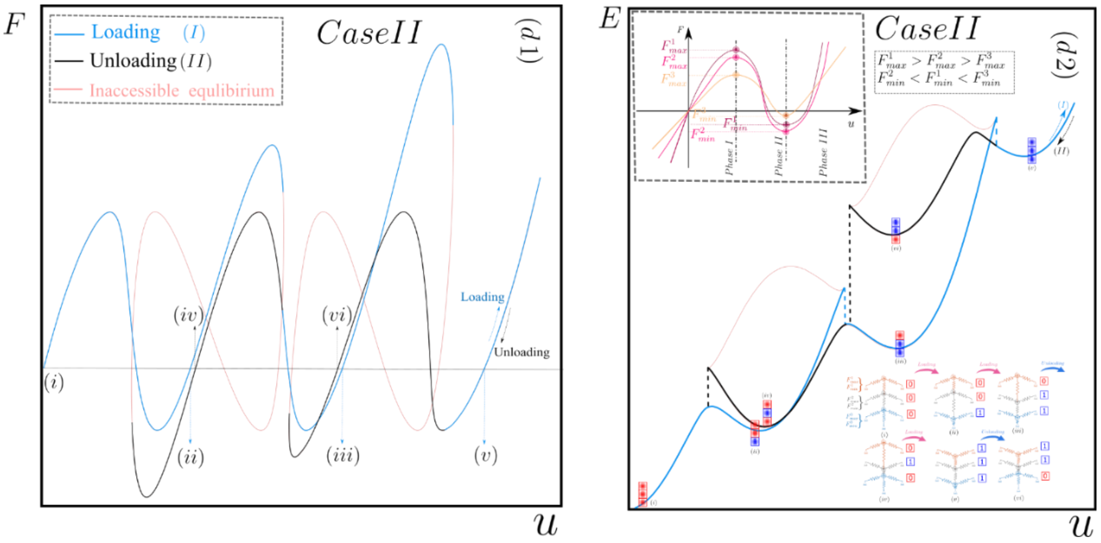

Figure S27. a chain comprises three bistable unit cells, characterized by $F^3_{max} < F^2_{max} < F^1_{max}, and\ F^2_{min} < F^1_{min} < F^3_{min}$, in which, **(d1),** and **(d2)** are the corresponding force-displacement and energy-displacement curves. Where, the blue line (I), and black line (II) demonstrate the loading and unloading paths, respectively, the blurred red line shows the inaccessible path, and the dash lines show the energy drop-off in snapping back. Zero (red

square), and one (blue square) show the initial and second stable configurations, respectively. Also, $(i) - (vi)$ show the stable configurations which are located in local minima of the energy-displacement curve. Moreover, schematic illustration of the stable configurations in which the way to capture all the stable configurations based on the orders of F_{max}, and F_{min}, without investigating the force-displacement curve, has been presented. The results are entirely in agreement with mathematical theory.

S2.5. Other possibilities of a chain composing three cells in series

In the following cases, the chains comprise three unit cells with more than one discrepancy.

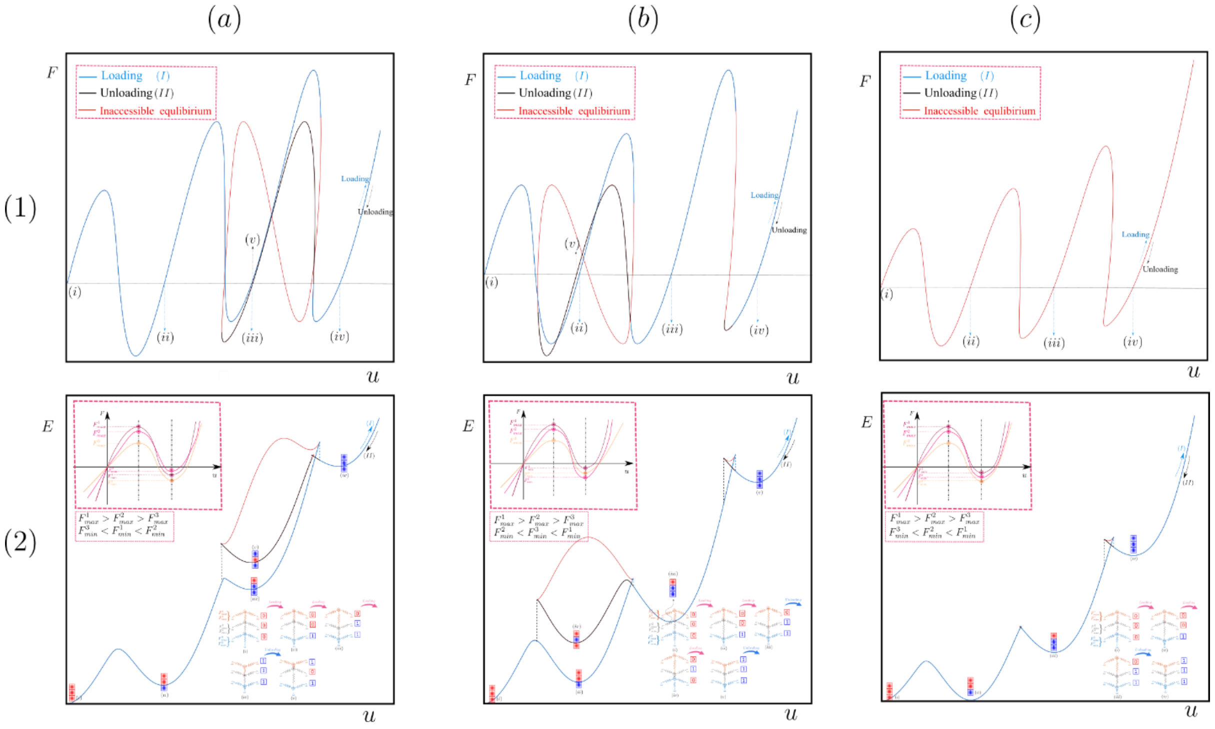

Figure S28. a chain comprises three bistable unit cells, characterized by **(a)** $F_{max}^3 < F_{max}^2 < F_{max}^1$, *and* $F_{min}^3 < F_{min}^1 < F_{min}^2$, **(b)** $F_{max}^3 < F_{max}^2 < F_{max}^1$, *and* $F_{min}^2 < F_{min}^3 < F_{min}^1$, **(c)** $F_{max}^3 < F_{max}^2 < F_{max}^1$, *and* $F_{min}^3 < F_{min}^2 < F_{min}^1$, in which, rows **(1),** and **(2),** are the corresponding force-displacement and energy-displacement curves, respectively, Where, the blue line (I), black line (II) demonstrate the loading and unloading paths, respectively, the blurred red line shows the inaccessible path, and the dash lines show the energy drop off in snapping back. Zero (red square), and one (blue square) show the initial and second stable configurations, respectively. Also, $(i) - (v)$ show the stable configurations which are located in the local minima of the energy-displacement curve. Moreover, schematic illustration of the stable configurations in which the way to capture all the stable configurations based on the orders of F_{max}, and F_{min}, without investigating the force-displacement curve, has been presented. Cases (a), and (b) are corresponding to two discrepancies, leading to five stable configurations, and case (c) is pertinent to three discrepancies, leading to four stable configurations.

S2.6. Chain configurations in high energy levels in a continuous path

As shown in Figure S29, the chain configuration in the high energy level at an inaccessible path shows *external stability* and *internal instability.* These definitions have been introduced in the following figure.

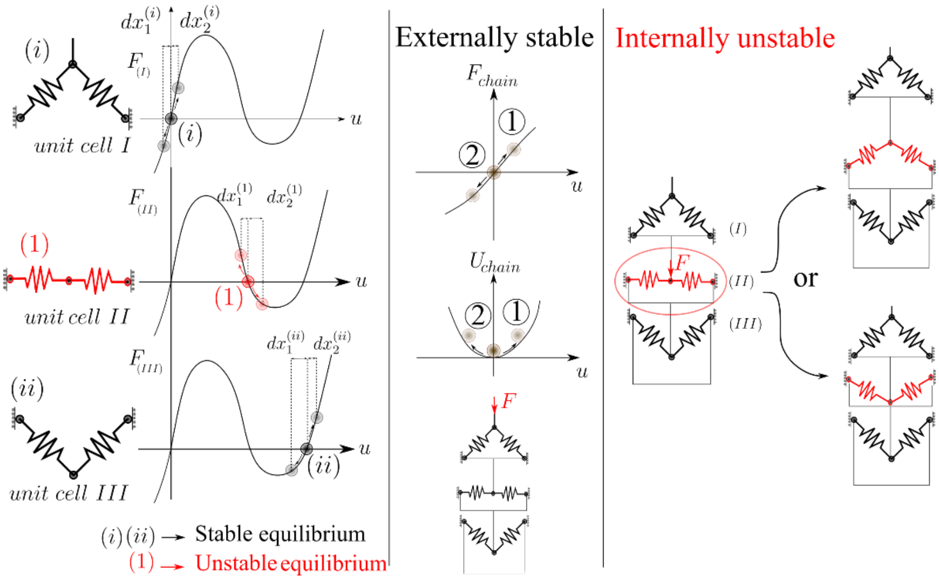

Figure S29. Studying the externally stable and internally unstable positions **(a)** in a chain comprised of three bistable unit cells, in which cells I, II, and III are in initial stable (i), unstable (1), and second stable (ii) configurations, respectively. The corresponded points in the force-displacement curve of the cells have been shown by (i), (1), and (ii). **(b)** schematic force-displacement and energy-displacement curves of the chain, which illustrate that the chain is externally stable. However, **(c)** cell II is in an unstable phase, and it naturally goes to initial or second stable states, which illustrates that the chain is internally unstable.

S2.7. Four cells in series

As shown in Figure S30, the chain comprises four bistable unit cells with different discrepancies.

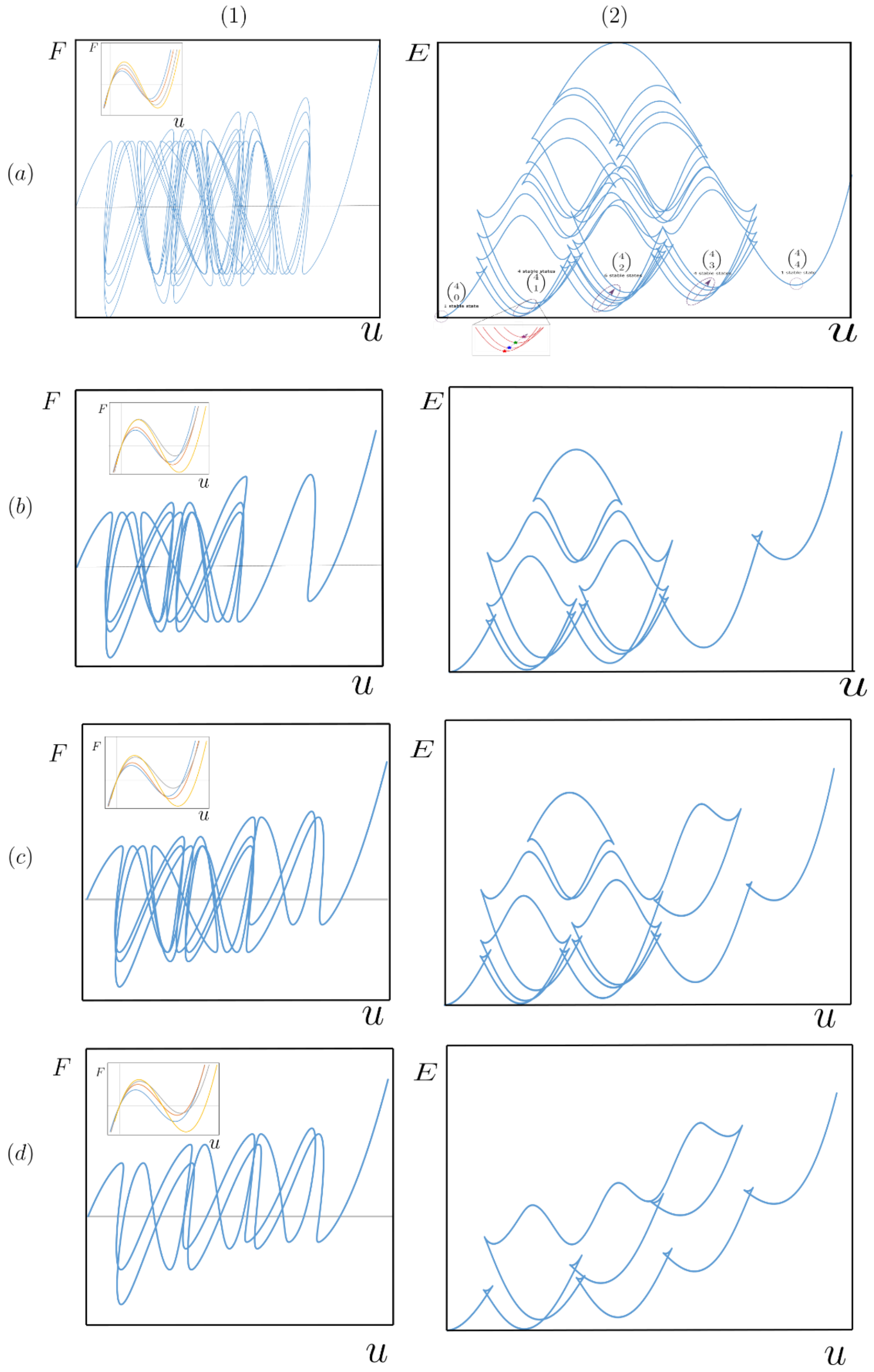

Figure S30. A chain comprises four bistable unit cells, characterized by the force-displacement curve of the cells. Columns **(1),** and **(2)** are the corresponding force-displacement and energy-displacement curves, respectively, where the blue line demonstrates the continuous paths.

S2.8. Generalization of the mathematical theory

Here, the number of stable configurations for a chain comprising three unit cells, has been studied. As shown in Figure S31, the stable configurations for different discrepancies have been discussed. Interestingly, the method to capture the inaccessible configurations has been developed.

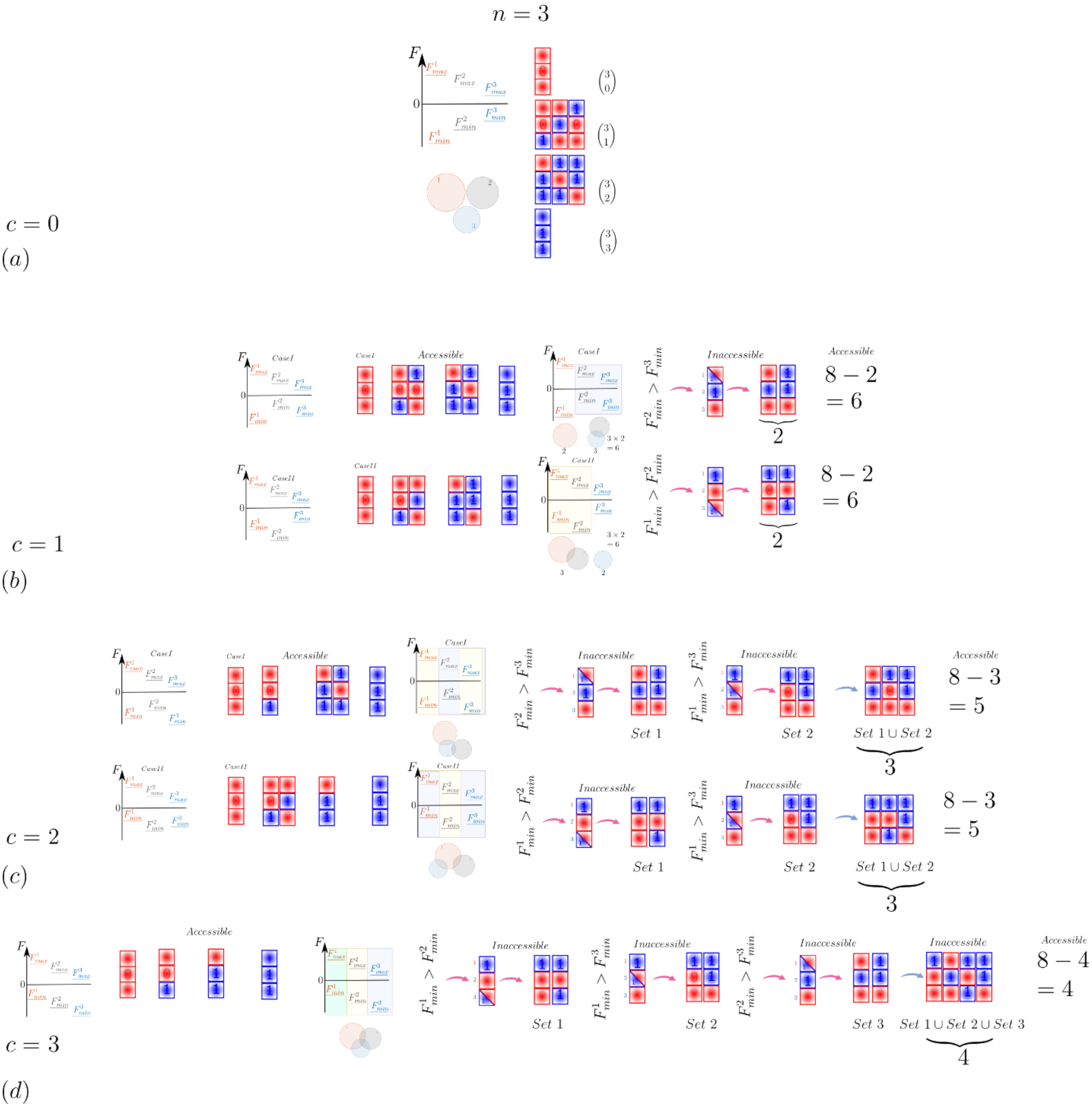

Figure S31. Investigating the accessible and inaccessible stable configurations in a chain that comprises three bistable unit cells with four different discrepancies; c=0, …, 3. The initial and second stable configurations have been shown by zero (red square), and one (blue square). **(a)** the ideal case without discrepancy has been shown based on the $F^3_{max} < F^2_{max} < F^1_{max}, and\ F^1_{min} < F^2_{min} < F^3_{min}$; in this case, the cells are similar to independent sets and therefore, all the stable configurations are accessible, which is equal to 2^3. Also, $\binom{n}{m}$ shows the number of stable configurations in a chain comprising n cells in which m cells are in second stable configurations. **(b)** cases I, and II with one discrepancy have been illustrated based on the $F^3_{max} < F^2_{max} < F^1_{max}, and\ F^1_{min} < F^3_{min} < F^2_{min}$

(case I), and $F_{max}^3 < F_{max}^2 < F_{max}^1, and\ F_{min}^2 < F_{min}^1 < F_{min}^3$ (case II). In case I, $F_{min}^3 < F_{min}^2$ is a discrepancy and therefore, the inaccessible configurations are (010), (110). In case II, $F_{min}^2 < F_{min}^1$ is a discrepancy and thereby, the inaccessible configurations are (100), and (101). Consequently, in both cases, six stable configurations are accessible**.** **(c)** the cases I and II with two discrepancies have been illustrated based on the $F_{max}^3 < F_{max}^2 < F_{max}^1, and\ F_{min}^3 < F_{min}^1 < F_{min}^2$ (case I), and $F_{max}^3 < F_{max}^2 < F_{max}^1, and\ F_{min}^2 < F_{min}^3 < F_{min}^1$ (case II). In case I, $F_{min}^3 < F_{min}^2, and\ F_{min}^3 < F_{min}^1$ are the discrepancies and therefore, the inaccessible configurations are (010), (100), and (110). In case II, $F_{min}^2 < F_{min}^1$, and $F_{min}^3 < F_{min}^1$ are the discrepancies and thereby, the inaccessible configurations are (100), (101), and (110). Consequently, in both cases, five stable configurations are accessible. **(d)** one case with three discrepancies has been illustrated based on the $F_{max}^3 < F_{max}^2 < F_{max}^1, and\ F_{min}^3 < F_{min}^2 < F_{min}^1$, In which I, $F_{min}^2 < F_{min}^1$, $F_{min}^3 < F_{min}^1$, $and\ F_{min}^3 < F_{min}^2$ are the discrepancies and thereby, the inaccessible configurations are (100), (010), (101), and (110). Consequently, in this case, four stable configurations are accessible. Note that the intersection of the sets is directly related to the discrepancies in the chain.

Here, the number of stable configurations for a chain comprising four unit cells, has been studied. As shown in Figure S32, the accessible, stable configurations for different discrepancies have been discussed.

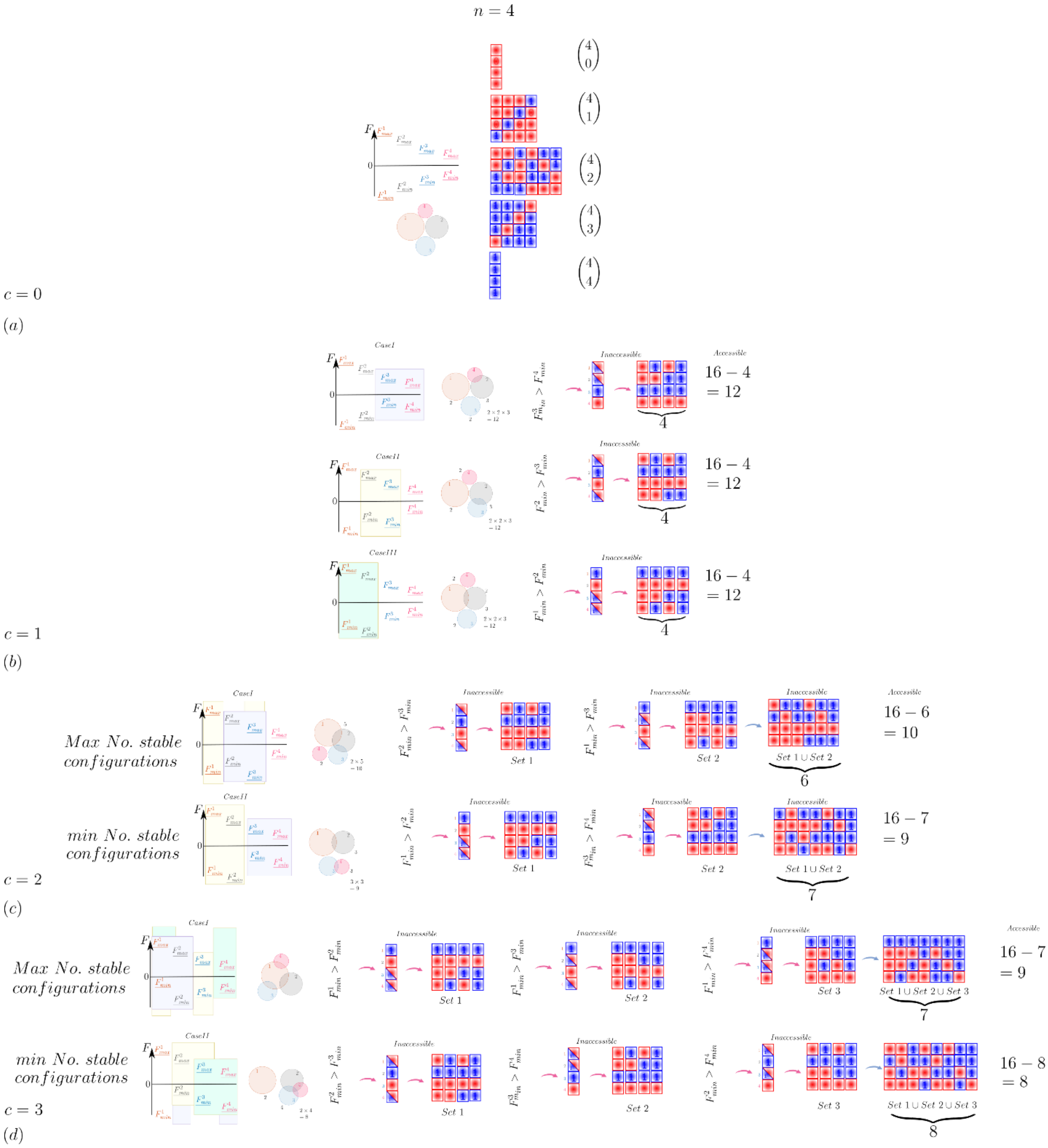

Figure S32. Investigating the accessible and inaccessible stable configurations and the number of stable configurations in each discrepancy in a chain that comprises four bistable unit cells with four different discrepancies; c=0, …, 3. The initial, and second stable configurations have been shown by zero (red square), and one (blue square). **(a)** The ideal case without discrepancy has been shown based on the $F^4_{max} < F^3_{max} < F^2_{max} < F^1_{max}, and\ F^1_{min} < F^2_{min} < F^3_{min} < F^4_{min}$; in this case, the cells are similar to independent sets and therefore, all the stable configurations are accessible, which is equal to 2^4. Also, $\binom{n}{m}$ shows the number of stable configurations in a chain comprising n cells in which m cells are in second stable configurations. **(b)** cases I, II, and III with one discrepancy have been illustrated based on the $F^4_{max} < F^3_{max} < F^2_{max} < F^1_{max}, and\ F^1_{min} < F^2_{min} < F^4_{min} < F^3_{min}$ (case I), $F^4_{max} < F^3_{max} < F^2_{max} < F^1_{max}, and\ F^1_{min} < F^3_{min} < F^2_{min} < F^4_{min}$ (case II), and $F^4_{max} < F^3_{max} < F^2_{max} < F^1_{max}, and\ F^2_{min} < F^1_{min} < F^3_{min} < F^4_{min}$. In case I, $F^4_{min} < F^3_{min}$ is a discrepancy and therefore, the inaccessible configurations are (0010), (1010), (0110), and (1110). In case II, $F^3_{min} < F^2_{min}$ is a discrepancy and thereby, the

inaccessible configurations are (0100), (1100), (0101), and (1101). In case III, $F_{min}^2 < F_{min}^1$ is a discrepancy and thereby, the inaccessible configurations are (1000), (1001), (1010), and (1011). Consequently, in all cases, 12 stable configurations are accessible. **(c)** the cases I and II with two discrepancies have been illustrated based on the $F_{max}^4 < F_{max}^3 < F_{max}^2 < F_{max}^1, and\ F_{min}^3 < F_{min}^1 < F_{min}^2 < F_{min}^4$ (case I), and $F_{max}^4 < F_{max}^3 < F_{max}^2 < F_{max}^1, and\ F_{min}^2 < F_{min}^1 < F_{min}^4 < F_{min}^3$ (case II). In case I, $F_{min}^3 < F_{min}^2$, and $F_{min}^3 < F_{min}^1$ are the discrepancies and therefore, the inaccessible configurations are (0100), (1000), (1100), (0101), (1001), and (1101). In case II, $F_{min}^2 < F_{min}^1$, and $F_{min}^4 < F_{min}^3$ are the discrepancies and thereby, the inaccessible configurations are (1000), (0010), (1001), (1010), (0110), (1011), and (1110). Consequently, 10 stable configurations in case I are accessible while the number of stable configurations in case II is equal to nine. **(d)** the cases I and II with three discrepancies have been illustrated based on the $F_{max}^4 < F_{max}^3 < F_{max}^2 < F_{max}^1, and\ F_{min}^2 < F_{min}^3 < F_{min}^4 < F_{min}^1$ (case I), and $F_{max}^4 < F_{max}^3 < F_{max}^2 < F_{max}^1, and\ F_{min}^1 < F_{min}^4 < F_{min}^3 < F_{min}^2$ (case II). In case I, $F_{min}^2 < F_{min}^1$, $F_{min}^3 < F_{min}^1$ and $F_{min}^4 < F_{min}^1$ are the discrepancies and therefore, the inaccessible configurations are (1000), (1001), (1010), (1100), (1011), (1101), and (1110). In case II, $F_{min}^3 < F_{min}^2$, $F_{min}^4 < F_{min}^3$, and $F_{min}^4 < F_{min}^2$ are the discrepancies and thereby, the inaccessible configurations are (0100), (0010), (1100), (0101), (1010), (0110), (1101), and (1110). Consequently, nine stable configurations in case I are accessible while the number of stable configurations in case II is equal to eight. According to (c), and (d), not only the number of discrepancies is important but also the arrangement of the discrepancies has a key role in determining the number of stable configurations. Note that each set is a representative of the corresponded cell and the intersection of the sets is directly related to the discrepancies in the chain. Therefore, the number of accessible stable configurations has been obtained by using the defined sets.

Generally, the total number of arrangements of the cells is $\binom{n}{2}$. A summary of the results has been shown in the following table:

Table S2. Number of stable configurations for a chain, comprised of n cell with c discrepancies.

n (number of cells)	c (number of discrepancies)	Number of stable configurations
2	0	4
	1	3
3	0	8
	1	6
	2	5
	3	4
4	0	16
	1	12
	2	9,10*
	3	8,9*

	4	7
	5	6
	6	5
5	0	32
	1	24
	2	18,20*
	3	-**
	4	12
	5	11
	6	10
	7	9
	8	8
	9	7
	10	6

* Maximum number of stable configurations

** Theory cannot capture it

By considering the aforementioned definition and presented table, modified formulations have been tabulated below to capture the minimum number of stable configurations for the arbitrary number of discrepancies.

Table S3. Minimum number of stable configurations for a chain, comprised of n cells with c discrepancies.

c	Minimum number of stable configurations	Required conditions
0	$\sum_{m=0}^{n}\binom{n}{m}$	
1	$\sum_{m=0}^{n}\binom{n}{m}-\sum_{m=0}^{n-2}\binom{n-2}{m}$	$n\geq 2$
2	$\sum_{m=0}^{n}\binom{n}{m}-2\sum_{m=0}^{n-2}\binom{n-2}{m}+\sum_{m=0}^{n-4}\binom{n-4}{m}$	$n\geq 4$
3	$\sum_{m=0}^{n}\binom{n}{m}-3\sum_{m=0}^{n-2}\binom{n-2}{m}+3\sum_{m=0}^{n-4}\binom{n-4}{m}-\sum_{m=0}^{n-6}\binom{n-6}{m}$	$n\geq 6$
4	$\sum_{m=0}^{n}\binom{n}{m}-4\sum_{m=0}^{n-2}\binom{n-2}{m}+6\sum_{m=0}^{n-4}\binom{n-4}{m}-4\sum_{m=0}^{n-6}\binom{n-6}{m}+\sum_{m=0}^{n-8}\binom{n-8}{m}$	$n\geq 8$

As shown in the following table the coefficient of the terms follows Pascal's triangle.

Table S4. The coefficient of the terms.

				1				
			1		1			
		1		2		1		
	1		3		3		1	
1		4		6		4		1
$\iddots$								$\ddots$

It can be easily shown that

$$\psi(n,c)_{m=m_0} = \begin{cases} 1 & m_0 = 0, n \\ \sum_{j=0}^{c} (-1)^j \binom{c}{j} \binom{n-2j}{m_0-j} & m_0 \neq 0, n \end{cases} \qquad n-2j \geq 0, \tag{1}$$

where in Eq.(1), $\psi(n,c)_{m=m_0}$ is the number of minimum stable configurations that have "m_0" cells in the second stable state. Also,

$$\varphi(n,m,c) = \sum_{j=0}^{c} \sum_{m=0}^{n-2j} (-1)^j \binom{c}{j} \binom{n-2j}{m} \qquad n-2j \geq 0. \tag{2}$$

Where in Eq.(1), $\varphi(n,m,c)$ is the minimum number of stable configurations. Finally, the results and limitations of the theory have been summarized in the following table.

Table S5. minimum number of stable configurations for a chain, comprising n cell with c discrepancies.

	***c* (discrepancies)**	**Minimum number of stable configurations**
n	0	$\sum_{m=0}^{n}\binom{n}{m}$
	1	$\sum_{j=0}^{1}\sum_{m=0}^{n-2j}(-1)^{j}\binom{1}{j}\binom{n-2j}{m}$
$[n/2]+1$	2	$\sum_{j=0}^{2}\sum_{m=0}^{n-2j}(-1)^{j}\binom{2}{j}\binom{n-2j}{m}$
	3	$\sum_{j=0}^{3}\sum_{m=0}^{n-2j}(-1)^{j}\binom{3}{j}\binom{n-2j}{m}$
$\frac{n(n-1)}{2}+1$	⋮	⋮
	It has not been captured	
$\lambda(n)=\begin{cases} n^2/4 & n=2k \\ (n^2-1)/4 & n=2k+1 \end{cases}$	$\frac{n(n-1)}{2}-\lambda(n)$	$(n+1)+\lambda(n)$
	⋮	⋮
	$\frac{n(n-1)}{2}-1$	$(n+2)$
	$\frac{n(n-1)}{2}$	$(n+1)$

S3. Multistability in tension and compression, and wave-like behavior in multistable materials

S3.1. Buckling load of slender beam

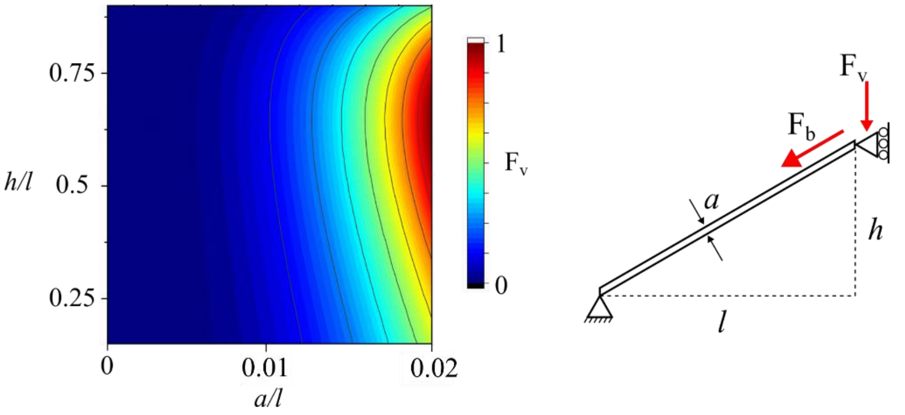

Figure S33. Vertical buckling load of an inclined beam. Results are normalized with highest buckling load.

By assuming buckling is happening in small deformations (a/l <<1) and Euler-Bernoulli beam formulation is valid. The beam cross-section is rectangular with "a" as thickness and "w" as width. Then, We have:

$$F_{cr} = F_b = \frac{EI\Pi}{L^2},$$

$$I = \frac{1}{12}a^3 w,$$

$$L = \sqrt{h^2 + l^2},$$

$$F_v = \frac{F_b}{\sin\theta} = F_b \frac{h}{\sqrt{h^2 + l^2}},$$

$$F_v = \frac{Ew\Pi}{12}\left(\frac{h}{\left(\sqrt{h^2 + l^2}\right)^3}\right)a^3$$

Results illustrate that although increasing height can help locate the beam in a more vertical position, it increases the length of the beam and can reduce buckling load. As it has been shown, there is an optimum point for height ratio.

S3.2. Multistability in tension

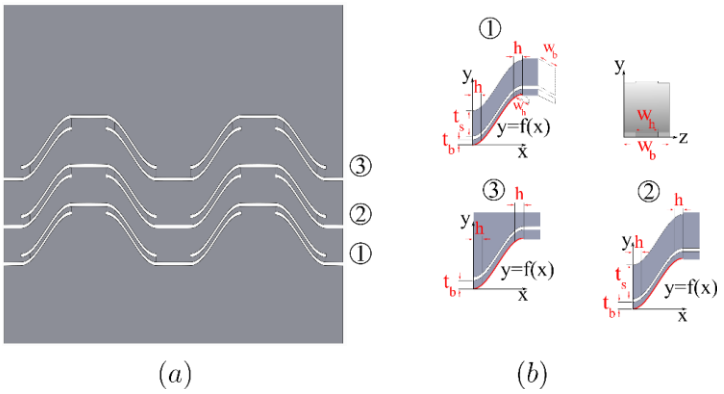

Figure S34. Multistability in tension. **(a)** a chain comprises three layers of sinusoidal beams. **(b)** nomenclature of the geometrical parameters of the cells.

Table S6. Assigning the geometrical parameters of the sinusoidal beams.

layer	function	h	t_b	t_s	W_h	W_b
1	$y = 9\left\{1 + sin\left(\frac{\pi x}{9} - \frac{\pi}{2}\right)\right\}$	4.2mm	1.8mm	9mm	15mm	30mm
2	$y = 9\left\{1 + sin\left(\frac{\pi x}{9} - \frac{\pi}{2}\right)\right\}$	4.2mm	2.4mm	12mm	15mm	30mm
3	$y = 9\left\{1 + sin\left(\frac{\pi x}{9} - \frac{\pi}{2}\right)\right\}$	4.2mm	3mm	–	15mm	30mm

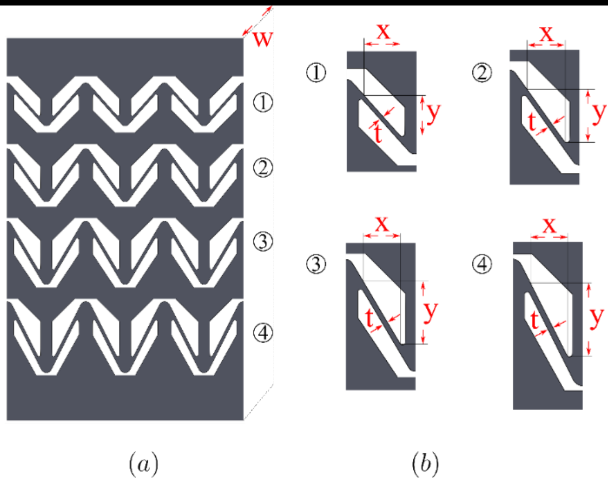

Figure S35. Multistability in tension. **(a)** a chain comprises three layers of inclined beams. **(b)** nomenclature of the geometrical parameters of the cells.

Table S7. Assigning the geometrical parameters of the inclined beams of a chain that has no discrepancy.

layer	x	y	t	w
**1**	8.2mm	8.2mm	1mm	30mm
**2**	8.2mm	10mm	1.1mm	30mm
**3**	8.2mm	12mm	1.2mm	30mm
**4**	8.2mm	13.7mm	1.3mm	30mm

Table S8. Assigning the geometrical parameters of the inclined beams of a chain which has a discrepancy.

layer	x	y	t	w
**1**	8.2mm	8.2mm	1mm	30mm
**2**	8.2mm	10mm	1.4mm	30mm
**3**	8.2mm	12mm	1.2mm	30mm
**4**	8.2mm	13.7mm	1.3mm	30mm

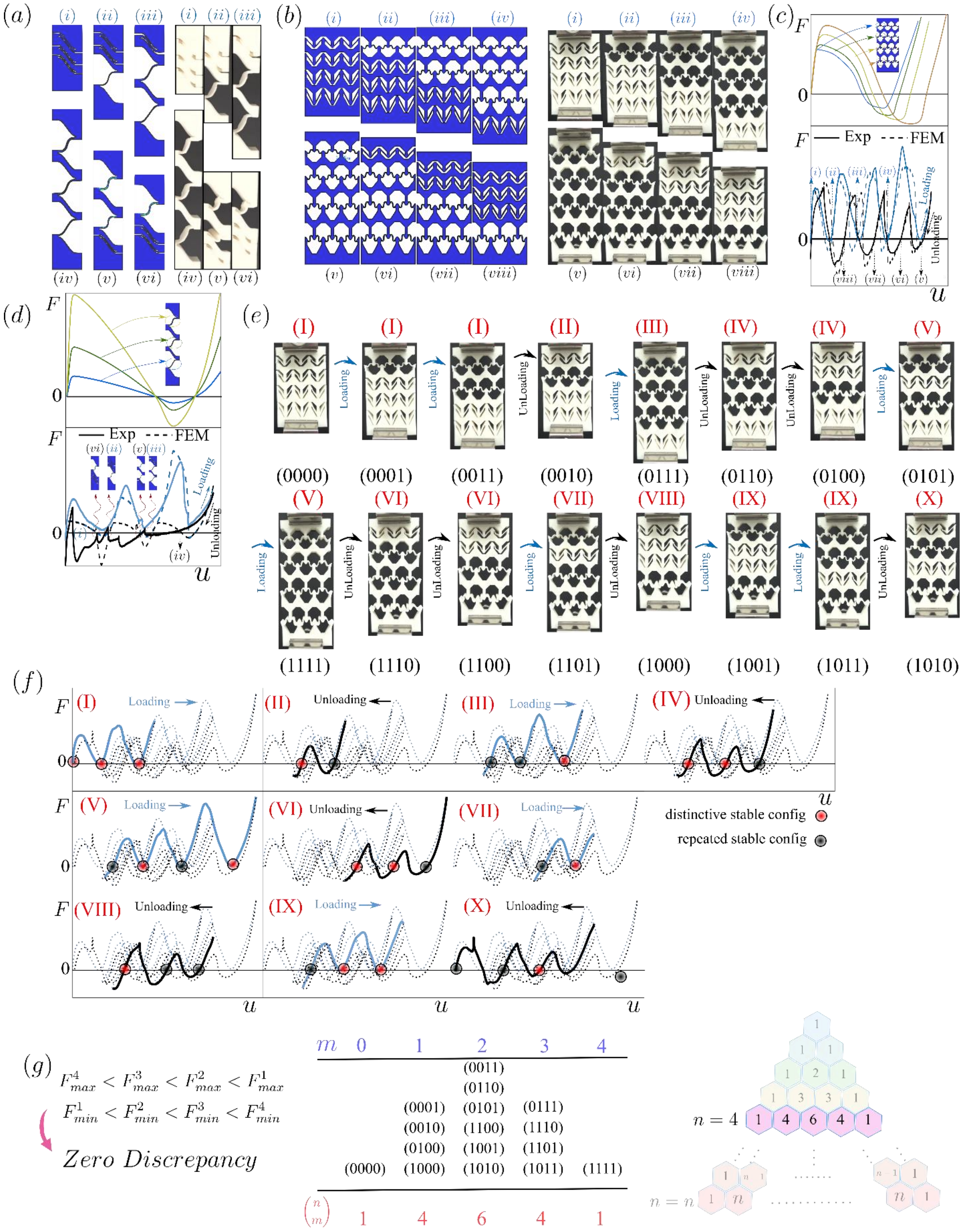

Figure S36. Experimental study of deformation sequence in multistable chains without discrepancy. **(a)** FEM and experimental results of a chain that comprises three bistable sinusoidal beams, characterized by $F^3_{max} < F^2_{max} < F^1_{max}, and\ F^1_{min} < F^2_{min} < F^3_{min}$. The accessible stable configurations in loading and unloading paths have been illustrated by $(i) - (vi)$. Generally, the number of accessible stable configurations in loading and unloading paths is equal to $2n$, where n is the number of cells. **(b)** FEM and experimental results of a chain that comprises four bistable inclined beams, characterized by $F^4_{max} < F^3_{max} < F^2_{max} < F^1_{max}, and\ F^1_{min} < F^2_{min} < F^3_{min} < F^4_{min}$.

Accessible stable configurations in loading and unloading paths have been demonstrated by $(i) - (viii)$. **(c)** Force-displacement curves of the cells and the chain corresponded to (b), respectively, where the solid line and dashed line illustrate the experimental and FEM results, respectively. Also, the blue and black lines demonstrate the loading and unloading paths. As shown, the results are in good agreement and the eight stable configurations $((i) - (viii))$ have been shown. **(d)** Force-displacement curves of the cells and the chain corresponding to (a), respectively, where the solid line and dashed line illustrate the experimental and FEM results, respectively. Also, the blue, and black lines demonstrate the loading and unloading paths. As shown, the results are in good agreement and the six stable configurations $((i) - (vi))$ have been shown. **(e)** 16 stable configurations of the chain and the way to achieve the configurations according to F_{max}, and F_{min} arrangement have been demonstrated. Also, the capital Greek letters illustrate the number that the direction of the applied displacement changes to capture the configurations. **(f)** deformation path in a controlled displacement experiment in which the blue and black dashed lines represent the experimental loading and unloading paths, respectively. The solid lines illustrate a part of the path to capture the stable configurations, where the red sign depicts the distinct stable configurations while the black sign shows the duplicated stable configurations. **(g)** verification of the results by mathematical theory, in which all the stable configurations have been captured by both theory and experiments.

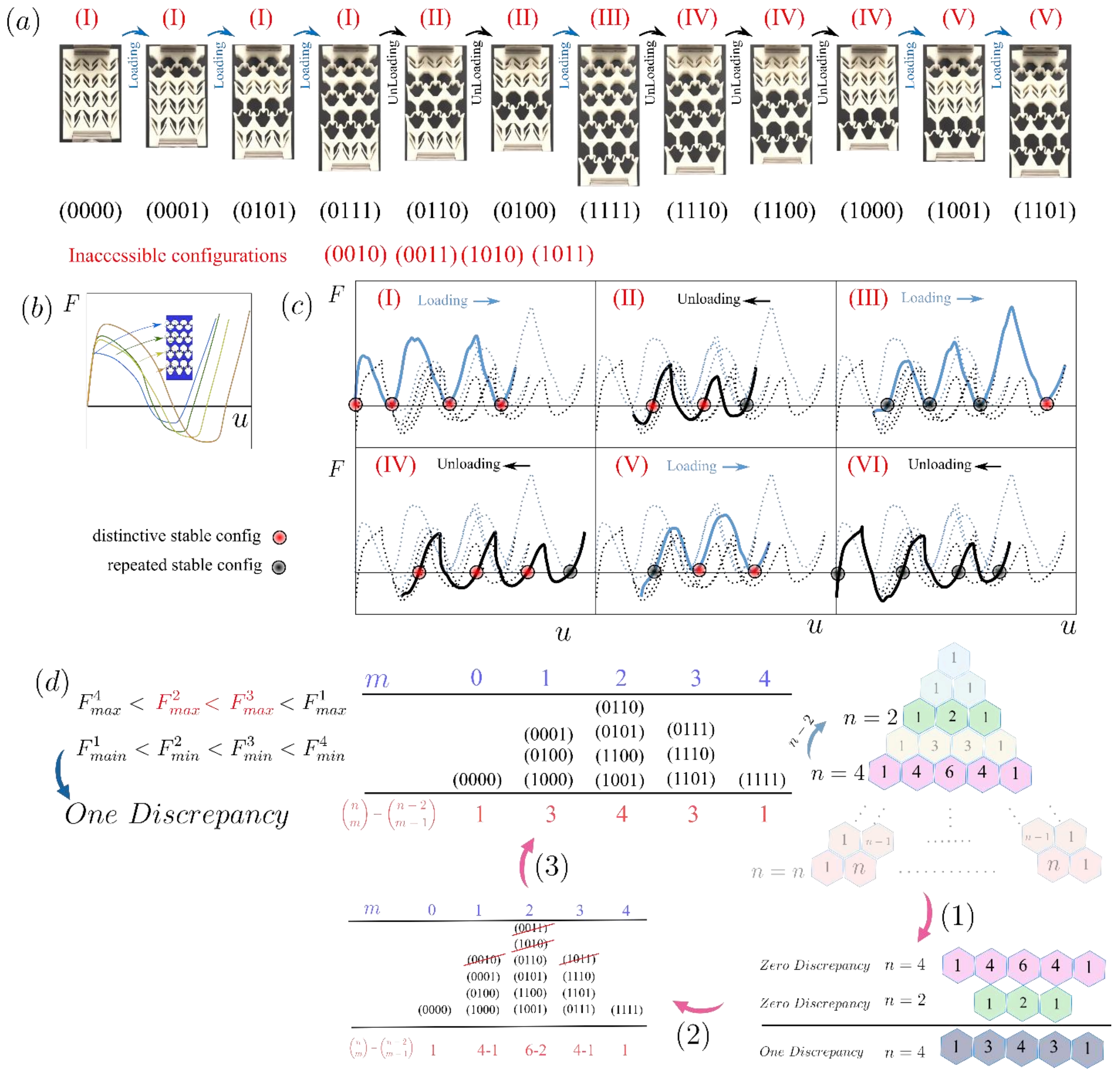

Figure S37. Experimental study of deformation sequence in multistable chains with one discrepancy. The chain comprises four bistable inclined beams, characterized by $F^4_{max} < F^2_{max} < F^3_{max} < F^1_{max}$, and $F^1_{min} < F^2_{min} < F^3_{min} < F^4_{min}$. **(a)** 12 stable configurations of the chain and the way to achieve the configurations according to F_{max}, and F_{min} orders. Also, the capital Greek letters illustrate the number that the direction of the applied displacement changes to capture the configurations. **(b)** Force-displacement curves of the cells corresponding to the chain. **(c)** deformation path in a controlled displacement experiment in which the blue and black dashed lines represent the experimental loading and unloading paths, respectively. The solid lines illustrate a part of the path to capture the stable configurations, where the red sign depicts the distinct stable configurations while the black sign shows the duplicated stable configurations. **(d)** verification of the results by mathematical theory in which all the accessible stable configurations have been captured by both theory and experiments. Capturing the configurations has been divided into three steps, (1) finding the difference between “n” and “n-2” rows, where n is the number of cells. (2) finding the inaccessible configurations based on the proposed method. (3) verifying the results.

S3.3. Multistability in compression for coupled multistable shellular cell

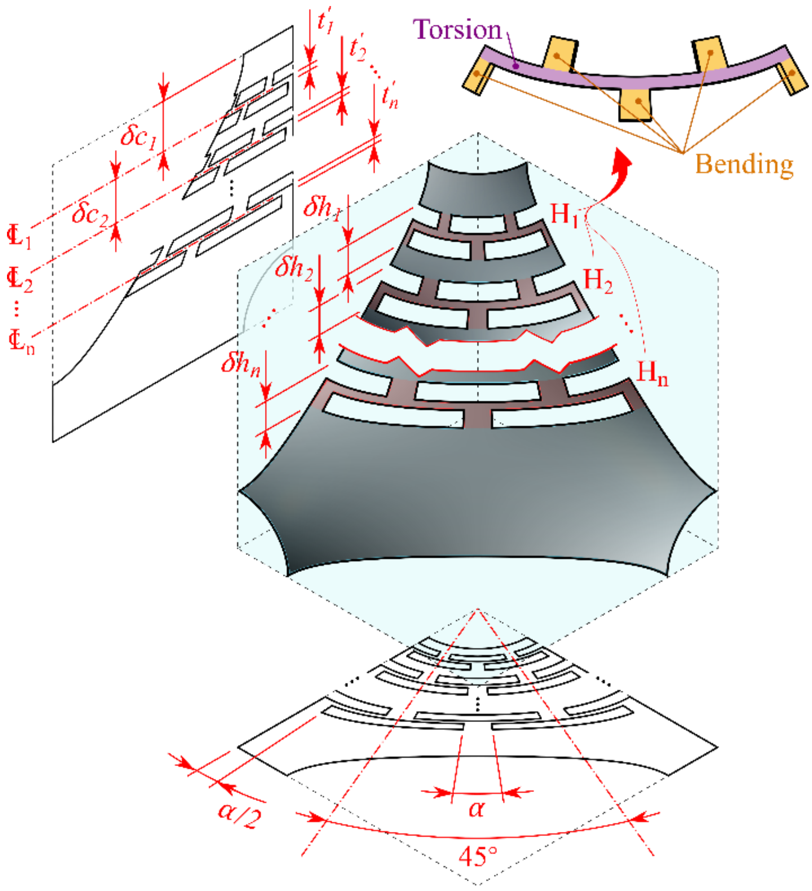

Figure S38. Multistability in compression. Nomenclature of the perforated shellular [4].

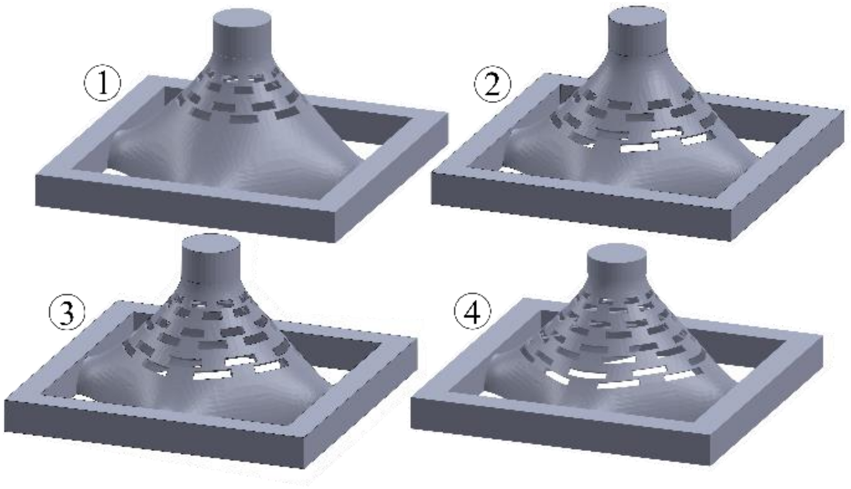

Figure S39. Multistable perforated shellular with various layers of bistable cells.

Table S9. Assigning the geometrical parameters of the perforated shellulars.

sample	α	n	t	c	h
**1**	6°	2	t_1=t_2=1mm	c_1=c_2=9mm	h_1=h_2=5mm
**2**	6°	2	t_1=t_2=1mm	c_1=18mm, c_2=9mm	h_1=h_2=5mm
**3**	6°	3	t_1=t_2=t_3=1mm	c_1=c_2=c_3=9mm	h_1=h_2=h_3=5mm
**4**	6°	4	t_1=t_2=t_3=t_4=1mm	c_1=9mm, c_2=c_3=c_4=7.5mm	h_1=h_2=h_3=h_4=5mm

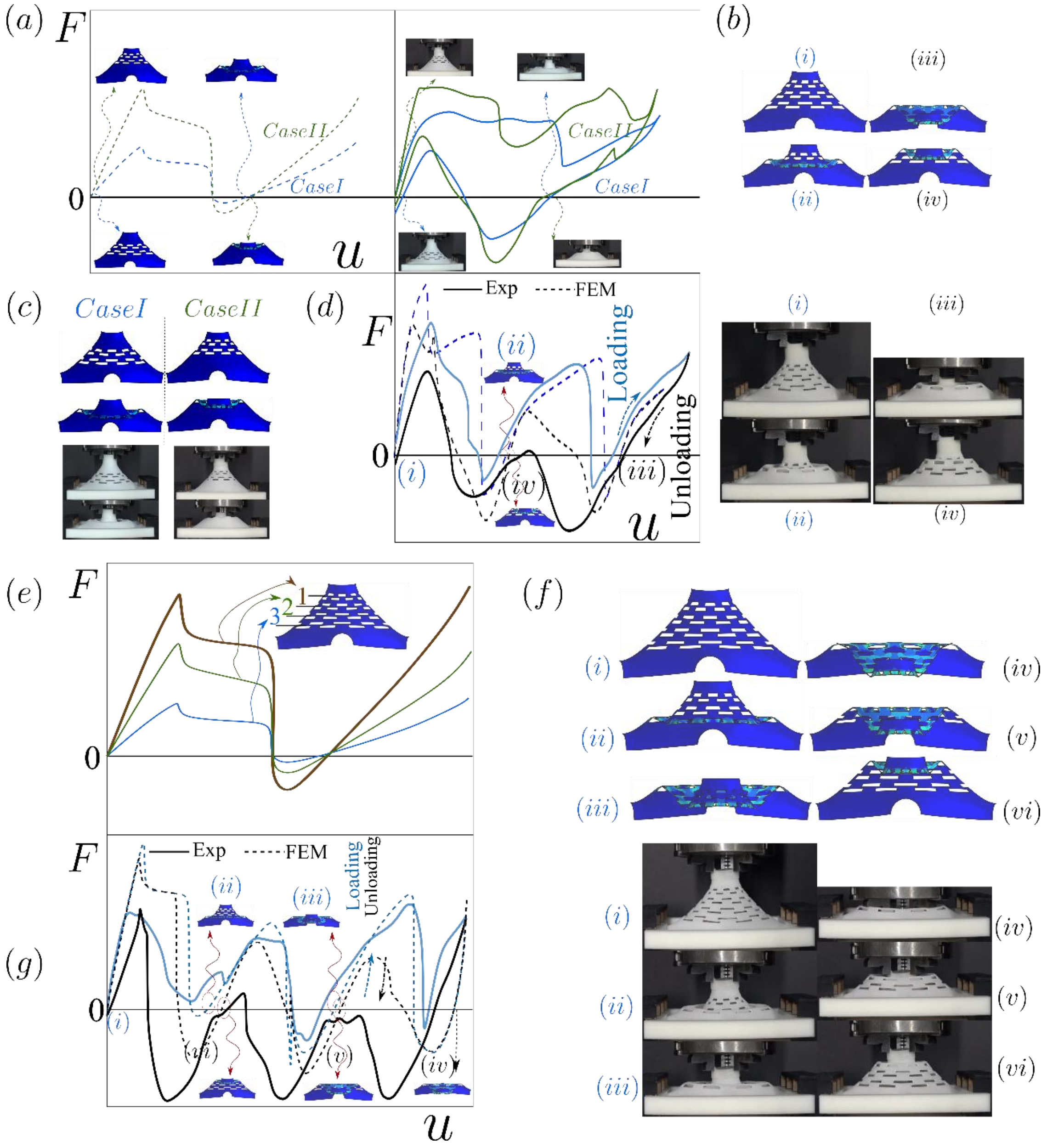

Figure S40. Multistability in compression for coupled multistable shellular cell.

S3.4. Wave like behavior in multistable materials

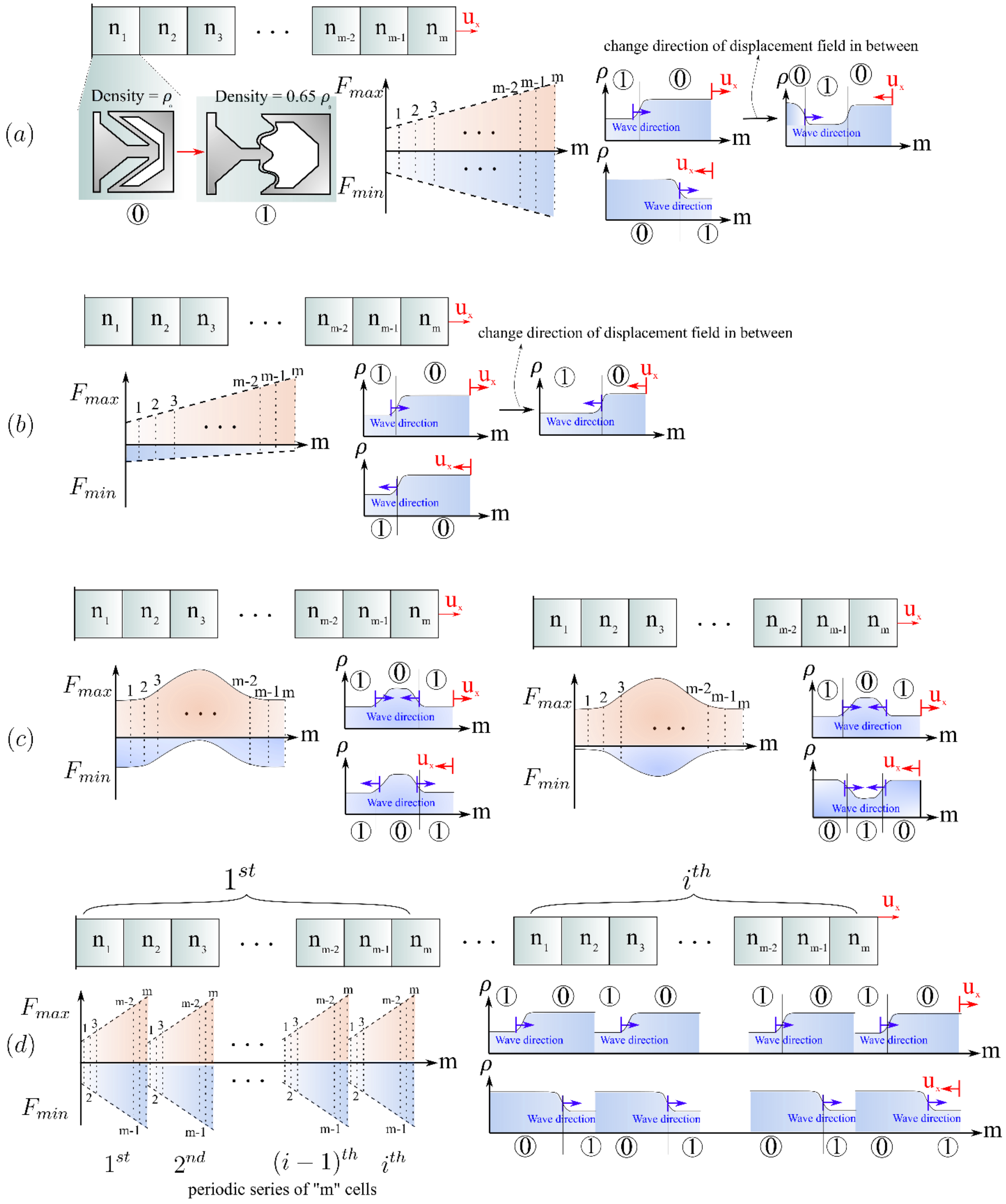

Figure S41. Wave Like Behavior in Multistable Materials. **(a)** definition of the density of unit cell based on stable configurations; the initial and second stable configurations have been defined by the densities of ρ, and 0.65ρ and the chain comprises "m" unit cell without discrepancy to investigate the wave propagation. By applying a whole cycle of controlled displacement (case I) to the (0000) configuration, the cells respectively pass through the initial (ρ) to second (0.65ρ) stable configuration based on the F_{max} orders. In this case, shape transformation and stimulus are in the same direction with one wavefront. However, by applying a whole cycle of the reversed controlled displacement (case II) to the (1111) configuration, the cells respectively turn back from second to the

initial stable configurations based on the F_{min} orders, by which the transformation direction remains unchanged while the stimulus direction has been reversed in compared to the loading path. If, before completing the cycle of the loading, the reversed displacement (case III) is applied to the chain, the transformation direction still remains unchanged, whereas two different wavefronts have appeared; one can be activated in loading and one in the unloading. **(b)** the chain comprises “m” unit cell with $m(m-1)/2$ discrepancies to investigate the wave propagation. In comparison to (a), case I remain unchanged, while by applying a whole cycle of the reversed controlled displacement (case II) to the (1111) configuration, the transformation direction and the stimulus direction are reversed in comparison to the loading path. If, before completing the loading cycle, the reversed displacement (case III) is applied to the chain, the transformation direction and the stimulus are both reversed. In all cases, there is one wavefront. **(c)** the chain comprises “m” unit cell with wave-like combinations of F_{max}, and F_{min}. **(d)** hierarchical metamaterial, comprising i^{th} chain, in which each chain comprises “m” cells without discrepancy. Interestingly, all the results of (a) remain unchanged.

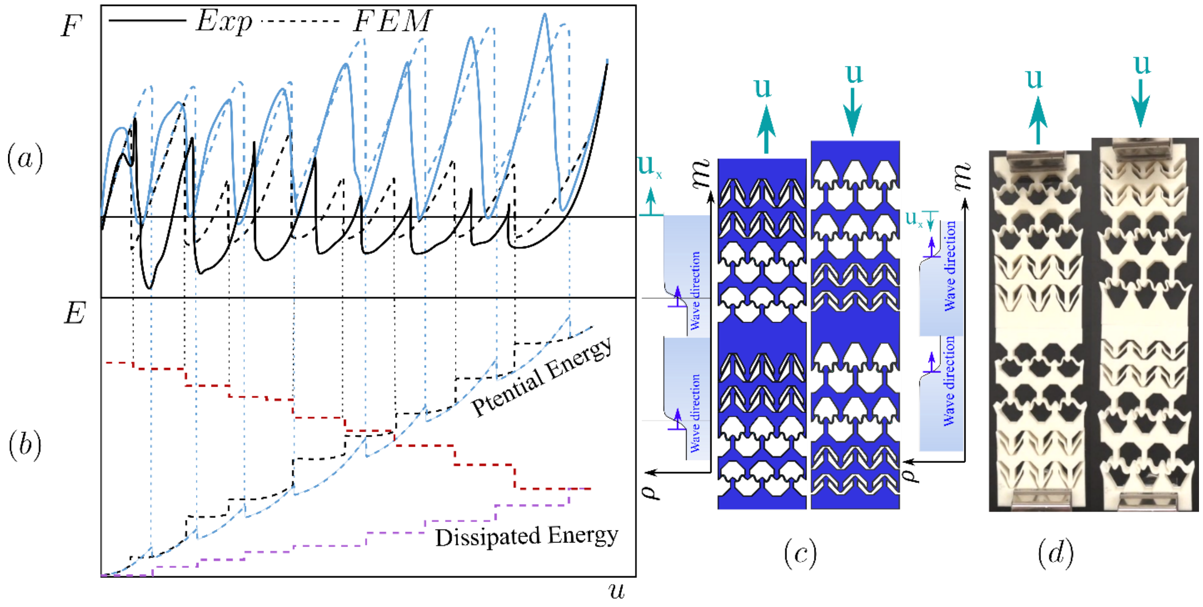

Figure S42. Wave Like Behavior in Multistable Materials**.** Experimental study of deformation sequence and wave propagation in a multistable metamaterial, comprising two chains in which each chain comprises four layers of the bistable inclined beam, characterized by $F_{max}^4 < F_{max}^2 < F_{max}^3 < F_{max}^1, and\ F_{min}^1 < F_{min}^2 < F_{min}^3 < F_{min}^4$. **(a)** Force-displacement curve of the materials in a controlled displacement experiment in which the blue and black solid lines represent the experimental loading and unloading paths, respectively, and the dashed lines illustrate the FEM paths. As shown, the two consecutive peaks correspond to the F_{max}^i $(i = 1, \dots, 4)$ of the chains, leading to that the deformation sequence still changes based on the F_{max}, and F_{min} orders, regardless, the number of layers in each chain and the number of the chain in the material. **(b)** Energy-displacement curve of the materials, in which the blue and purple dashed lines show the potential, and cumulative dissipation energy in the loading path, respectively, and the black and red dashed lines show the potential, and cumulative dissipation energy in the unloading path, respectively. As shown, by passing through the loading cycle and turning back from the unloading, a part of the absorbed energy by the material will continuously be dissipated. **(c)** computational study of the wave propagation in the chain. As illustrated, the deformation sequence occurs based on the F_{max}, and F_{min} orders and

the shape transformation and stimulus are in the same direction in loading. **(d)** verification of the results by the displacement control experiment in which all the results are entirely in a good agreement.

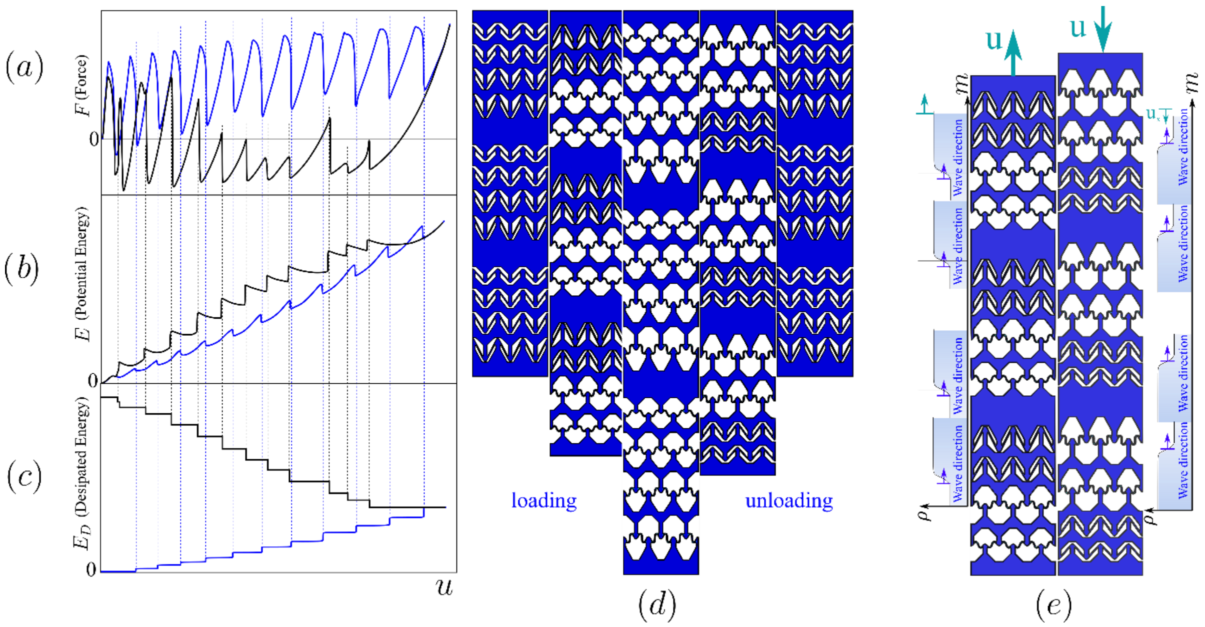

Figure S43. Wave Like Behavior in Multistable Materials. Computational study of deformation sequence and wave propagation in a multistable metamaterial, comprising three chains in which each chain comprises four layers of the bistable inclined beam, characterized by $F^4_{max} < F^2_{max} < F^3_{max} < F^1_{max}, and\ F^1_{min} < F^2_{min} < F^3_{min} < F^4_{min}$. **(a)** Force-displacement curve of the materials in a controlled displacement experiment in which the blue and black solid lines represent the computational loading and unloading paths, respectively. As shown, the four consecutive peaks correspond to the F^i_{max} $(i = 1, \ldots, 4)$ of the chains, leading to that the deformation sequence still changes based on the F_{max}, and F_{min} orders, regardless of the number of layers in each chain and the number of the chain in the material. **(b)** the potential energy-displacement curve of the materials in which the blue and black solid lines show the potential energy in loading, and unloading paths, respectively. **(c)** dissipation energy-displacement curve of the materials, in which the blue and black solid lines show the cumulative dissipation energy in loading, and unloading paths, respectively. As shown, by passing through the loading cycle and turning back from unloading, a part of the absorbed energy by the material will continuously be dissipated. **(d)** schematic sequence of deformation and stable configurations in loading and unlading paths. **(e)** computational study of the wave propagation in the material, comprising three chains. As illustrated, the deformation sequence occurs based on the F_{max}, and F_{min} orders and the shape transformation and stimulus are in the same direction in loading.

S4. Multistability in shear and SMA-like materials for programming chiral materials

Shape memory alloys can be deformed in low temperatures and retain their initial shape by heating the material, which means that the material has memory. As shown in Figure S44, shape memory alloys initially have martensite twinned crystal structures and by applying deformation, it goes to the martensite detwinned crystal structure. For reforming, it needs heat. Therefore, heat transforms the crystal structure into austenite. Also, by cooling the material, it comes back

to the initial twinned crystal structure. The material shows specific mechanical properties based on the new configuration. Then, due to the memory of the material, it can return to the initial form whenever needed. Bistability naturally has two stable conditions, which can be interpreted as a memory; therefore, multistability can expand the capacity of the material memory. In this work, we were looking for a multistable material with two properties. First, it shows different crystal structures by applying shear deformations. Second, it should be recoverable to any possible configuration. It is also well known that the targeted material properties are related to the Bravais lattice angels, while bistability in tension or compression does not change the angle. To change these angles, the unit cell should experience shear deformations. Also, bistability keeps the changes after removing the load. Therefore, a shear bistable element should be designed to capture our goal. Bistable elements in compression or tension have a supporting part that provides in-plane stiffness. However, it is challenging to design a supporting part without interfering deformations. Here, we overcome this challenge.

S4.1. Shear bistability

one degree of freedom tensegrity-like materials has been initially designed. Then, adding a string to the mechanism would interestingly facilitate generating an ideal bistable cell under a transverse loading in which the cell is locked in stable configurations. These configurations are entirely mirrored, leading that by increasing the F_{max}, F_{min} is also increased. This behavior is important in terms of designing deterministic material. Deterministic means that controlling the stable configurations is only related to the rigid element, regardless of the tension string, which is a variable in programmability. Therefore, controlling the instability forces are achievable by three factors: stiffness of the string, number of string rounds over the three hinges, and the prestress string. The prestressed string can be obtained by tightening the string and can increase the instability forces desired.

S4.2. Different crystal structures in 2D materials

As shown in Figure S45, a chain comprises four unit cells, and shows 16 distinct stable configurations in which 10 of them show monoclinic Bravais lattice, and the other six configurations represent six tetragonal Bravais lattices. Also, it shows six twinned and two detwinned structures. These transformations are also like SMA’s, except for the mode of austenite, which is obtained in heating. Here an instable state happens one by one, which means that we do not have a situation in which all cells are unstable. Here the memory of material is based on the bistability of the unit cells, and recalling the initial configuration is based on the shear deformations instead of the heating process. The manufactured prototype of these cells for two and three bistable unite cells without discrepancy have been shown in Figure S46, and

Figure S47, respectively. The deformation has been applied at the top side of the chain. Due to the existence of the shear through the beam, this design has the capability of making even multistable beams under three-point bending.

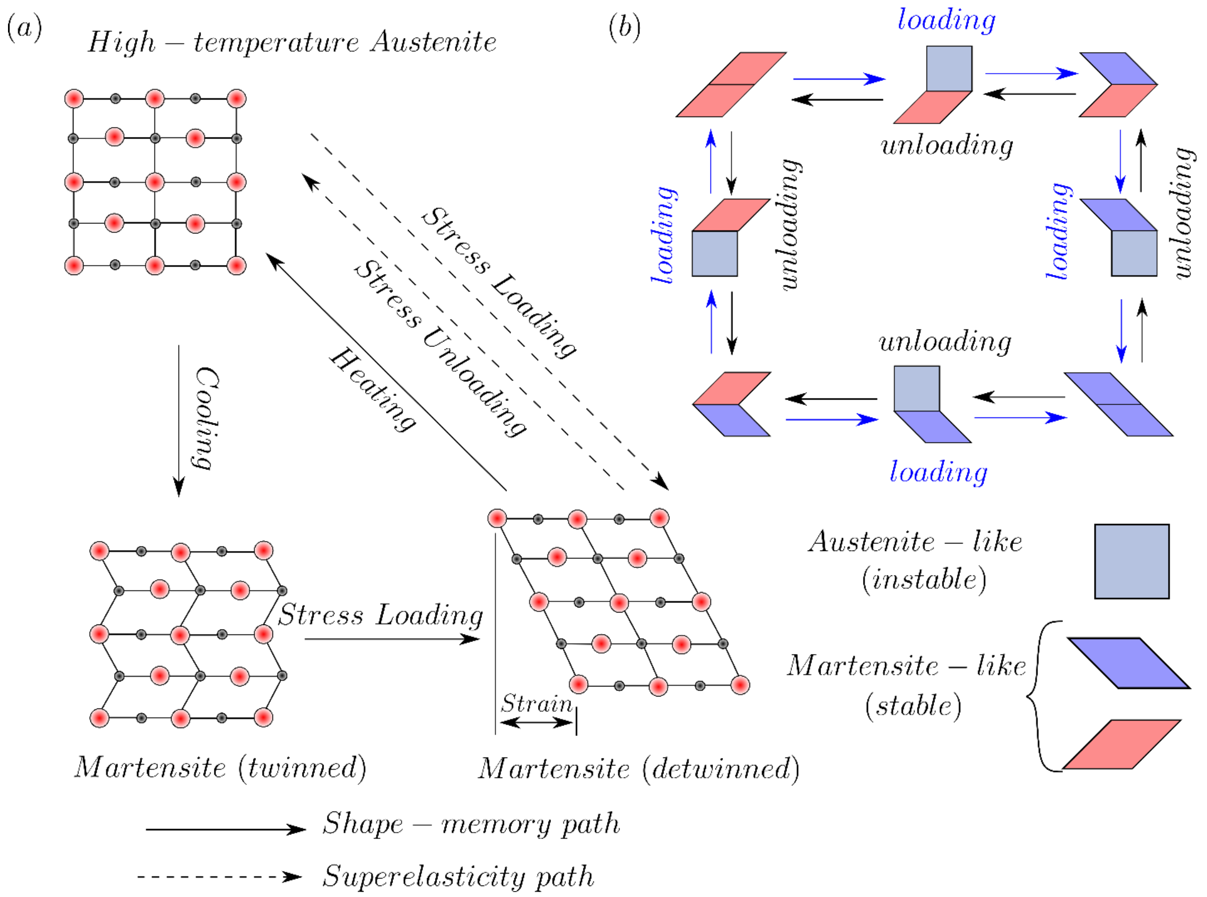

Figure S44. Shape memory alloys (SMAs). **(a)** martensite twinned crystal structure goes to the martensite detwinned crystal structure by applying deformation. For reforming, heat transforms the crystal structure to austenite, and by cooling the material, it comes back to the initial twinned crystal structures. The material has specific properties in each state and therefore, SMAs are known as memory. **(b)** mechanical memory. Designing shear bistable element in which by applying the forward and backward controlled displacement, the cells pass through (turn back from) the instability to the second (initial) stable state, where the instable state and stable states correspond to austenite and martensite crystal structure in SMAs.

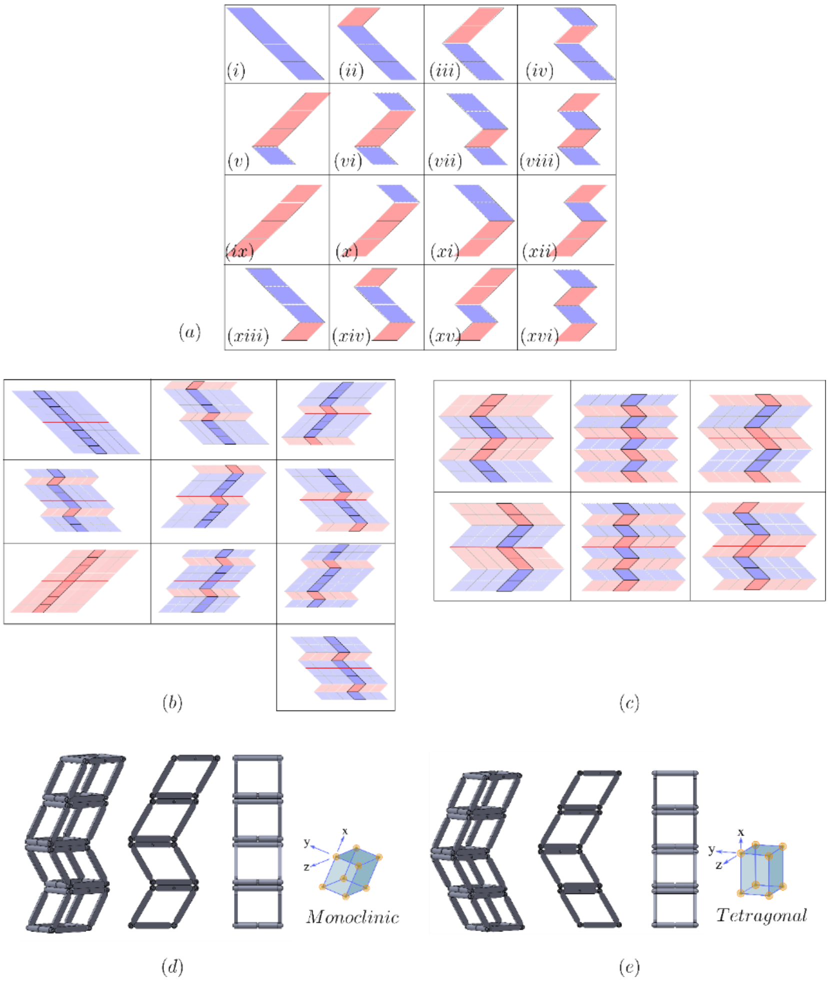

Figure S45. A chain comprises four shear bistable unit cells, characterized by $F_{max}^{4\,(top)} < F_{max}^{3} < F_{max}^{2} < F_{max}^{1\,(bottom)}$, and $F_{min}^{1\,(bottom)} < F_{min}^{2} < F_{min}^{3} < F_{min}^{4\,(top)}$ **(a)** in which the initial, and second stable states have been defined by blue and red parallelograms, respectively, and the 16 stable configurations have been shown by Greek letters. **(b)** Based on the arrangement of F_{max} in loading and F_{min} in unloading, 10 monoclinic crystal structure has been obtained in a material comprised of two defined chains. **(c)** Based on the orders, six tetragonal crystal structure has been obtained in the material. **(d)**, and **(e)** illustration of monoclinic and tetragonal crystal structures in the chain based on the designed shear bistable element.

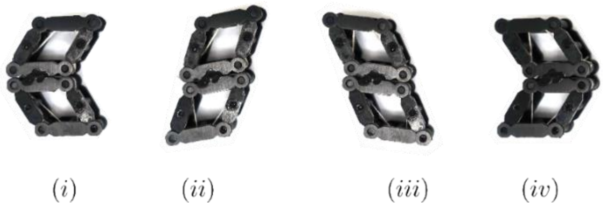

Figure S46. Multistability in shear. The fabricated chain comprises two shear bistable unit cells without discrepancy, leading to four stable configurations.

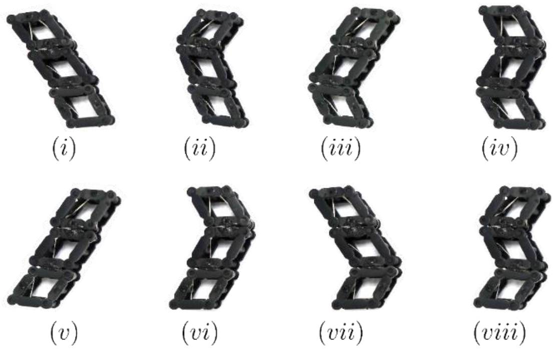

Figure S47. Multistability in shear. The fabricated chain comprises three shear bistable unit cells without discrepancy, leading to eight stable configurations.

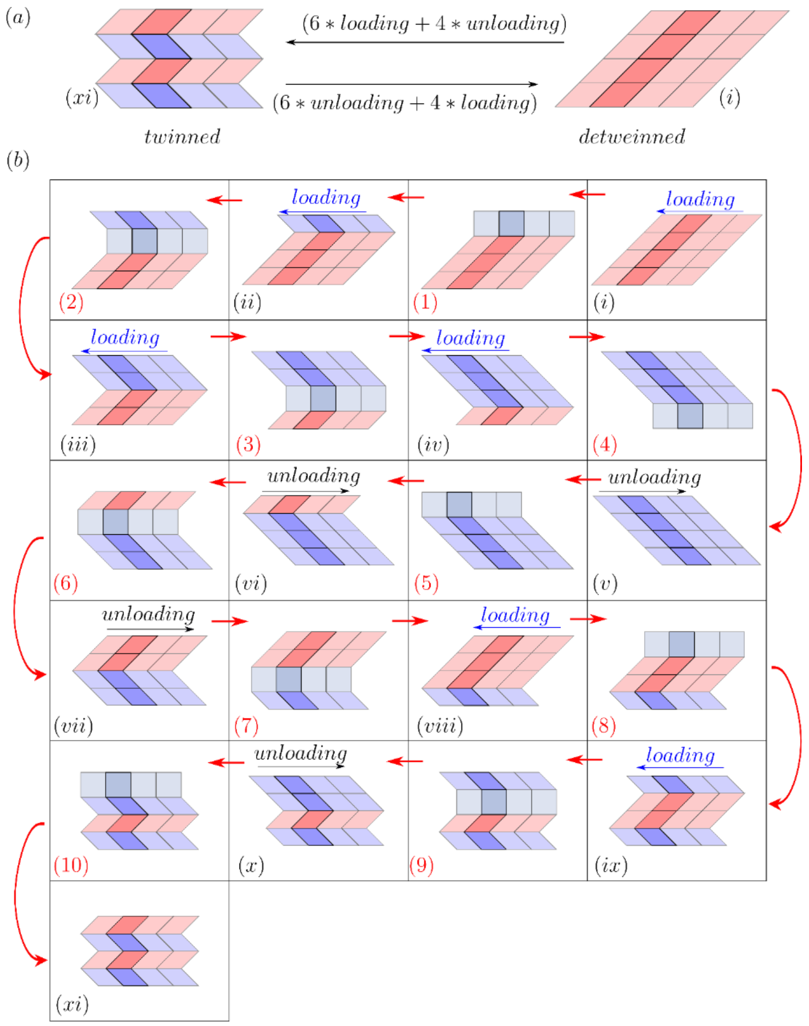

Figure S48. Programmability of 2D chiral material. A chain comprises four shear bistable unit cells, characterized by $F_{max}^{4\,(top)} < F_{max}^{3} < F_{max}^{2} < F_{max}^{1\,(bottom)}$, and $F_{min}^{1\,(bottom)} < F_{min}^{2} < F_{min}^{3} < F_{min}^{4\,(top)}$, has been shown. **(a)** initial (0000), and final (0101) stable configurations of the chain, corresponding to the detwinned-like and twinned-like crystal structures in SMAs, respectively, have been shown by (i), and (xi) configurations. The initial configuration (detwinned-like crystal structure) has been converted to the final configuration (twinned-like crystal structure) by applying the proposed deformation. **(b)** for achieving (xi) configuration, step by step path has been displayed. As shown, the Greek letters demonstrate the stable configurations, and the natural numbers illustrate the unstable state (austenite-like crystal structure).

S4.3. Different crystal structures in 3D materials

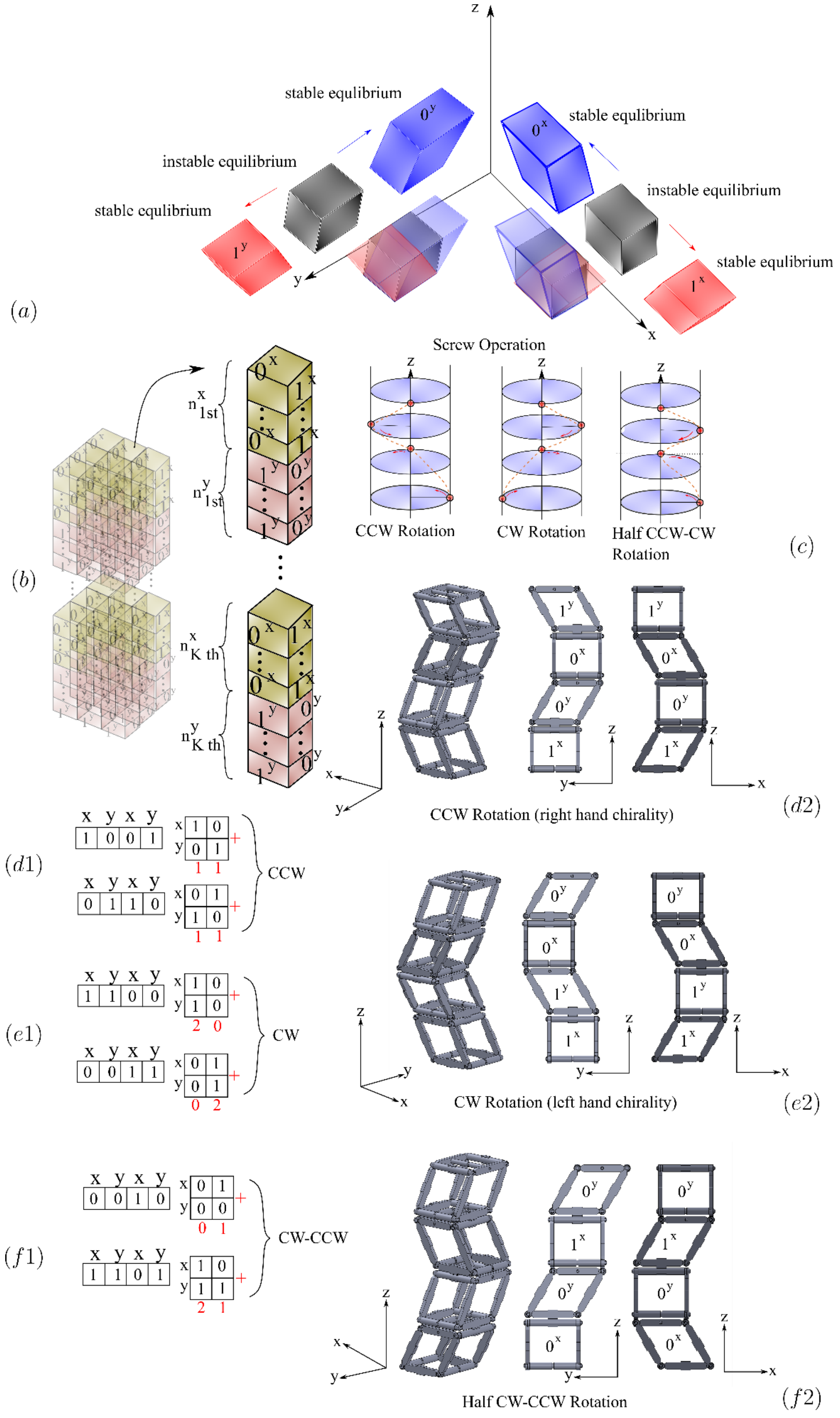

Figure S49. Programmability of 3D chiral material. **(a)** schematic of two-directional bistable structure. **(b)** definition of the number of cells that have similar bistability. **(c)** definition of the rotations and the material transformation line to classify the structures. **(d)** definition of the CCW chiral material with further insight. **(d1)** definition of summation on bistable states in x and y-directions. X and y are the directions of the bistability and zero and one are the initial and second stable states, respectively. By adding two nearest neighbor cells, the results would be equal. **(d2)** schematic of CCW chiral material. **(e)** Definition of the CW chiral material with further insight. **(d1)** by adding two nearest neighbor cells, the cells in second stable configurations add up. **(e2)** schematic

of CW chiral material. **(f)** Definition of the Half CW-CCW material with further insight. **(f1)** if cases (d) and (e) do not happen, the chain is polar material. **(f2)** schematic of Half CW-CCW chiral material.

S5. Study of chirality in multistable materials

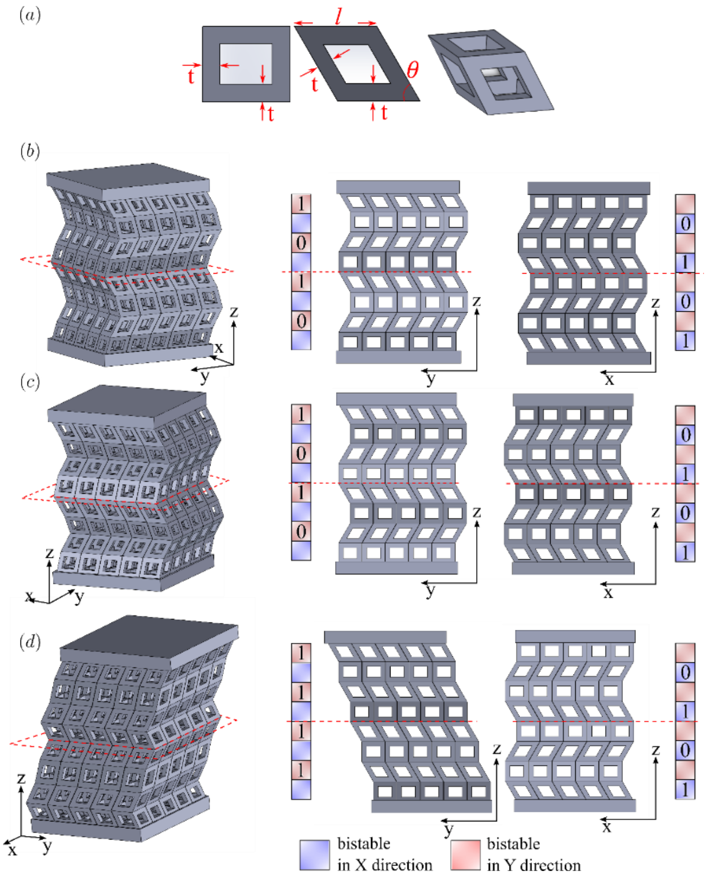

Figure S50. Tessellation of the structure to design a chiral metamaterial. **(a)** nomenclature of the shear bistable cell. **(b)**, **(c)**, and **(d)** tessellated CCW, CW, and Half CW-CCW chiral materials.

S5.1. Theoretical analysis

As shown in Figure S51, we tried to propose a simple beam analysis for the helical structure to study some parameters for tuning the chirality of the material. Although considering these structures as the beam is questionable, with an acceptable error, the trend of these results is valid for one set of unit cells. It means that if the chain has been tessellated in the x and y directions, the absolute value of the results will change, even though the pattern of these results are simalar to the the tessellated chain.

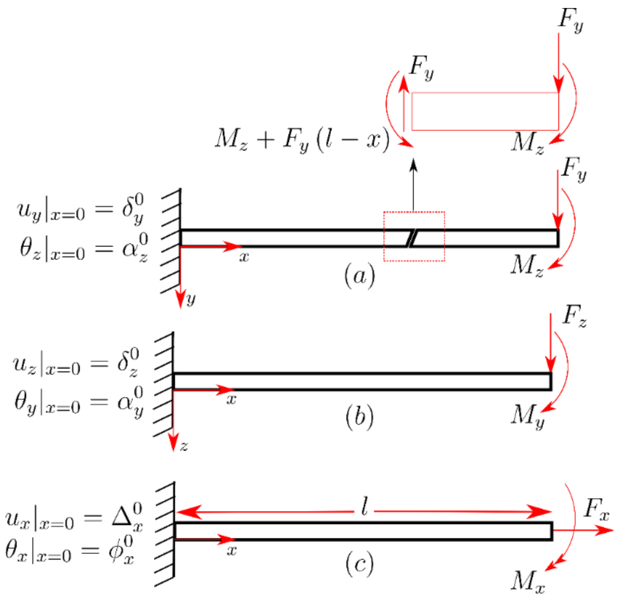

Figure S51. A simplified beam model to study the shear bistable chain has been proposed. Based on the linear analysis and superposition theory, the 3D beam model has been divided into two bendings and one torsional part, in which the applied forces, moments, and boundary conditions have been shown. **(a)** and **(b)** analyzing the beam under the shear force and bending moment. **(c)** studying the beam under the axial force and torsional moment.

By superposing the beams in Figure S51, 3D beams would be studied based on the Euler–Bernoulli beam theory.

$$\frac{d^2u_y}{dx^2} = -\frac{M_z}{EI_z}, \tag{3}$$

where, $M_z = -M_z^0 - F_y^0(l-x)$. Also, the boundary conditions have been illustrated as follows,

$$\begin{aligned} u_y\big|_{x=0} &= \delta_y^0 \qquad & u_z\big|_{x=0} &= \delta_z^0 \qquad & u_x\big|_{x=0} &= \Delta_x^0 \\ \theta_y\big|_{x=0} &= \alpha_y^0 \qquad & \theta_z\big|_{x=0} &= \alpha_z^0 \qquad & \theta_x\big|_{x=0} &= \varphi_x^0 \end{aligned} \tag{4}$$

By solving the equations for shown beams in Figure S51, the displacement field has been obtained as follows,

$$\begin{aligned} u_x &= \frac{F_x^0 l}{EA} + \Delta_x^0 \ , \\ \varphi_x &= \frac{M_x^0 l}{GJ} + \varphi_x^0 . \end{aligned} \tag{5}$$

$$u_y = -\frac{F_y^0}{6EI_y}x^3 + \left(M_z^0 + F_y^0 l\right)\frac{x^2}{2EI_y} + \alpha_y^0 x + \delta_y^0 , \tag{6}$$

$$\theta_y = -\frac{F_y^0}{2EI_y}x^2 + \left(M_z^0 + F_y^0 l\right)\frac{x}{EI_y} + \alpha_y^0 .$$

$$u_z = -\frac{F_z^0}{6EI_z}x^3 + \left(M_y^0 + F_z^0 l\right)\frac{x^2}{2EI_z} + \alpha_z^0 x + \delta_z^0 \quad , \tag{7}$$

$$\theta_z = -\frac{F_z^0}{2EI_z}x^2 + \left(M_y^0 + F_z^0 l\right)\frac{x}{EI_z} + \alpha_z^0 .$$

It can be easily shown,

$$\begin{bmatrix} u_y \\ u_z \\ \theta_y \\ \theta_z \\ u_x \\ \varphi_x \end{bmatrix} = \begin{bmatrix} \frac{l^2}{2EI_y} & 0 & \frac{l^3}{3EI_y} & 0 & 0 & 0 \\ 0 & \frac{l^2}{2EI_z} & 0 & \frac{l^3}{3EI_z} & 0 & 0 \\ \frac{l}{EI_y} & 0 & \frac{l^2}{2EI_y} & 0 & 0 & 0 \\ 0 & \frac{l}{EI_z} & 0 & \frac{l^2}{2EI_z} & 0 & 0 \\ 0 & 0 & 0 & 0 & \frac{l}{EA} & 0 \\ 0 & 0 & 0 & 0 & 0 & \frac{l}{GJ} \end{bmatrix} \begin{bmatrix} M_z^0 \\ M_y^0 \\ F_y^0 \\ F_z^0 \\ F_x^0 \\ M_x^0 \end{bmatrix} + \begin{bmatrix} \delta_y^0 + \alpha_y^0 l \\ \delta_z^0 + \alpha_z^0 l \\ \alpha_y^0 \\ \alpha_z^0 \\ \Delta_x^0 \\ \varphi_x^0 \end{bmatrix} . \tag{8}$$

Therefore,

$$\begin{bmatrix} u_x & \theta_y & \theta_z \\ \theta_y & u_y & \varphi_x \\ \theta_z & \varphi_x & u_z \end{bmatrix} = \mathbf{S} \begin{bmatrix} F_x^0 & F_y^0 & F_z^0 \\ F_y^0 & M_z^0 & M_x^0 \\ F_z^0 & M_x^0 & M_y^0 \end{bmatrix} + \begin{bmatrix} \Delta_x^0 & \alpha_y^0 & \alpha_z^0 \\ \alpha_y^0 & \delta_y^0 + \alpha_y^0 l & \varphi_x^0 \\ \alpha_z^0 & \varphi_x^0 & \delta_z^0 + \alpha_z^0 l \end{bmatrix} . \tag{9}$$

where $\mathbf{S}$ is the compliance tensor.

Here, the rotation matrix has been defined to extend the results for all beams in the chain.

$$\bar{\mathbf{T}} = \mathbf{Q}\mathbf{T}\mathbf{Q}^{\mathrm{T}} , \tag{10}$$

where, $\mathbf{Q} = \mathbf{R_x}\mathbf{R_y}\mathbf{R_z}$, and

$$\mathbf{R}_z = \begin{bmatrix} \cos\alpha & \sin\alpha & 0 \\ -\sin\alpha & \cos\alpha & 0 \\ 0 & 0 & 1 \end{bmatrix}, \ \mathbf{R}_x = \begin{bmatrix} 1 & 0 & 0 \\ 0 & \cos\beta & \sin\beta \\ 0 & -\sin\beta & \cos\beta \end{bmatrix},$$
$$\mathbf{R}_y = \begin{bmatrix} \cos\gamma & 0 & \sin\gamma \\ 0 & 1 & 0 \\ -\sin\gamma & 0 & \cos\gamma \end{bmatrix}. \quad (11)$$

In which, α, β, and γ have been obtained as follows,

$$\cos\alpha = \frac{\mathbf{n}.\mathbf{e}_x}{\|\mathbf{n}\|\|\mathbf{e}_x\|}, \quad \cos\beta = \frac{\mathbf{a}.\mathbf{e}_y}{\|\mathbf{a}\|\|\mathbf{e}_y\|}, \quad \cos\gamma = \frac{\mathbf{b}.\mathbf{e}_z}{\|\mathbf{b}\|\|\mathbf{e}_z\|}. \quad (12)$$

where, $\mathbf{n}.\bar{\mathbf{x}} = const.$, and $\mathbf{n}.\mathbf{a} = 0$, $\mathbf{n} \times \mathbf{a} = \mathbf{b}$. Finally, the results have been obtained as follows.

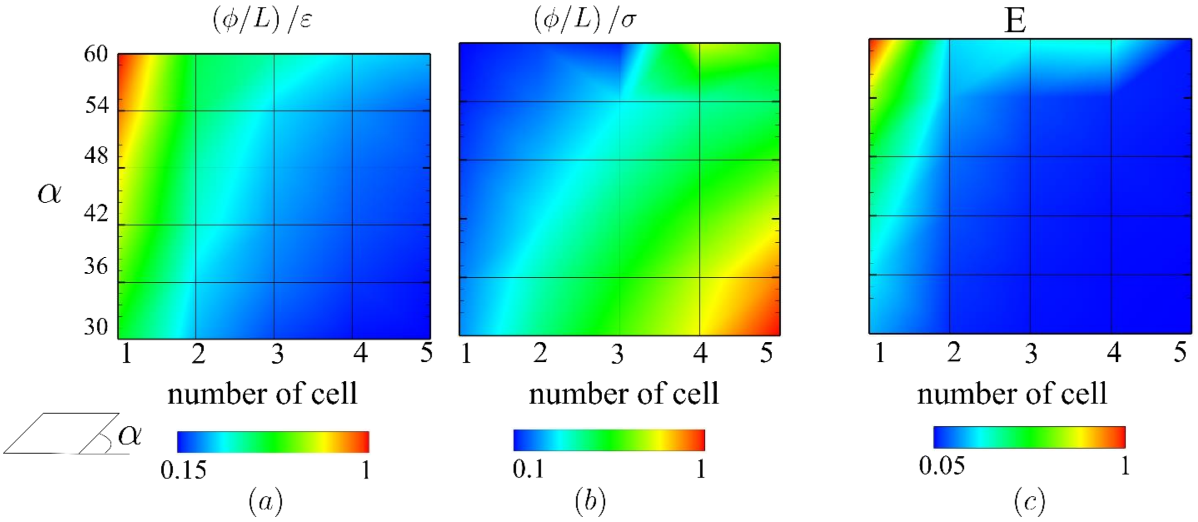

Figure S52. Effect of rotational degree of freedom (RDOF) and the number of the cells on **(a)** chirality in a constant strain, **(b)** chirality in constant stress, and **(c)** stiffness. Increasing the RDOF decreases eccentric parameters, leading to increasing the chirality in constant strain. However, increasing the angle can decrease chirality in constant stress at infinitesimal strain. This behavior stems from stiffness variation relative to the angle, in which by increasing the angle, Young's modulus increases.

According to Figure S52, increasing the α up to 60 degrees increases the curvature at a constant compressive strain. In contrast, it has an opposite effect on the obtained curvature under constant compressive stress. The opposite behavior happens when the number of the same oriented series cells increases. By increasing the number of cells under constant strain the curvature decreases, while this curvature increases at the constant force. The eccentricity increases by increasing the number of cells in each orientation or by decreasing α. This study was limited to the $\pi/6 \leq \alpha \leq \pi/3$ as an acceptable angle for shear bistable cells. In this domain of α, eccentricity determines the curvature under constant stress or strain. Higher eccentricity leads to higher curvature under constant stress, and lower eccentricity leads to

higher curvature under constant strain. This difference comes from the change in the elastic modulus of the material. By increasing the eccentricity, the elastic modulus drastically decreases and makes the material compliant.

S5.2. Stress concentration

Note that two definitions should be comprehensively illustrated. Chirality direction is defined based on the geometry of the cells, while the rotation direction depends on the geometry and direction of the loading. For instance, the CCW structure shows CW and CCW rotation under compression and tension, respectively. Likewise, the CW structure shows CCW and CW rotation under compression and tension, respectively. Interestingly, the stress concentration zone is the same as structural chirality in both tension and compression, as shown in Figure S53, and Figure S54.

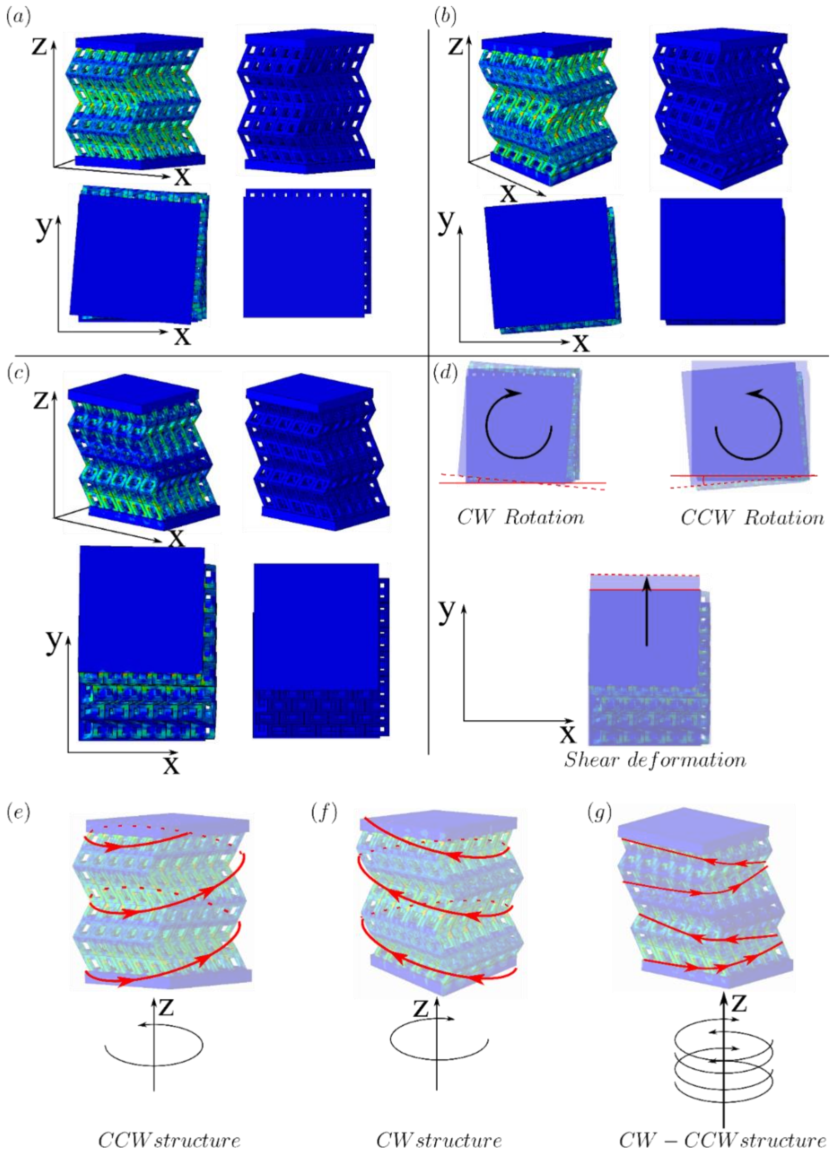

Figure S53. Stress concentration in compression. **(a)** CCW material under the compression. **(b)** CW material under the compression. **(c)** polar material under the compression. **(d)** deformation direction in CCW, CW, and polar

materials; the CCW, CW materials rotate in CW, CCW direction, and the polar materials show shear deformation. **(e)** note that chirality direction is defined based on the geometry of the cells. While the rotation direction depends on the geometry and direction of the loading. Here, stress concentration distribution has been shown in which it, quite literally, depends on the chirality direction of the material, regardless of the types of subjected loads.

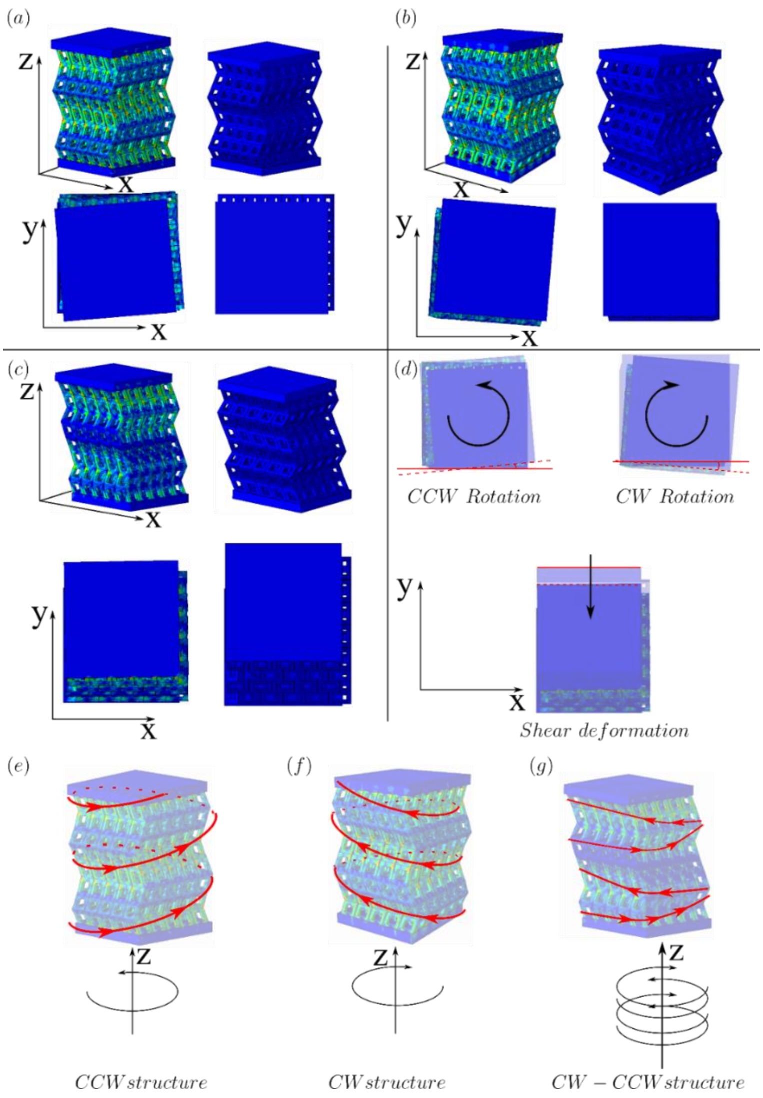

Figure S54. Stress concentration in tension. **(a)** CCW material under the tension. **(b)** CW material under the tension. **(c)** polar material under the tension. **(d)** deformation direction in CCW, CW, and polar materials; the CCW and CW materials rotate in CCW, CW direction, and the polar materials show shear deformation. **(e)** note that chirality direction is defined based on the geometry of the cells. While the rotation direction depends on the geometry and direction of the loading. Here, stress concentration distribution has been shown in which it, quite literally, depends on the chirality direction of the material, regardless of the types of subjected loads.

S6. Application of the Multistability in Memory Devices and Sensors

In the multistable material, a specific number of configurations are available from one external displacement field (set of loading and unloading). These configurations can be representative of a specific sign or number. For instance, in memory cells, there are 0 and 1, which are related to the two stable configurations. These two configurations can give two different current values

in an electrical board if it is considered as an on-off switch. One challenge in the memory industry is the capacity of the memory. Reading and writing in the memory can be done by an external head, implying displacement. Our methodology shows that by programming the material, 2^n configurations are achievable, and each configuration can be represented by a specific currency. However, this increase of capacity has one challenge; when the memory needs to reach $(101...101)$ the speed of writing is slower than one bistable switch $(0$ or $1)$. However, these configurations reduce the need for the head moves (if we have one head for writing the memory). Moreover, this history-dependent material behavior can also be used as a sensor. Each stage of material can give information about the applied stress and displacements on the material, which can be used as a sensor that can be read once and obtain the probable path (worst case scenario). For obtaining the worst-case scenario, we also need the number of shapes transforming in the material. The number of shapes transforming can be obtained by a simple counter. The current situation also gives users the probable paths that reach this point. By having the number of shapes transforming in the material, the worst case that is compatible with this configuration is achievable. Here the worst-case scenario is defined as a case the material was under the highest applied stress.

S.6.1. Sensor circuit

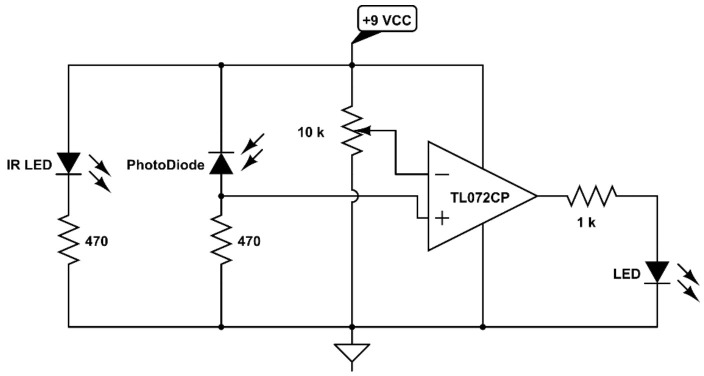

Figure S55. Sensor circuit. The pair of IR LED, while PhotoDiode captures the distance. The circuit is connected to $9V$ power supply and TL072CP is a general operational amplifier that has been connected to a photodiode, $10k\Omega$ potentiometer, and $1k\Omega$ resistor along with LED.

S6.2. Sensor/memory data conversion (digital to analog D/A)

Here, three cases have been investigated in detail.

S6.2.1. Case I

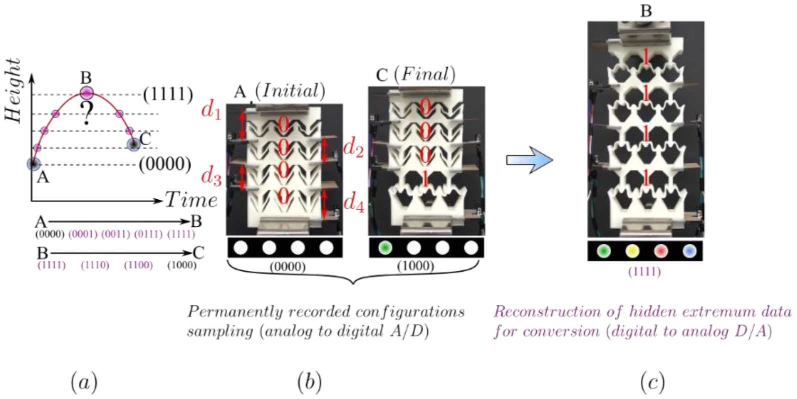

Figure S56. Mechanical memory device (Case I). **(a)** Time evolution of the height variation in the monitored system, in which **(b)** is the recorded data, and **(c)** is the reconstructed extremum data that has been saved in the history of mechanical memory based on the accessible paths and deformation sequences.

Table S10. Detailed study of the condition pertinent to case I.

State	Max $\{\boldsymbol{min_1}, \boldsymbol{min_2}\ , \ldots\}$	Min $\{\boldsymbol{max_1}, \boldsymbol{max_2}\ , \ldots\}$	condition
(0000)	$-\uparrow$	$-$	
(0001)	(0000)	$-$	valid
(0011)	(0000)	$-$	valid
(0111)	(0000)	$-$	valid
(1111)	(0000)	(1111)	valid
(1110)	(0000)	(1111)	valid
(1100)	(0000)	(1111)	valid
(1000)	$-$	$-$	

Table S11. Detailed study of the reconstruction function pertinent to case I.

	K	j	m	L	State
(A)	1	–	3	L = (1)(4)-(0) = 4	(0000)
	1	1	3	4	(0001)
	1	2	3	4	(0011)
	1	3	3	4	(0111)
(B)	2	4	3	L = (-1)(3) = -3	(1111)
	2	1	3	-3	(1110)
	2	2	3	-3	(1100)
(C)	–	–	3	–	(1000)

S6.2.2. Case II

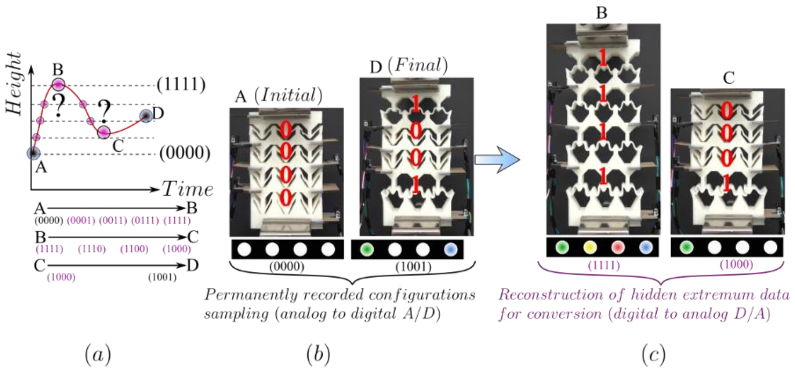

Figure S57. Mechanical memory device (Case II). **(a)** time evolution of the height variation in the monitored system, in which **(b)** is the recorded data, and **(c)** is the reconstructed data that has been saved in the history of mechanical memory based on the accessible paths and deformation sequences.

Table S12. Detail study of the condition pertinent to case II.

state	Max $\{min_1, min_2\ , \ldots\}$	Min $\{max_1, max_2\ , \ldots\}$	condition
(0000)	–↑	–	
(0001)	(0000)	–	valid
(0011)	(0000)	–	valid
(0111)	(0000)	–	valid
(1111)	(0000)	(1111)	valid
(1110)	(0000)	(1111)	valid
(1100)	(0000)	(1111)	valid
(1000)	(0000)	(1111)	valid
(1001)	–	–	

Table S13. Detail study of the reconstruction function pertinent to case II.

	K	j	m	L	State
(A)	1	–	3	L=(1)(4)-(0)=4	(0000)
	1	1	3	4	(0001)
	1	2	3	4	(0011)
	1	3	3	4	(0111)
(B)	2	4	3	L=(-1)(3)=-3	(1111)
	2	1	3	-3	(1110)
	2	2	3	-3	(1100)
(C)	3	3	3	L=(1)(1)=1	(1000)
(D)	–	–	3	–	(1001

S6.2.3. Case III

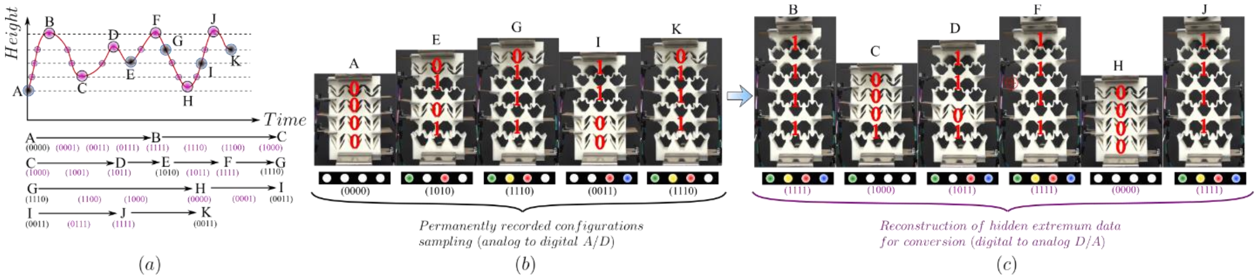

Figure S58. Mechanical memory device (Case III). **(a)** time evolution of the height variation in the monitored system, in which **(b)** is the recorded data, and **(c)** is the reconstructed data that has been saved in the history of mechanical memory based on the accessible paths.

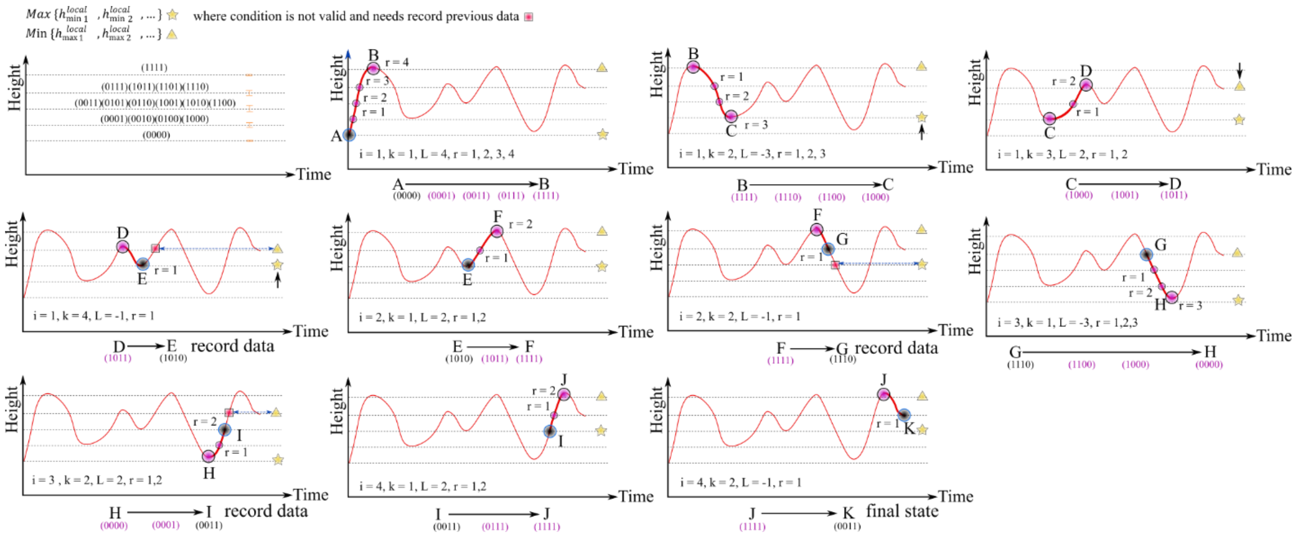

Figure S59. Detail study of the reconstruction in case III. The defined parameters, recorded data, and reconstructed data have been shown step by step.

For clarification, the results of Figure S59 have precisely been illustrated in the following table.

Table S14. Detail study of the condition pertinent to case III.

	State	min	max	Condition
	A(0000)	–↑	–	
	(0001)	(0000)	–	valid
	(0011)	(0000)	–	valid
	(0111)	(0000)	–	valid
	B(1111)	(0000)	(1111)	valid
	(1110)	(0000)	(1111)	valid
	(1100)	(0000)	(1111)	valid
	C(1000)	(0000)	(1111)	valid
	(1001)	Max {(0000),(1000)}= (1000)	(1111)	valid
	D(1011)	(1000)	(1111)	valid
* (Aliasing)	E(1010)	(1000)↑	min{(1111),(1011)}=(1011)	valid
	(1011)	(1010)	–	Not valid
	F(1111)	(1010)	–	valid
* recorded	G(1110)	–	–↓	valid
	(1100)	–	(1110)	Not valid
	(1000)	–	(1110)	valid
	H(0000)	–	(1110)	valid
	(0001)	(0000)	(1110)	valid
* recorded	I(0011)	–↑	–	valid
	(0111)	–	(0011)	Not valid
	J(1111)	–	(0011)	valid
	K(1110)	–	–	

***This step should be saved because the next step condition is not valid, and we will lose the (1010) state and we cannot reconstruct and reproduce all paths.

Table S15. Detail study of the reconstruction function pertinent to case III.

	K	j	m	L	State
(A)	1	–	3	L=(1)(4)-(0)=4	(0000)
	1	1	3	4	(0001)
	1	2	3	4	(0011)
	1	3	3	4	(0111)
(B)	2	4	3	L=(-1)(3)=-3	(1111)
	2	1	3	-3	(1110)
	2	2	3	-3	(1100)
(C)	3	3	3	L=(1)(2)=2	(1000)
	3	1	3	2	(1001)
(D)	4	2	3	L=(-1)(1)=-1	(1011)
(E)	1	–	3	L=(1)(3)-(1)=2	(1010)
	1	1	2	2	(0011)
(F)	2	2	2	L=(-1)(1)=-1	(1111)
(G)	1	–	3	L=(-1)(4)+(1)=-3	(1110)
	1	1	3	-3	(1100)
	1	2	3	-3	(1000)
(H)	2	3	3	L=(1)(2)=2	(0000)
	2	1	3	2	(0001)
(I)	1	–	3	L=(1)(4)-(2)=2	(0011)
	1	1	3	2	(0111)
(J)	2	2	3	L=(-1)(1)=-1	(1111)
(K)	–	–	3	–	(1110)

S7. Simulation method

Commercial finite element software ABAQUS is used to conduct numerical simulations on the designed metamaterials and meta structures. Specifically, the material parameters have been mainly used in numerical simulation based on the average stiffness of the mechanical properties of thermoplastic polyurethane (TPU). Furthermore, if not specially mentioned, a nonlinear dynamic implicit solver is adopted, and a quasi-static condition is ensured by checking the kinetic energy during deformation.

S8. Manufacturing

S8.1 Chraractrised chain comprised of two unit cell

unit cells have been designed and 3D printed by fused deposition modeling (FDM) using an Ultimaker S3 3D printer. Unit cells are printed out of thermoplastic polyurethane (TPU (95A)), and polylactic acid (PLA).

S8.2 Multistable chains (chain comprised of 4 bistable unit cells with inclined beam and sinusoidal beam elements and shellular perforated structure)

For chains comprised of 4 bistable unit cells with inclined beam and sinusoidal beam elements and shellular perforated samples, Sinterstation 2500 with 50W CO2 laser is used to 3D print TPU materials (SLS Technology). Its maximum build size is 25.4 cm × 25.4 cm × 34.29 cm, with resolution in the X/Y plane as 0.75 mm and 0.1 mm in the Z direction. The sample's post-processing process lasts 2 hours to guarantee the high quality of final 3D printed shellulars. Irregular TPU particles around 60 μm are used as SLS feedstock.

S8.3 Representative samples of shape morphable material with programmable chirality characteristics

Sinterstation 2500 with 50W CO2 laser is used to 3D print TPU materials (SLS Technology).

S8.4 Tensigirity-like multistable material with programmable chirality characteristics

DLP (digital light processing) technology is used to print black photopolymer resin for manufacturing parts. Then parts are assembled and stabilized with one string. B9C 3D printer is used for manufacturing.

References

1. Gorissen, B., Melancon, D., Vasios, N., Torbati, M. & Bertoldi, K. Inflatable soft jumper inspired by shell snapping. *Sci. Robot.* **5**, 1–8 (2020).
2. Shan, S. *et al.* Multistable Architected Materials for Trapping Elastic Strain Energy. *Adv. Mater.* **27**, 4296–4301 (2015).
3. Chen, T., Pauly, M. & Reis, P. M. A reprogrammable mechanical metamaterial with stable memory. *Nature* **589**, 386–390 (2021).
4. Shi, J., Mofatteh, H., Mirabolghasemi, A., Desharnais, G. & Akbarzadeh, A. Programmable Multistable Perforated Shellular. *Adv. Mater.* **2102423**, 2102423 (2021).
